# Polycystin-1 is required for insulin-like growth factor 1-induced cardiomyocyte hypertrophy

**DOI:** 10.1371/journal.pone.0255452

**Published:** 2021-08-18

**Authors:** Carolina Fernández, Natalia Torrealba, Francisco Altamirano, Valeria Garrido-Moreno, César Vásquez-Trincado, Raúl Flores-Vergara, Camila López-Crisosto, María Paz Ocaranza, Mario Chiong, Zully Pedrozo, Sergio Lavandero

**Affiliations:** 1 Faculty of Chemical & Pharmaceutical Sciences & Faculty of Medicine, Advanced Center for Chronic Diseases (ACCDiS), Universidad de Chile and Pontificia Universidad Católica de Chile, Santiago de Chile, Chile; 2 Laboratory of Tumour Resistance, Institute of Biotechnology of the Czech Academy of Sciences, Vestec, Czech Republic; 3 Division of Cardiology, Department of Internal Medicine, University of Texas Southwestern Medical Center, Dallas, Texas, United States of America; 4 Department of Cardiovascular Sciences, DeBakey Heart & Vascular Center Houston Methodist Research Institute, Houston, Texas, United States of America; 5 Department of Cardiothoracic Surgery, Weill Cornell Medical College, Cornell University, Ithaca, New York, United States of America; 6 Facultad de Medicina, Programa de Fisiología y Biofísica, Instituto de Ciencias Biomédicas, Universidad de Chile, Santiago de Chile, Chile; 7 Faculty of Medicine, Division of Cardiovascular Diseases, Pontificia Universidad Católica de Chile, Santiago de Chile, Chile; 8 Center for New Drugs for Hypertension (CENDHY), Pontificia Universidad Católica de Chile, Santiago de Chile, Chile; 9 Corporación Centro de Estudios Científicos de las Enfermedades Crónicas (CECEC), Santiago de Chile, Chile; Tohoku University, JAPAN

## Abstract

Cardiac hypertrophy is the result of responses to various physiological or pathological stimuli. Recently, we showed that polycystin-1 participates in cardiomyocyte hypertrophy elicited by pressure overload and mechanical stress. Interestingly, polycystin-1 knockdown does not affect phenylephrine-induced cardiomyocyte hypertrophy, suggesting that the effects of polycystin-1 are stimulus-dependent. In this study, we aimed to identify the role of polycystin-1 in insulin-like growth factor-1 (IGF-1) signaling in cardiomyocytes. Polycystin-1 knockdown completely blunted IGF-1-induced cardiomyocyte hypertrophy. We then investigated the molecular mechanism underlying this result. We found that polycystin-1 silencing impaired the activation of the IGF-1 receptor, Akt, and ERK1/2 elicited by IGF-1. Remarkably, IGF-1-induced IGF-1 receptor, Akt, and ERK1/2 phosphorylations were restored when protein tyrosine phosphatase 1B was inhibited, suggesting that polycystin-1 knockdown deregulates this phosphatase in cardiomyocytes. Moreover, protein tyrosine phosphatase 1B inhibition also restored IGF-1-dependent cardiomyocyte hypertrophy in polycystin-1-deficient cells. Our findings provide the first evidence that polycystin-1 regulates IGF-1-induced cardiomyocyte hypertrophy through a mechanism involving protein tyrosine phosphatase 1B.

## Introduction

Cardiac hypertrophy is a biological response that imposes an increased demand on myocardial function [1]. Depending on the type and duration of the stimulus, hypertrophy may be physiological or pathological [1]. Cardiomyocyte hypertrophy is characterized by cell enlargement, metabolic changes, increased protein synthesis, and fetal gene expression [2–6]. Mechanical stretch and certain neurohumoral factors, such as insulin-like growth factor (IGF-1), norepinephrine, and angiotensin II, are common cardiomyocyte hypertrophic inducers [2–6].

IGF-1 participates in the physiological cardiac hypertrophy evoked by physical exercise [7]. IGF-1 binds to its receptor (IGF-1R) to induce tyrosine autophosphorylation and activates several downstream signaling pathways, including the extracellular signal-regulated kinase (ERK) and phosphatidylinositol-3 kinase (PI3-K)/protein kinase B (Akt) pathways [8]. IGF-1R activity is mainly modulated by dephosphorylation, which is catalyzed by protein tyrosine phosphatase 1B (PTP1B) [9–11]. PTP1B also plays key roles in metabolic regulation [12,13], cardiac hypertrophy [14] and heart failure [15].

We recently determined that polycystin-1 (PC1), encoded by the *Pkd1* gene, is a critical regulator of cardiomyocyte hypertrophy elicited by mechanical stretch [16]. PC1 is a plasma membrane protein expressed in various tissues that has a large N-terminal extracellular domain and a short cytoplasmic C-terminal domain and modulates several pathways, such as calcineurin/nuclear factor of activated T-cells (NFAT) and Signal transducer and activator of transcription 6 (STAT6) [17]. PC1 also interacts with various membrane proteins, including cell-cell communication proteins [18,19], cell-matrix communication proteins [20], the inositol trisphosphate receptor (IP3R) [21], serine/threonine phosphatase 1 alpha protein [22] and tyrosine phosphatases [23]. Recently, we identified PC1 as a mechanosensor in cardiomyocytes that governs L-type Ca^2+^ channel protein stability [16]. PC1 is crucial for myocardial function, as well as mechanisms involving mechanical stretch (*in vitro*) and pressure overload (*in vivo*) that may induce cardiomyocyte hypertrophy [16]. However, our previous data show that PC1 is not necessary for phenylephrine-dependent cardiomyocyte hypertrophy, suggesting that PC1 activity is stimulus-dependent. Whether PC1 is a general mediator of cardiomyocyte hypertrophy or a mediator of IGF-1-induced cardiomyocyte hypertrophy has not been previously studied. Here, we describe the role of PC1 in IGF1-induced cardiomyocyte hypertrophy. Our results show that PC1 knockdown negatively regulates the IGF-1R, Akt, and ERK signaling pathways, preventing IGF-1-induced cardiomyocyte hypertrophy. Finally, we provide evidence of PTP1B dysregulation upon PC1 knockdown.

## Materials and methods

### Reagents and antibodies

Antibodies against phospho-IGF-1R β (Tyr1135/1136)/insulin receptor β subunit (Tyr1150/1151) (#3024S), the IGF-IR β subunit (#3027S), insulin receptor β (#3025S), phospho-Akt (Ser473) (#9271S), Akt (#9272S) and phospho-ERK1/2 (Thr202/Tyr204) (#9106S) were obtained from Cell Signaling (Danvers, MA). Anti-PC-1 (sc-25570), anti-ERK (sc-94), and CinnGEL 2-methyl ester were purchased from Santa Cruz Biotechnology (Dallas, TX). Anti-β-myosin heavy chain (β-MHC) (VP-M667) was purchased from Vector Laboratories (Burlingame, CA). Peroxidase-conjugated rabbit and mouse anti-IgG secondary antibodies, Hank’s medium, DME medium (DMEM), 199 medium (M199), pancreatin, gelatin, Triton X-100, 5-bromo-2´-deoxyuridine, insulin, anti-GAPDH (G9545), PC-1 and MISSION^®^ siRNA universal negative control #1 (Scr) were purchased from Millipore Sigma (Burlington, MA). SDS-PAGE materials and PVDF membranes were from Bio-Rad Laboratories (Hercules, CA). DAKO fluorescence mounting medium was purchased from Agilent Technologies, Inc. (Santa Clara, CA). All other organic and inorganic reagents, salts, acids, and solvents were purchased from Merck (Darmstadt, Germany), unless otherwise specified.

### Animals

All animals were handled according to the guidelines stated in the Guide for the Care and Use of Laboratory Animals published by the U.S. National Institutes of Health (NIH Publication, 8th Edition, 2011). The protocol was approved by the Institutional Ethics Review Committee of the Faculty of Chemical and Pharmaceutical Sciences, Universidad de Chile. We used Sprague-Dawley rat pups on postnatal days 1–3 to isolate neonatal rat ventricular myocytes (NRVM).

### Cardiomyocyte cultures

NRVM were isolated from neonatal Sprague-Dawley rats, as previously described [8]. In brief, pups were quickly decapitated without the need for anesthesia, hearts were obtained, and cells from neonatal rat ventricles were dissociated in a solution of collagenase (0.2 mg/mL) and pancreatin (1.2 mg/mL). After enzymatic digestion, the cells were plated in gelatin-coated plastic dishes and cultured in DMEM/M199 (4:1) containing 10% fetal bovine serum, 5% fetal calf serum, penicillin-streptomycin solution (100 U/mL-100 μg/mL) and bromodeoxyuridine (100 μM). NRVM (1x10^6^ cells) were plated on gelatin-pre-coated 35-mm dishes. For microscopy experiments, cells were plated on gelatin-pre-coated 18-mm glass coverslips in 12-well plates. NRVM were 95% pure, as assessed by β-MHC immunofluorescence.

### Cardiomyocyte transfection

Small interfering RNAs (siRNA) for PC1 and scrambled (Scr; negative control) siRNA were used according to the manufacturer’s instructions. NRVM were cultured and transfected with siRNAs (120 nM) with Oligofectamine^™^ or Lipofectamine RNAiMax. After 12 h of incubation, the medium was removed and replaced with DMEM/M199. Experiments were performed 24 h after siRNA transfection. Knockdown efficiency was determined by Western blot after 48 h.

### Treatments

NRVM were treated with IGF-1 (10 nM) to study both hypertrophy (48 h) and the IGF-1R signaling pathway (5 min). When using phosphatase inhibitors, NRVM were treated with the inhibitors 1 h prior to IGF-1 stimulation and were maintained throughout the experiment. CinnGEL was dissolved in 0.05% DMSO, and the same solution without CinnGEL was used as a control condition.

### Western blot analysis

NRVM were rinsed three times with ice-cold PBS solution and lysed in cold lysis buffer containing Tris-HCl (100 mM, pH 7.4), NaCl (300 mM) and Nonidet P-40 (0.5% v/v), supplemented with commercial protease and phosphatase inhibitors (Roche Applied Science, Penzberg, Germany). Lysates were spun for 10 min at 12,000 x g using a refrigerated microcentrifuge. Supernatant protein content was assessed using the Bradford method (BioRad protein assay, BioRad, Hercules, CA). Proteins were separated by SDS-PAGE (8–15% polyacrylamide gels) and transferred onto PVDF membranes. Membranes were blocked with 5% nonfat milk in Tris-buffered saline, pH 7.6, containing 0.1% (v/v) Tween 20 (TBST), incubated with primary antibodies (1:1,000), and re-blotted with horseradish peroxidase-linked secondary antibody (1:5000). The antigen-antibody reaction was detected using an EZ-ECL kit (Biological Industries, Kibbutz Beit Haemek, Israel), and blots were quantified by image densitometry using the ImageJ software (NIH, USA). GAPDH intensities were used as a loading control.

### RNA purification and real-time quantitative reverse transcription-PCR (qRT-PCR)

Total RNA was isolated using TRIzol, according to the manufacturer’s protocol (Invitrogen, Carlsbad, CA). Random hexamers were used for reverse transcription reactions using Superscript II (Invitrogen, Carlsbad, CA). qRT-PCR was performed using Master mix PowerUp SYBR Green (Applied Biosystems, Carlsbad, CA), and results were analyzed using the Step One^™^ system (Thermo Fisher Scientific, MA; USA). Data for each transcript were normalized to Pabpn1 mRNA or 18S rRNA as an internal control, using the 2^-ΔΔCT^ method. Primer sequences were: brain natriuretic peptide (*BNP)* forward: 5’-TCCTTAATCTGTCGCCGCTG-3’, reverse: 5’-AGGCGCTGTCTTGAGACCTA-3’; *Pabpn1* forward: 5’-GTTGGCAATGTGGACTATGG-3’, reverse: 5’-AACAGGGACTCATCTAAGGC-3’. Polycystic kidney disease 1 (*Pkd1*) and 18S rRNA sequences were described before [16].

### Cell morphology and sarcomerization

After treatments, cells were fixed with paraformaldehyde and permeabilized with Triton X-100. Rhodamine-phalloidin was used to stain F-actin (1:500, Thermo Fisher Scientific, MA) [24]. Cardiomyocyte hypertrophy, area, perimeter, the percentage of sarcomerization and sarcomere fluorescence profile were calculated from fluorescent images obtained using an epifluorescence microscope (Carl Zeiss Axiovert 135, LSM Microsystems). At least 50 cells from randomly selected fields were analyzed using the ImageJ software (NIH, USA).

### [^3^H]-leucine incorporation

Cardiomyocytes were incubated with [^3^H]-leucine (1 mCi/mL; Perkin-Elmer). After treatment, cardiomyocytes were rinsed three times with ice-cold PBS, incubated with 10% trichloroacetic acid (30 min, 4°C), and rinsed with ice-cold 95% ethanol. Remaining proteins were then dissolved in 0.5 M NaOH (6 h, 37°C) with gentle agitation. Protein extracts were neutralized with 0.5 M HCl. Total content from each well was processed by scintillation counting (Beckman Coulter Life Sciences, Indianapolis, IN).

### Statistical analysis

Depending on the type of experiment, results are shown as representative images or mean ±SD of at least three independent experiments. The Mann-Whitney-U or Kruskal-Wallis tests were used for data analysis. A p value <0.05 was set as the level of statistical significance.

## Results

### PC1 knockdown blunts IGF-1-induced cardiomyocyte hypertrophy

**Fig 1:**
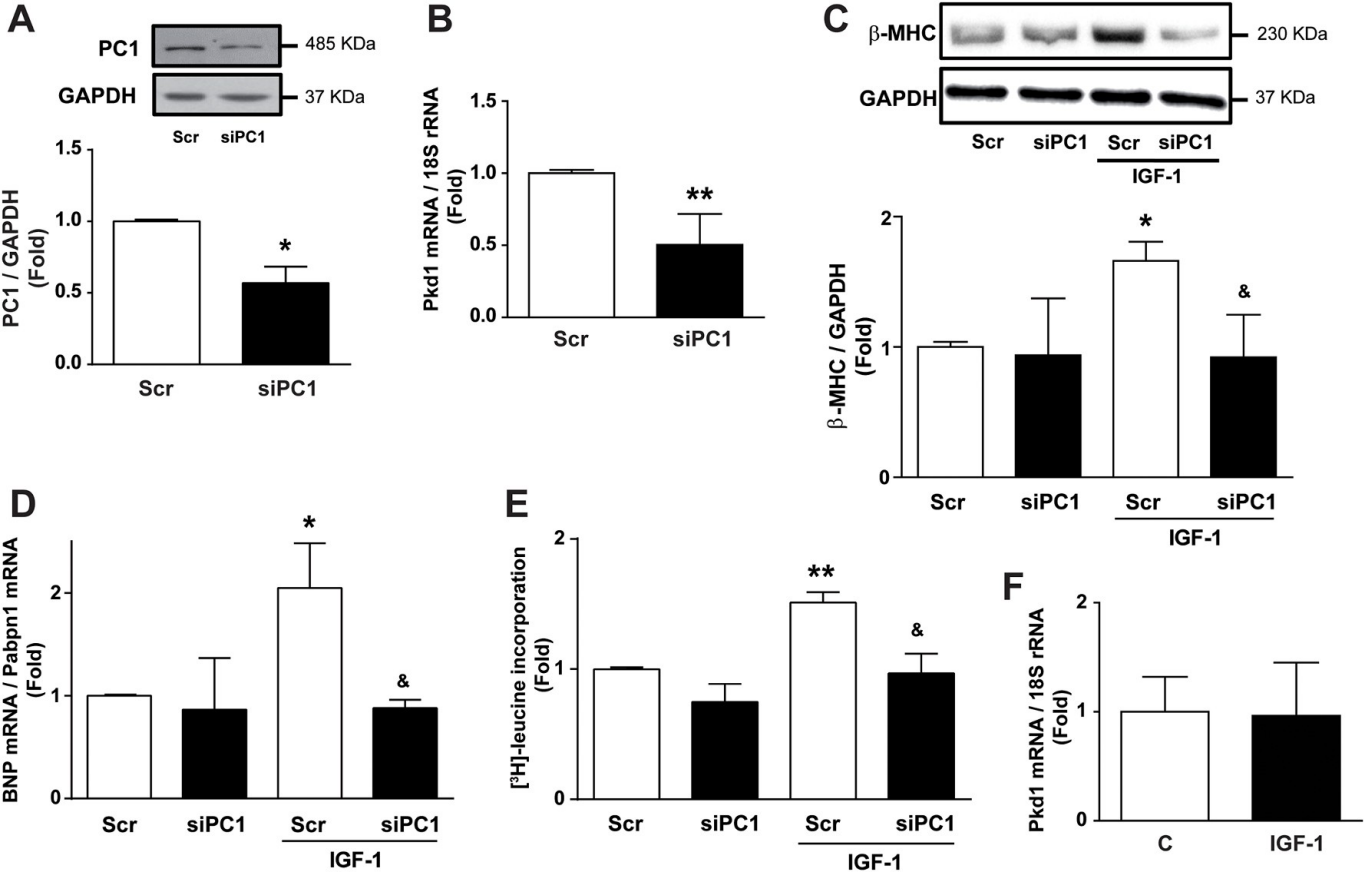
PC1 is required for IGF-1-induced cardiomyocyte hypertrophy. Cardiomyocytes were transfected with scrambled (Scr) or PC1 siRNA (siPC1), incubated for 24 h, and then treated with IGF-1 (10 nM) or vehicle for 24 h. (A) Representative Western blot of polycystin-1 (PC1) and GAPDH as a loading control. The lower panel shows the PC1/GAPDH ratio. (B) Bar graph of polycystic kidney disease 1 (*Pkd1*) mRNA levels. (C) Upper panel shows representative Western blots of β-MHC and GAPDH. The graph in the lower panel shows the β-MHC/GAPDH ratio calculated by densitometric analysis. (D) Brain natriuretic peptide *(BNP*) mRNA levels determined by qRT-PCR are expressed relative to Pabpn1 mRNA. (E) [^3^H]-leucine incorporation in cardiomyocytes. (F) Bar graph shows *Pkd1* mRNA levels after IGF-1 stimulus. Values are shown as mean ± SD (n = 3-6). *p<0.05 and **p<0.01 vs. Scr, ^&^p<0.05 vs. Scr+IGF-1.

To elucidate whether PC1 is required for IGF-1-dependent cardiomyocyte hypertrophy, PC1 expression was reduced in neonatal rat ventricular myocyte (NRVM) using specific siRNA constructs (siPC1) and scrambled (Scr) siRNA as a negative control. In our previous work, we tested two different siRNAs to knockdown PC1 in NRVM. Both siRNAs decreased PC1 expression in the same level [16], so in this work, we used only one of them. siPC1 decreased PC1 protein content by 50% compared to the control after 24 h of transfection (Fig 1A) and the *Pkd1* mRNA levels (Fig 1B). To determine hypertrophy *in vitro*, we measured the following markers: β-myosin heavy chain (MHC) protein expression, brain natriuretic peptide (BNP) mRNA levels, and [^3^H]-leucine incorporation. Interestingly, PC1 knockdown reduced IGF-1 dependent increases of β-MHC protein content, BNP mRNA abundance, and [^3^H]-leucine incorporation (Fig 1C–1E). Moreover, IGF-1 did not change *Pkd1* mRNA levels in cardiomyocytes (Fig 1F), suggesting that IGF-1 does not alter PC1 expression.

**Fig 2:**
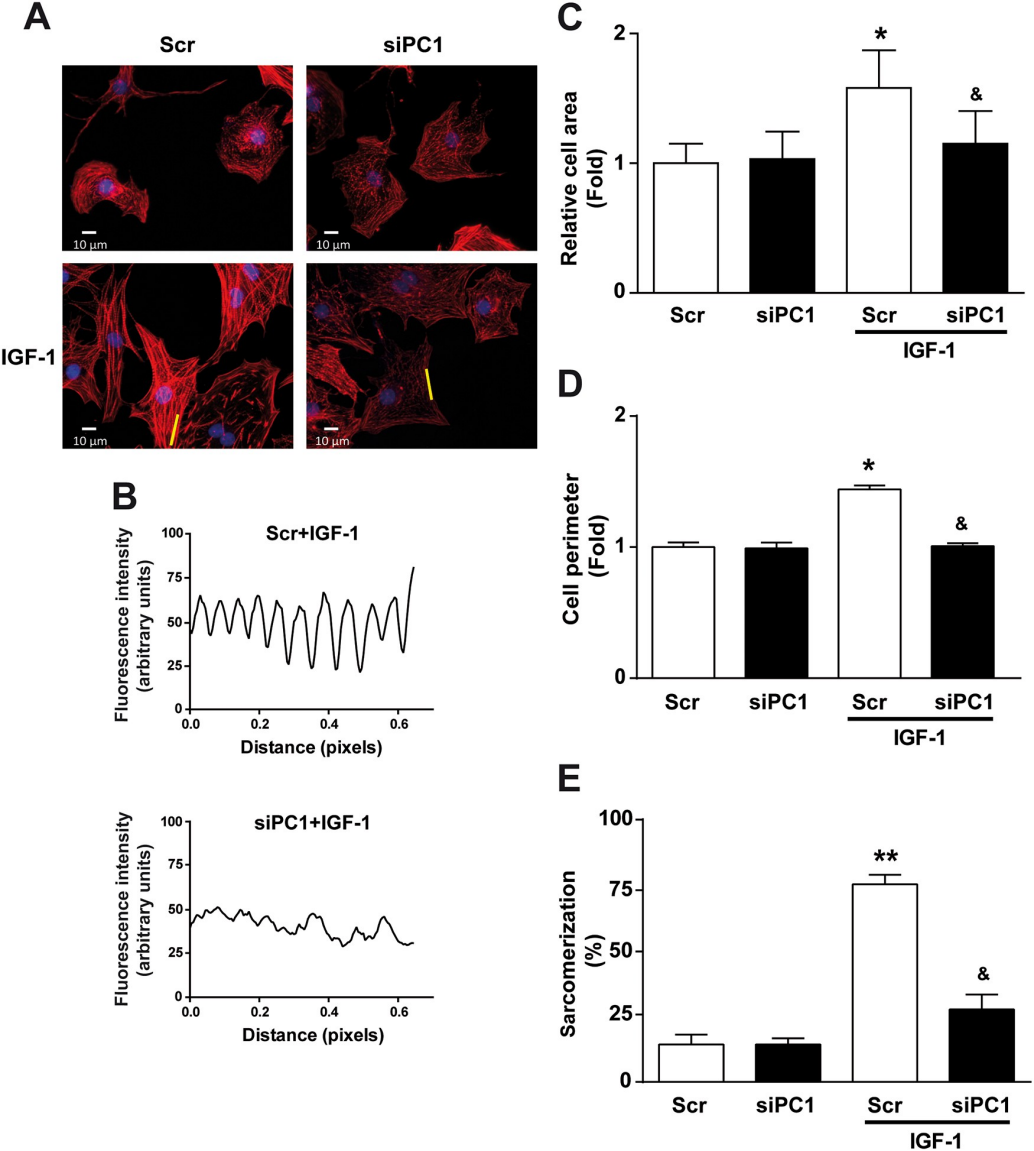
IGF-1-induced hypertrophic changes in cardiomyocyte morphology are mediated by PC1. Cardiomyocytes were transfected with scrambled (Scr) or PC1 siRNA (siPC1), incubated for 24 h, and then treated with IGF-1 (10 nM) or vehicle for 24 h. (A) Representative images of cardiomyocytes stimulated with IGF-1 and stained with rhodamine-phalloidin. (B) Fluorescence densitometric profiles of the lines depicted in (A), showing sarcomere density in IGF-1-stimulated cardiomyocytes pretreated with Scr or siPC1. Bar graphs show cardiomyocyte (C) cell area, (D) cell perimeter and (E) sarcomerization. Values are shown as mean ± SD (n = 3–6). *p<0.05 and **p<0.01 vs. Scr, ^&^p<0.05 vs. Scr+IGF-1.

*In vitro* cardiomyocyte hypertrophy can be defined phenotypically as an increase in cellular area, perimeter, and the degree of sarcomerization. NRVM have a very rudimentary sarcomeric structure, which turns into a highly organized “stair-like” structure after a pro-hypertrophic stimulus [24–26]. IGF-1 treatment increased cardiomyocyte area, perimeter, and percentage of cells with highly organized sarcomeres (Fig 2A and 2B), while PC1 knockdown completely prevented these IGF-1 dependent changes (Fig 2C–2E). Taken altogether, these results suggest that PC1 is required for IGF-1-induced cardiomyocyte hypertrophy.

### PC1 regulates the IGF-1 receptor signaling pathway

**Fig 3:**
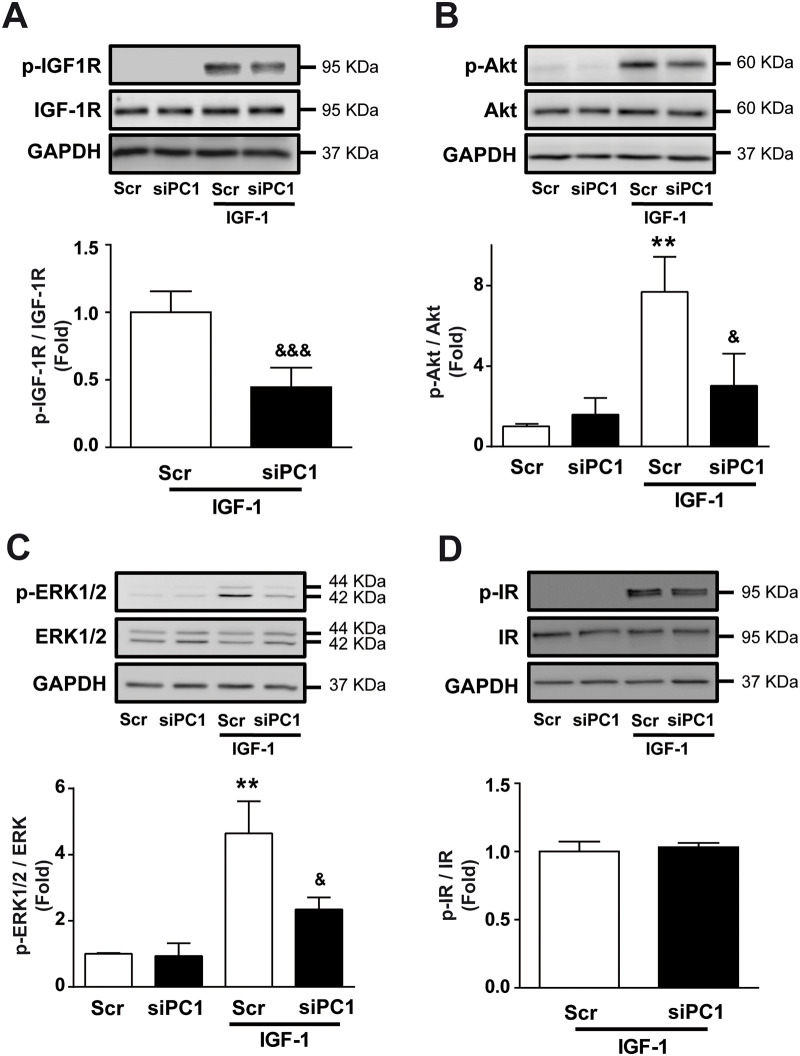
PC1 regulates IGF-1-dependent IGF-1R activation. Cardiomyocytes were transfected with scrambled (Scr) or PC1 siRNA (siPC1), incubated for 24 h, and then stimulated with IGF-1 (10 nM) or vehicle for 5 min. Upper panels are representative Western blots of (A) phosphorylated IGF-1R (p-IGF-1R) and total IGF-1R, (B) phosphorylated Akt (p-Akt) and total Akt, (C) phosphorylated ERK1/2 (p-ERK1/2) and total ERK1/2 and (D) phosphorylated insulin receptor (p-IR) and total IR. Lower panels show the (A) p-IGF1R/IGF-1R, (B) p-Akt/Akt, (C) p-ERK1/2/ERK1/2, and (D) p-IR/IR ratios. GAPDH was used as a loading control. Values are shown as mean ± SD (n = 3–6). **p<0.01 vs. Scr, ^&^p<0.05 and ^&&&^p<0.001 vs. Scr+IGF-1.

IGF-1 activates its receptor (IGF-1R) by inducing tyrosine autophosphorylation, subsequently activating downstream signaling pathways such as Akt and ERK1/2 [27–29]. It is well known that IGF-1 exerts its pro-hypertrophic effects via the Akt and ERK1/2 signaling pathways [27,29]. To study the role of PC1 in the activation of IGF-1R and its downstream signaling pathways, we measured the phosphorylation status of IGF-1R, ERK1/2, and Akt upon IGF-1 stimulation. IGF-1 increased IGF-1R phosphorylation at 5 min, and PC1 knockdown partially prevents this activation (Fig 3A). Total IGF-1R content did not change upon PC1 knockdown in NRVM (Fig 3A). Next, we measured both Akt and ERK1/2 activation in NRVM after IGF-1 treatment. PC1 knockdown reduced Akt (Fig 3B) and ERK1/2 (Fig 3C) phosphorylation upon IGF-1 treatment. In order to evaluate whether these changes were specific for the IGF-1 receptor, we assessed insulin receptor (IR) activation upon insulin stimulation. Insulin triggered similar IR phosphorylation levels in NRVM transfected with either Scr or siPC1 (Fig 3D). We did not detect IGF-1R or IR phosphorylation in basal condition. Taken together, these results suggest that PC1 has a specific effect on the IGF-1R signaling pathway in cardiomyocytes.

### PC1 regulates IGF-1R activation through protein tyrosine phosphatase 1B

**Fig 4:**
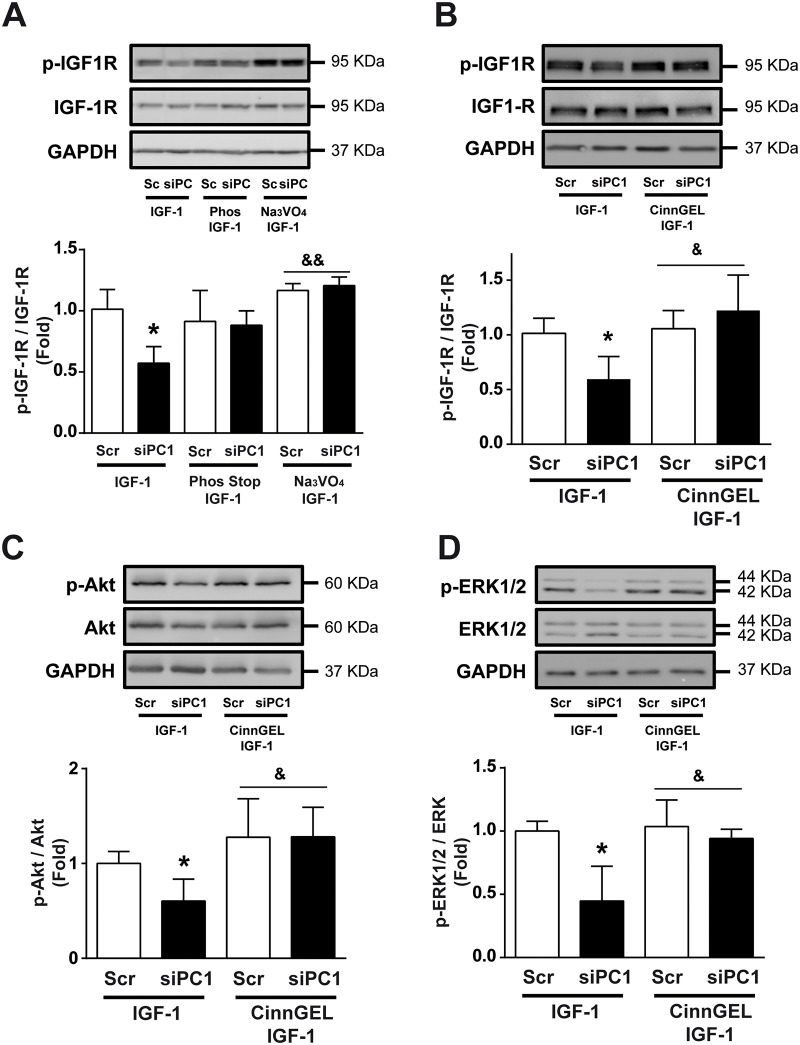
PC1 requires PTP1B to regulate IGF-1-dependent IGF-1R activation. Cardiomyocytes were transfected with scrambled (Scr) or PC1 siRNA (siPC1), incubated for 24 h, and then stimulated with IGF-1 (10 nM) in the presence or absence of the phosphatase inhibitors Phos Stop (1X), Na_3_VO_4_ (1 mM) or CinnGEL (10 μM) for 5 min. Upper panels are representative Western blots of (A and B) phosphorylated IGF-1R (p-IGF1R) and IGF-1R, (C) phosphorylated Akt (p-Akt) and total Akt and (D) phosphorylated ERK1/2 (p-ERK1/2) and total ERK1/2. Lower panels show the (A-B) p-IGF-1R/IGF1R, (C) p-Akt/Akt, and (D) p-ERK1/2/ERK1/2 ratios. GAPDH was used as a loading control. Values are shown as mean ± SD (n = 3–6). *p<0.05 vs. Scr+IGF-1, ^&^p<0.05, ^&&^p<0.01 vs. siPC1+ IGF-1.

Tyrosine phosphatases fine-tune tyrosine kinase receptor function, modulating their phosphorylation status [30,31]. Some evidence suggests that PC1 interacts with several isoforms of receptor protein tyrosine phosphatases, forming multimeric complexes that regulate PC1-dependent signaling [23]. Therefore, we hypothesized that PC1 knockdown might impair phosphatase activity. Using the phosphatase inhibitors PhoSTOP and Na_3_VO_4_, we disrupted the inhibitory effect of PC1 knockdown on IGF-1R phosphorylation upon IGF-1 stimulation (Fig 4A). These data suggest that tyrosine phosphatases are involved in the mechanism through which PC1 regulates IGF-1R phosphorylation in NRVM.

Protein tyrosine phosphatase 1B (PTP1B) participates in IGF-1R dephosphorylation and is the key negative regulator of IGF-1 signaling [9,11,30]. CinnGEL 2-methyl ester (10 μM, CinnGEL), a specific inhibitor of PTP1B, completely prevented the decrease of IGF-1R phosphorylation induced by PC1 knockdown upon IGF-1 stimulation in NRVM (Fig 4B). CinnGEL also inhibited the decrease in Akt (Fig 4C) and ERK1/2 (Fig 4D) phosphorylation induced by PC1 knockdown upon IGF-1 stimulation. These results suggest that PC1 regulates IGF-1R activation through a PTP1B-dependent mechanism.

### PC1 knockdown impairs PTP1B activation and IGF-1-induced cardiomyocyte hypertrophy

**Fig 5:**
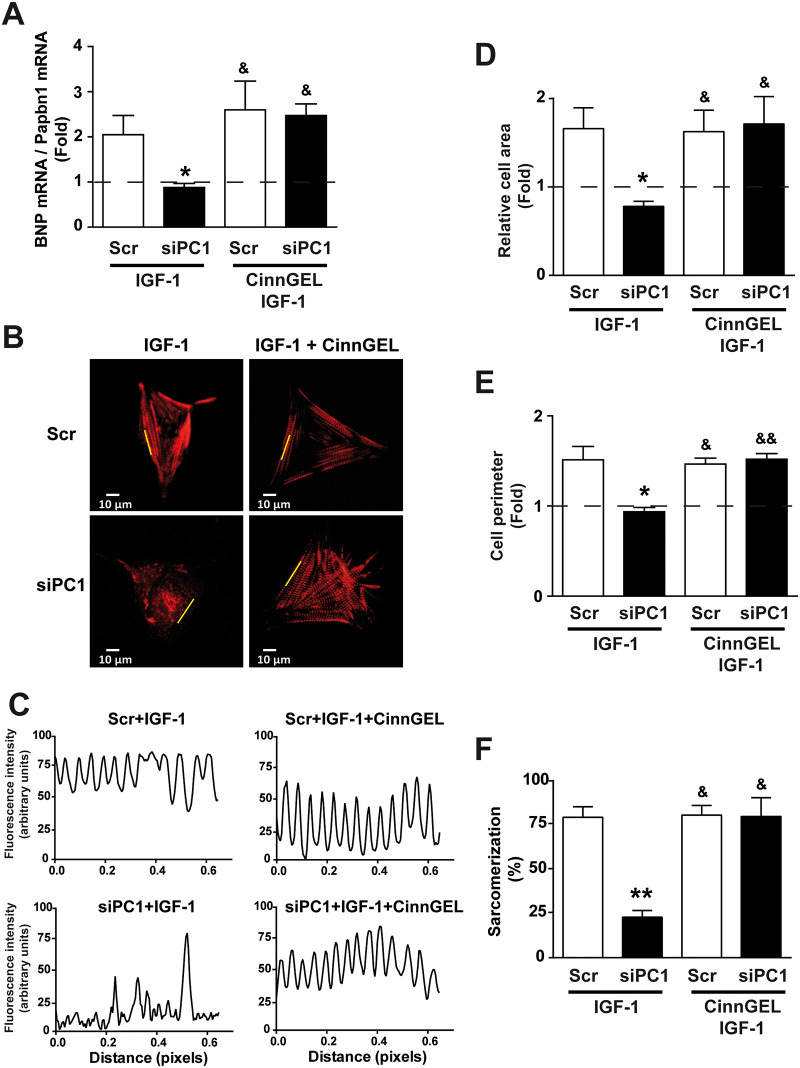
IGF-1-dependent cardiomyocyte hypertrophy requires inhibition of PTP1B by PC1. Cardiomyocytes were transfected with either scrambled (Scr) or PC1 siRNA (siPC1), incubated for 24 h, and then stimulated with IGF-1 (10 nM) in the presence or absence of the PTP1B inhibitor CinnGEL (10 μM). (A) Brain natriuretic peptide (BNP) mRNA levels were expressed relative to Pabpn1 mRNA levels. (B) Representative images of cardiomyocytes stained with rhodamine-phalloidin. (C) Representative fluorescence profile of lines depicted in (B) used for sarcomere densitometric analysis. Measurements of cardiomyocyte (D) cell area, (E) cell perimeter and (F) sarcomerization. The dashed line indicates data normalized respect to the Scr control. Values are shown as mean ± SD (n = 4). *p<0.05 and **p<0.01 vs. Scr+IGF-1, ^&^p<0.05 and ^&&^p< 0.01 vs. siPC1+IGF-1.

To assess the effect of PC1 knockdown on PTP1B activation in IGF-1-induced cardiomyocyte hypertrophy, NRVM were exposed to IGF-1 in the presence or absence of CinnGEL, and hypertrophic parameters were measured. CinnGEL reduced the inhibitory effect of PC1 knockdown on BNP mRNA upon IGF-1 stimulation (Fig 5A). Moreover, morphologic studies and fluorescence intensity profiles (Fig 5B and 5C) confirmed that CinnGEL also prevented the decrease in cell area (Fig 5D), perimeter (Fig 5E), and sarcomerization (Fig 5F) induced by PC1 knockdown upon IGF-1 stimulation. All these data are presented as fold with respect to Scr. These results suggest that PTP1B is involved in PC1-dependent regulation of IGF-1-induced cardiomyocyte hypertrophy.

## Discussion

In this study, we showed that IGF-1-induced cardiomyocyte hypertrophy requires PC1. PC1 knockdown using siRNA blunted the IGF-1-dependent upregulation of hypertrophic β-MHC protein levels, BNP mRNA abundance, [^3^H]-leucine incorporation, sarcomerization, cell area, and perimeter of NRVM. Furthermore, we provided evidence that PC1 knockdown affects the phosphorylation status of IGF-1R depending on PTP1B activity.

IGF-1 participates in the initiation and development of physiological cardiac hypertrophy [32,33], and its cardiomyocyte-specific overexpression leads to cardiac hypertrophy in transgenic mice [7]. IGF-1 is critical for cardiomyocyte survival due to its potent cardioprotective effect [32,33]. Our group and others have shown that IGF-1 activates multiple signal transduction pathways in cardiomyocytes [8,27,28,34]. To our knowledge, this is the first study showing the involvement of PC1 in IGF-1 signaling. Our data suggest that PC1 knockdown specifically regulates the IGF-1 signaling pathway, but without affecting other tyrosine kinase receptors, such as the IR, or participating in phenylephrine-mediated cardiomyocyte hypertrophy [27].

Tyrosine phosphorylation is regulated by phosphatases to terminate the activation of tyrosine kinase receptors. Moreover, regulation of both IGF-1 and insulin signaling involves serine phosphorylation of Insulin receptor substrate 1/2 (IRS1/2) [35]. Insulin- or IGF-1-induced serine phosphorylation of IRS1/2 dissociates these proteins from their receptors, preventing tyrosine phosphorylation and inhibiting binding with downstream effectors. Therefore, this process serves as negative feedback mechanism [35]. Our results showed that PC1 knockdown not only reduces IGF-1-induced Akt and ERK phosphorylation but also decreases IGF-1R phosphorylation. This effect seems to be specific to IGF-1R signaling, as PC1 knockdown did not affect insulin-induced IR phosphorylation in cardiomyocytes. On the other hand, PC1 knockdown reduces only around 50% of IGF-1-dependent IGF-1R phosphorylation, as well as Akt and ERK1/2 phosphorylations. This result could be explained, at least partly, because PC1 siRNA only reduces 50% of PC1 expression in NRVM. However, a significant amount of PC1 remains in the cell in these cells, and the knockdown of PC1 could affect its subcellular distribution or traffic to the plasma membrane, thus explaining the effects on the IGF-1R. We acknowledge as a study limitation that the subcellular location of the remaining PC1 was not studied here. Interestingly, PC1 has been located both in the plasma membrane and other subcellular compartments [36]. In aggregate, our data strongly suggest that PC1 regulates positively IGF-1R activity induced by IGF-1 in NRVM.

Receptor protein tyrosine phosphatases interact with polycystins both in the primary cilia and at adhesion complexes [20,23]. The presence of cilia in cardiomyocytes is still controversial [37–39], and methodological issues to identify cardiac myocytes or cilia could explain these discrepancies. However, independent of the presence of cilia, cardiomyocyte-specific knockdown of PC1 promotes cardiomyocyte alterations [16,40] that are distinct from the ones elicited by Polycystin-2 knockdown [37]. Moreover, we previously showed that NRVM knockdown to PC1 did not change the PC2 expression [41] as well as cardiomyocytes-restricted silencing of PC1 mice model [16], suggesting that our results are not influenced by PC2 alterations. Altogether, this evidence suggests that these proteins play different roles in cardiomyocytes, so we did not explore the role of Polycystin-2 in IGF-1-induced cardiomyocyte hypertrophy.

Interestingly, we found that PTP1B inhibition normalizes IGF-1R phosphorylation and the hypertrophic response upon IGF-1 stimulation in cells lacking PC1. These results suggest that PC1 acts as a negative regulator of PTP1B activity, allowing normal activation of the IGF-1R. IGF-1R can also be dephosphorylated by the tyrosine phosphatase SHP-2 [42]. Therefore, although our data support that PTP1B has a relevant role in IGF-1R dephosphorylation, we cannot rule out the participation of other tyrosine phosphatases in the PC-1-dependent effects. Previous reports have indicated that PC1 can interact and form multiprotein complexes with focal adhesion proteins [18,20], cell-cell *adherens junction* proteins [18,20], the IP3R [21], intermediate filaments [19], serine/threonine protein phosphatase-1α [43] and tyrosine phosphatases [31]. Tyrosine phosphatases also interact with and bind to receptor tyrosine kinases (RTKs) [31]. We hypothesize that PC1 controls at least the activity of PTP1B to regulate IGF-1R activity. Here, we have shown that the use of both general phosphatase inhibitors and specific tyrosine phosphatase inhibitors completely revert the PC1 knockdown-dependent decreases in IGF-1R, Akt and ERK phosphorylation. Therefore, these data suggest that PC1 may regulate the IGF-1 signaling pathway by controlling the function of tyrosine phosphatases. A possible mechanism through PC1 regulates PTP1B activity could involved AKT as a negative modulator of this phosphatase. PC1 stimulates AKT activity [41,44], while PTP1B phosphorylation on Ser(50) by AKT negatively regulates the activity of this phosphatase [45]. Future experiments should address this hypothesis andinvestigate a potential interaction between PTP1B and PC1 with the IGF-1R and whether PC1 regulates PTP1B availability to clarify the mechanism involved in this signaling pathway.

Furthermore, interactions between the IGF-1R and mechanosensors, such as integrins, have also been described [22,46]. This interaction is fundamental for proper IGF-1R signaling in response to IGF-1 in osteoblasts [22]. PC1 is a plasma membrane protein expressed in various tissues. As previously reported, we have identified PC1 as a mechanosensor in cardiomyocytes [16]. Whether PC1 modulates IGF-1R signaling by only regulating phosphatase levels or exerting its control as a mechanoreceptor remains unknown. Moreover, future studies are necessary to determine whether PC1 and PTB1B interact directly or indirectly and will broaden our understanding of this pathway.

## Conclusion

**Fig 6:**
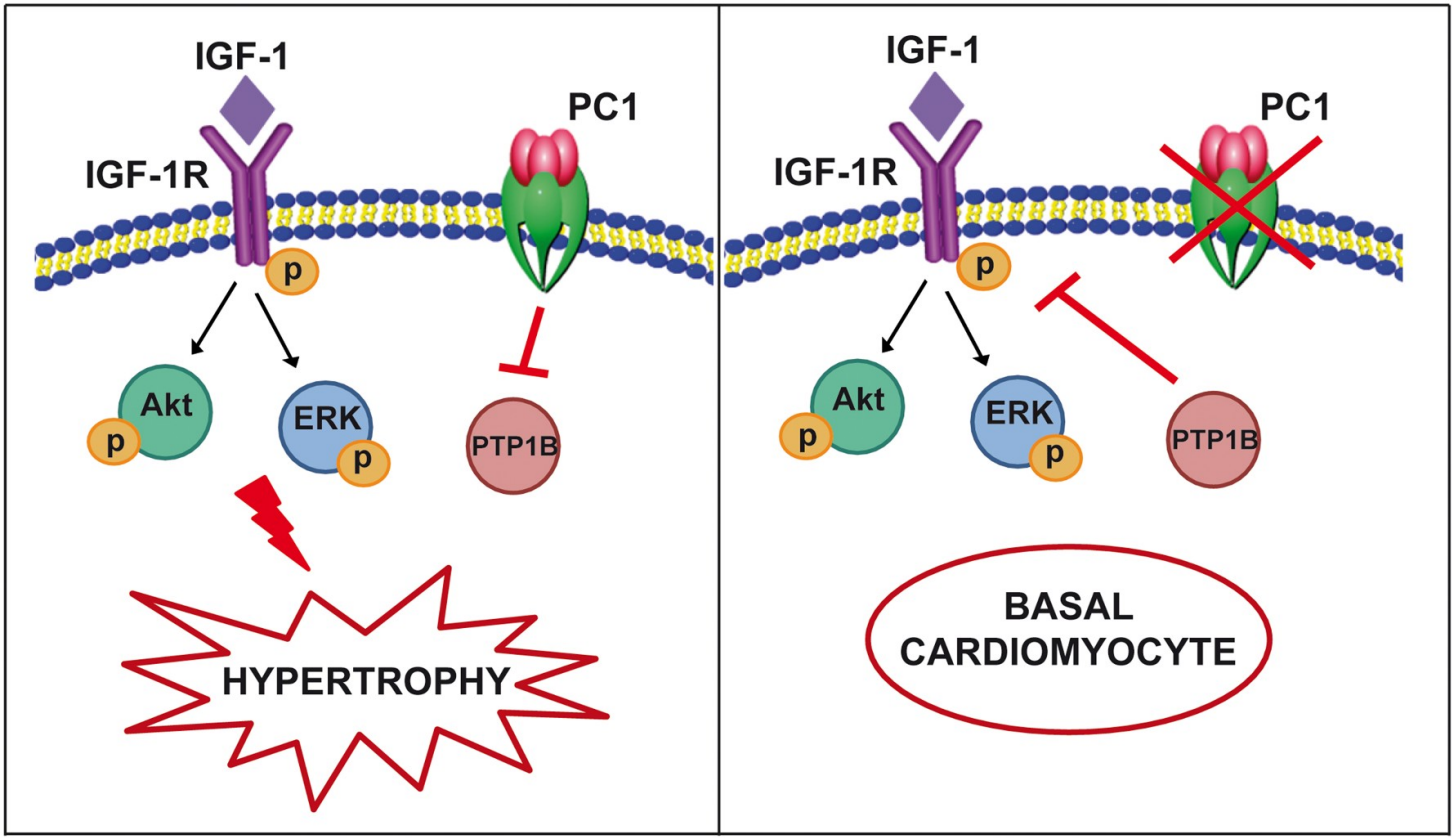
Graphical model of polycystin-1 and IGF-1-induced cardiac hypertrophy regulation.

Our results show for the first time that PC1 is required for IGF-1-dependent induction of cardiomyocyte hypertrophy. PC1 may regulate IGF-1R phosphorylation by controlling the availability of tyrosine phosphatases. A graphical model is shown in Fig 6.

## Supporting information

S1 Fig This is the S1 raw images. (PDF)

S2 Fig This is the S2 controls Figs 4 and 5. (PDF)
