# Supplementary material for: Polycystin-1 is required for insulin-like growth factor 1-induced cardiomyocyte hypertrophy
Source: PLoS One. 2021 Aug 18;16(8):e0255452. doi: 10.1371/journal.pone.0255452 (PMC8372926; doi:10.1371/journal.pone.0255452)
Supplement: S1 Fig — (PDF) [file pone.0255452.s001.pdf]

Fig 1A
n 1

Paper figure

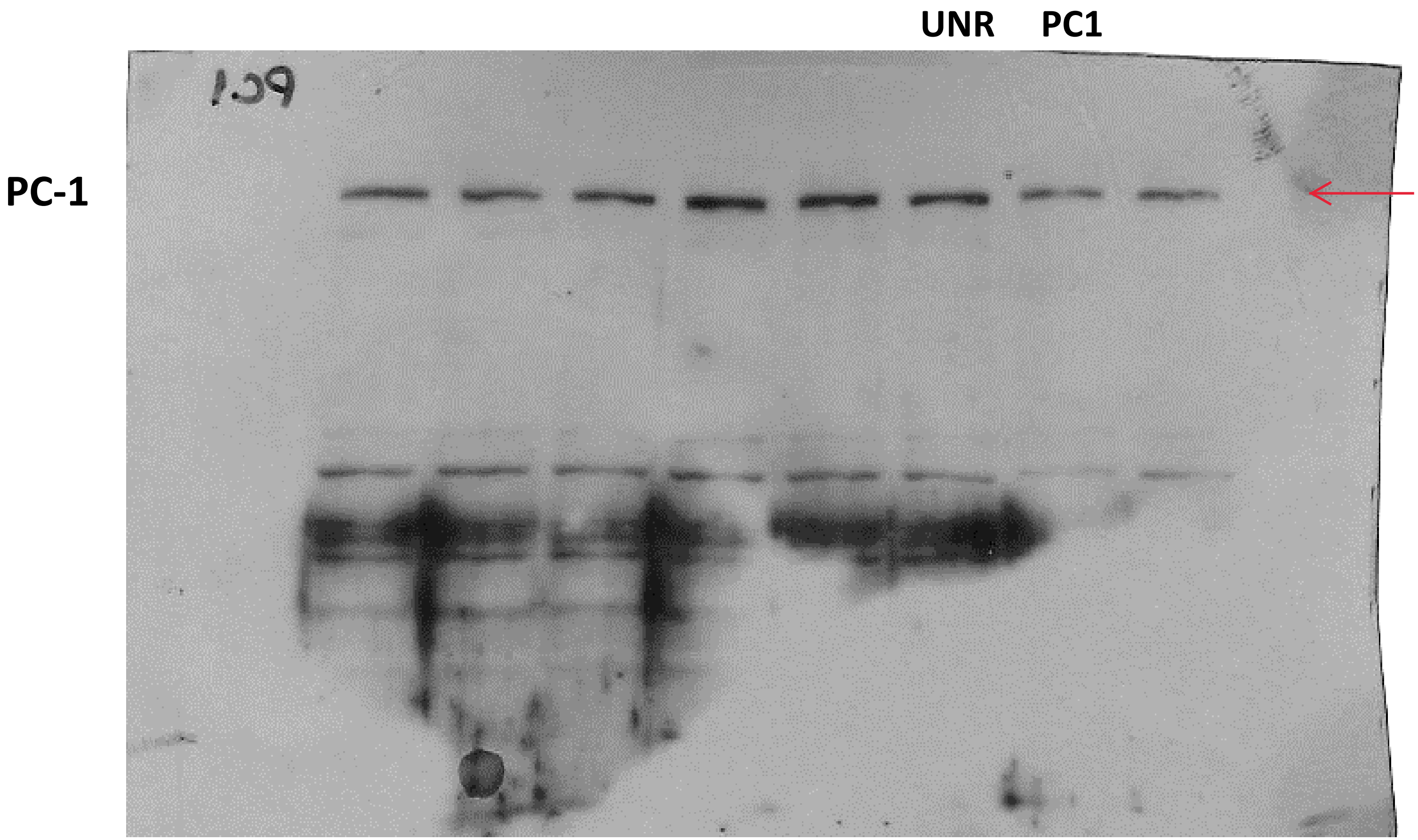

Detection method: EZ-ECL chemiluminescent detection HRP activity, manual development

Fig 1A
n 1
Paper figure

GAPDH

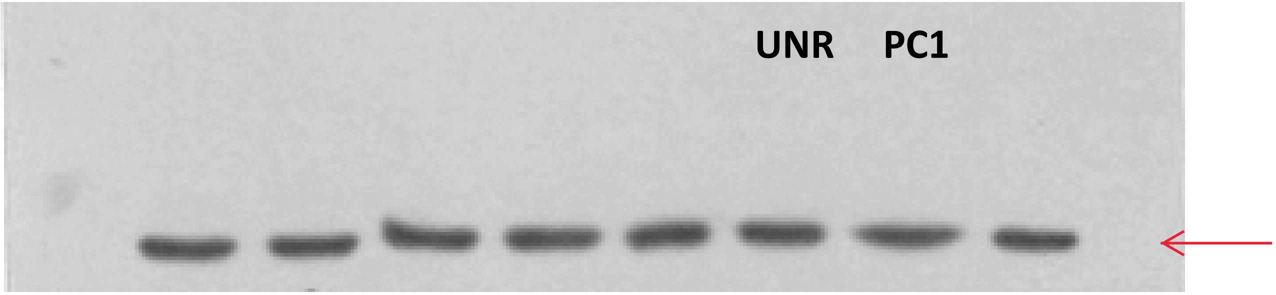

Detection method: EZ-ECL chemiluminescent detection HRP activity, manual development

Fig 1A
n2

UNR PC1

PC-1 antibody

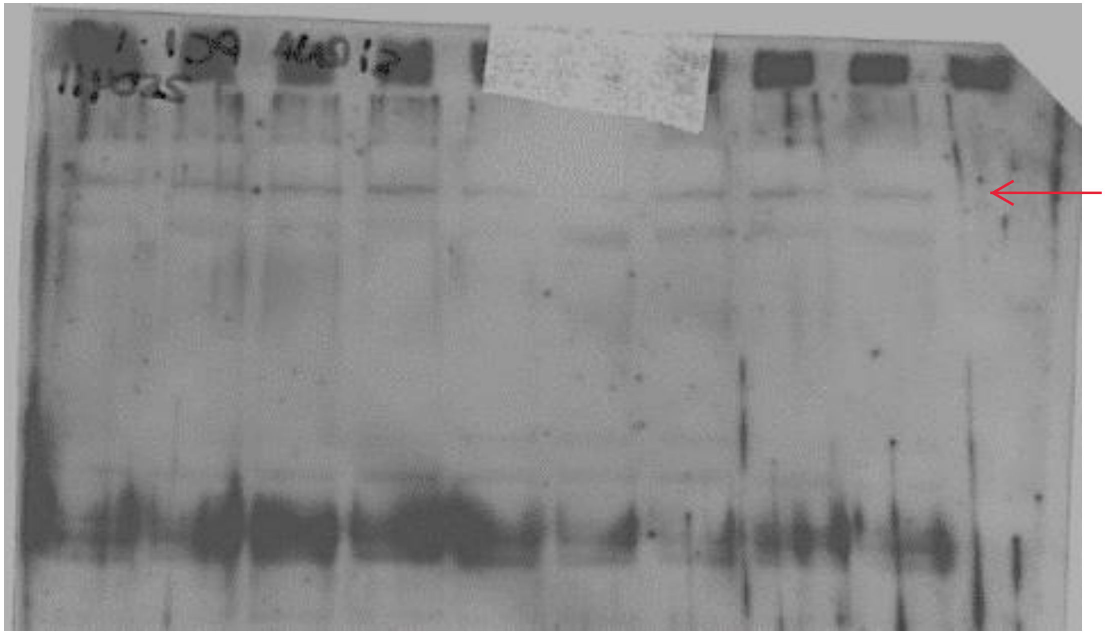

UNR PC1

GAPDH antibody

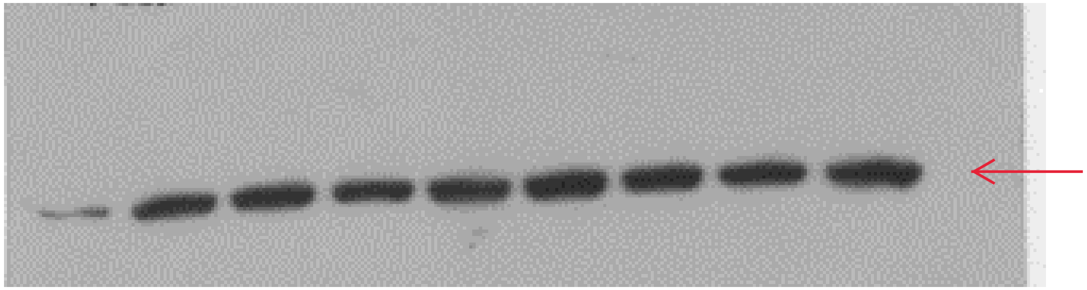

Detection method: EZ-ECL chemiluminescent detection HRP activity, manual development

Fig 1A
n 3

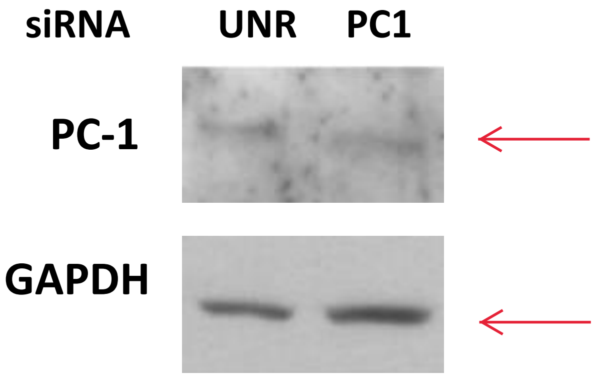

Detection method: EZ-ECL chemiluminescent detection HRP activity, manual development

Fig 1C
n1
Paper figure

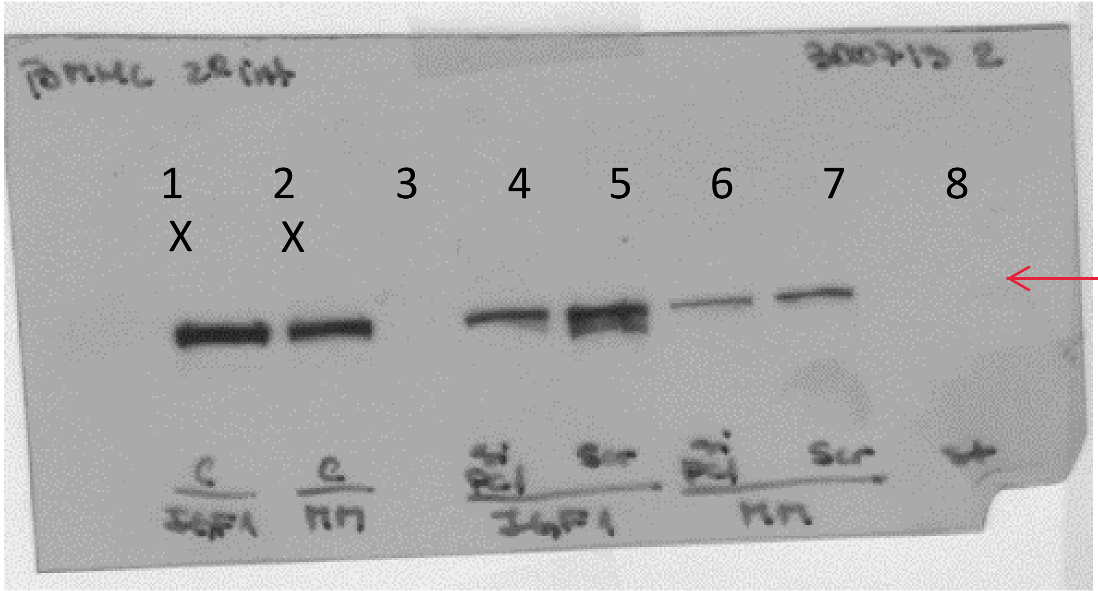

β-MHC antibody

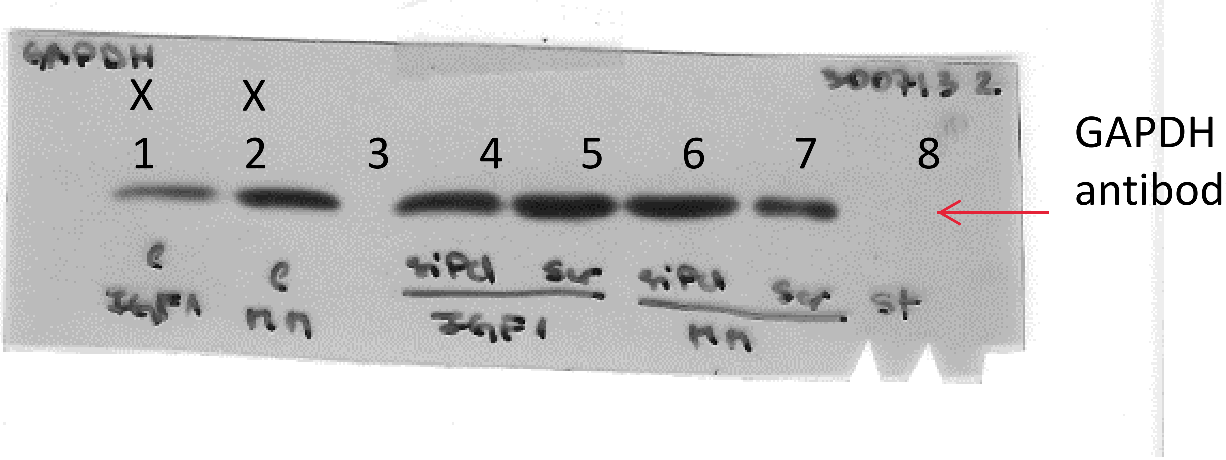

GAPDH antibody

Samples:
1: NRVM IGF-1 stimulated
2: NRVM control medium
3: empty
4: NRVM trated with PC1 siRNA and IGF-1
5: NRVM trated with UNR siRNA and IGF-1
6. NRMV trated with PC1 siRNA and control medium
7: NRMV trated with UNR siRNA and control medium
8: Standard Molecular weight

Detection method: EZ-ECL chemiluminescent detection HRP activity, manual development

Fig 1C
n2

Samples:
1: NRVM IGF-1 stimulated
2: NRVM control medium
3: NRVM trated with UNR siRNA and control medium
4: NRVM trated with PC1 siRNA and IGF-1
5: NRVM trated with UNR siRNA and IGF-1
6. NRMV trated with PC1 siRNA and control medium
7: NRMV trated with UNR siRNA and control medium
8: Standard Molecular weight

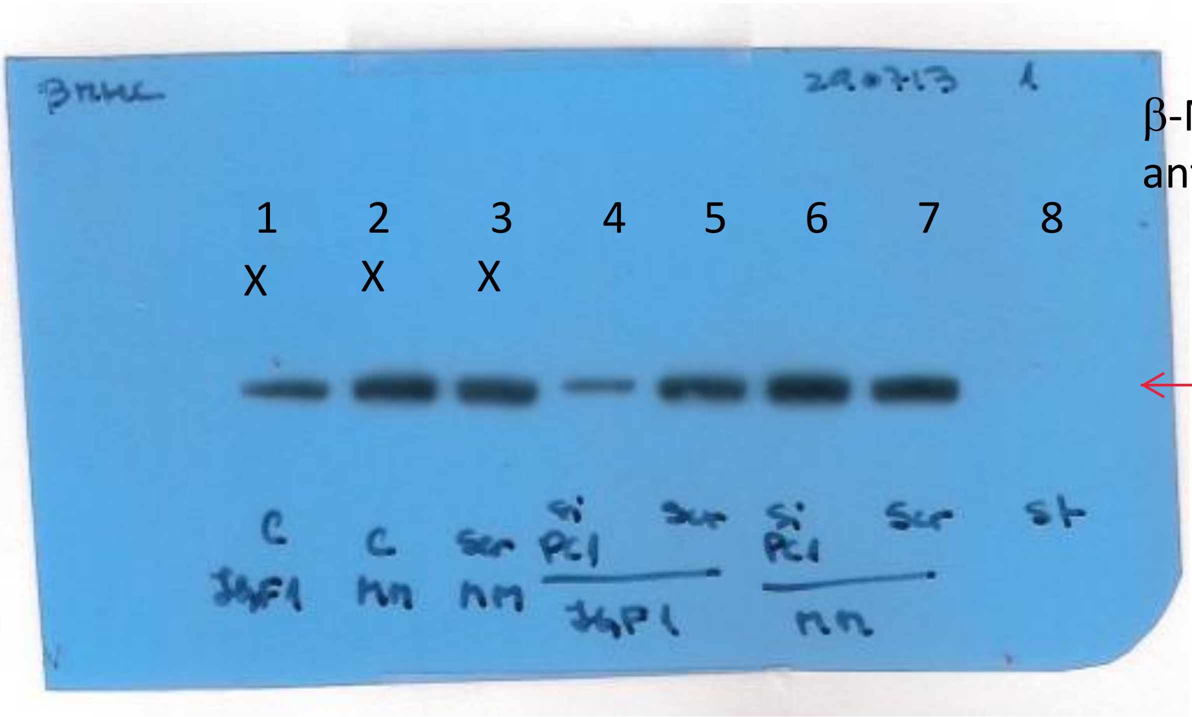

β-MHC antibody

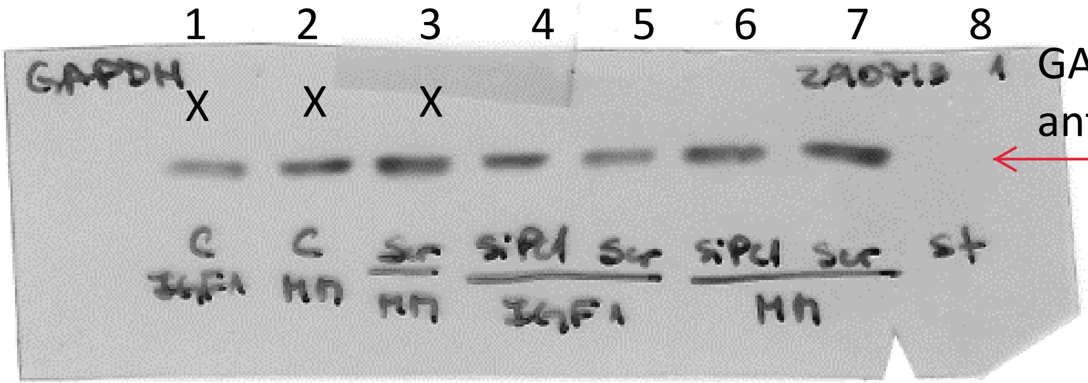

GAPDH antibody

Detection method: EZ-ECL chemiluminescent detection HRP activity, manual development

Fig 1C
n3

Samples:
1: NRVM norepinephrine stimulated
2: NRVM IGF-1 stimulated
3: NRVM trated control medium
4: NRVM trated with PC1 siRNA and IGF-1
5: NRVM trated with UNR siRNA and IGF-1
6. NRMV trated with PC1 siRNA and control medium
7 and 8: NRMV trated with UNR siRNA and control medium
9: Standard Molecular weight

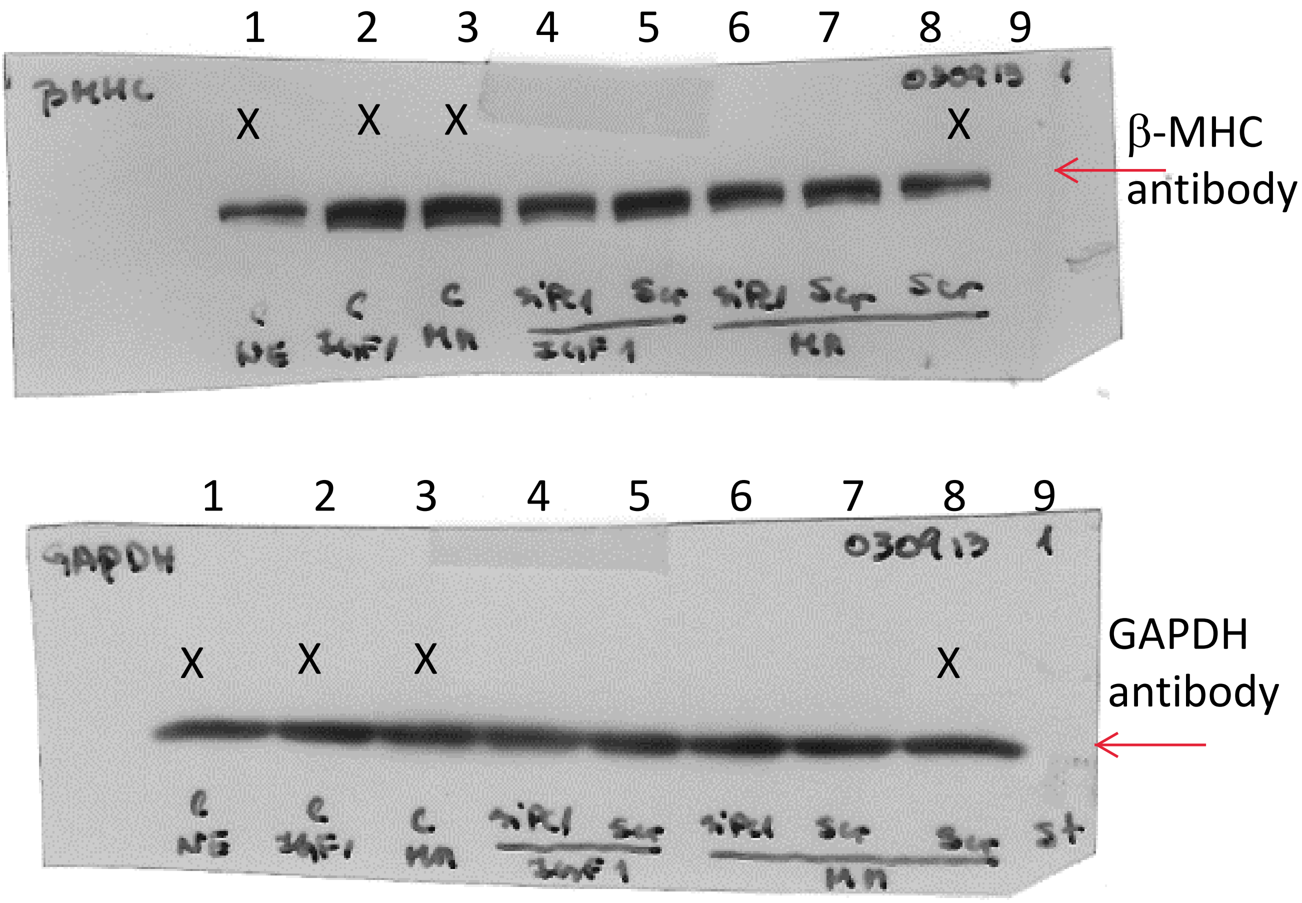

Detection method: EZ-ECL chemiluminescent detection HRP activity, manual development

Fig 1C
n4

Samples:
1: Standard Molecular weight
2. NRVM trated control medium
3. NRVM norepinephrine stimulated
2: NRVM IGF-1 stimulated
3: NRMV trated with UNR siRNA and control medium
4: NRMV trated with PC1 siRNA and control medium
5: NRVM IGF-1 stimulated
6. NRMV trated with UNR siRNA and control medium
7: NRMV trated with PC1 siRNA and IGF-1

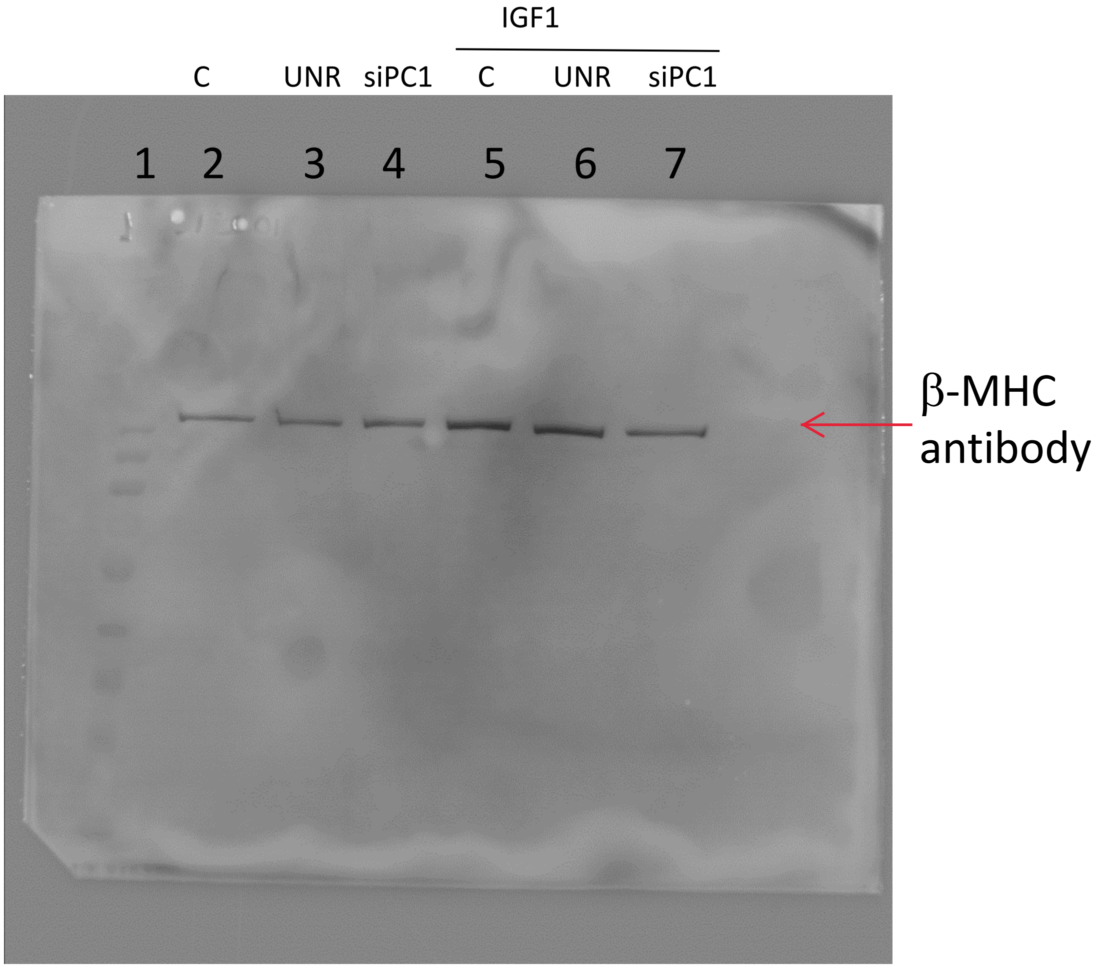

Detection method: EZ-ECL chemiluminescent detection HRP activity, automated

Fig 1C
n4

Samples:
1: Standard Molecular weight
2. NRVM trated control medium
3. NRVM norepinephrine stimulated
2: NRVM IGF-1 stimulated
3: NRMV trated with UNR siRNA and control medium
4: NRMV trated with PC1 siRNA and control medium
5: NRVM IGF-1 stimulated
6. NRMV trated with UNR siRNA and control medium
7: NRMV trated with PC1 siRNA and IGF-1

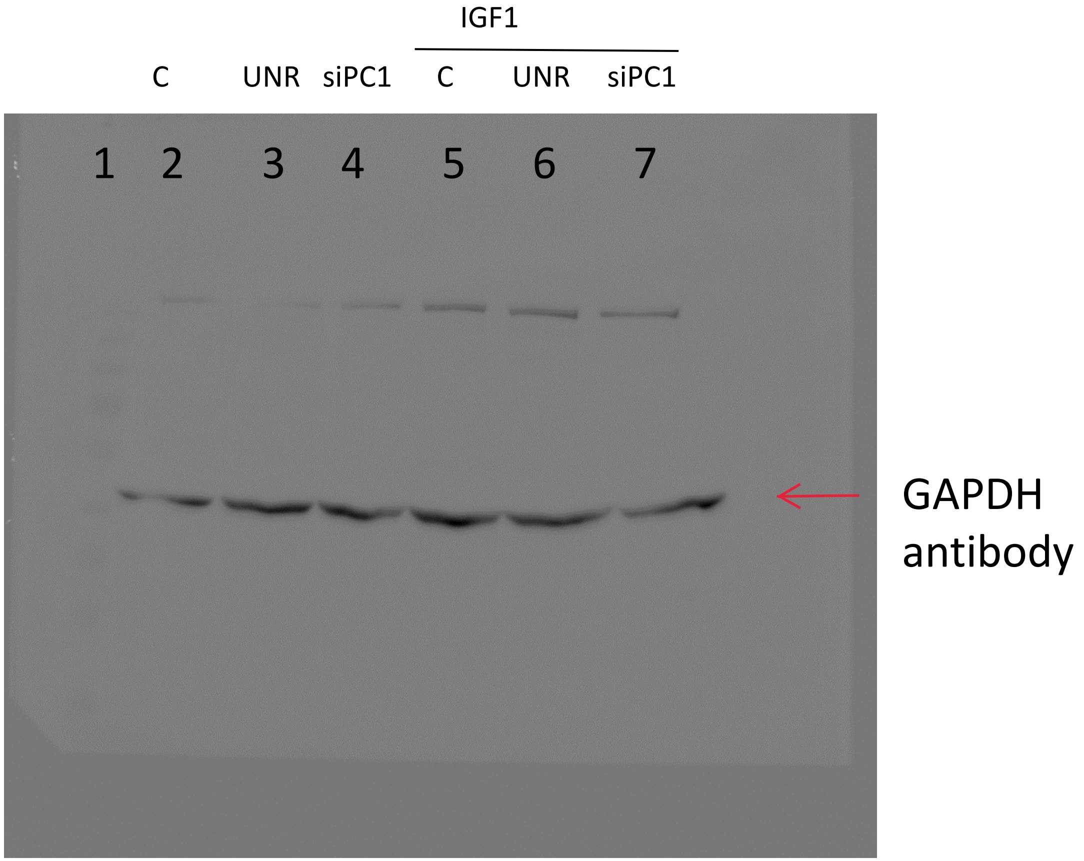

Detection method: EZ-ECL chemiluminescent detection HRP activity, automated

Fig 1C, n5

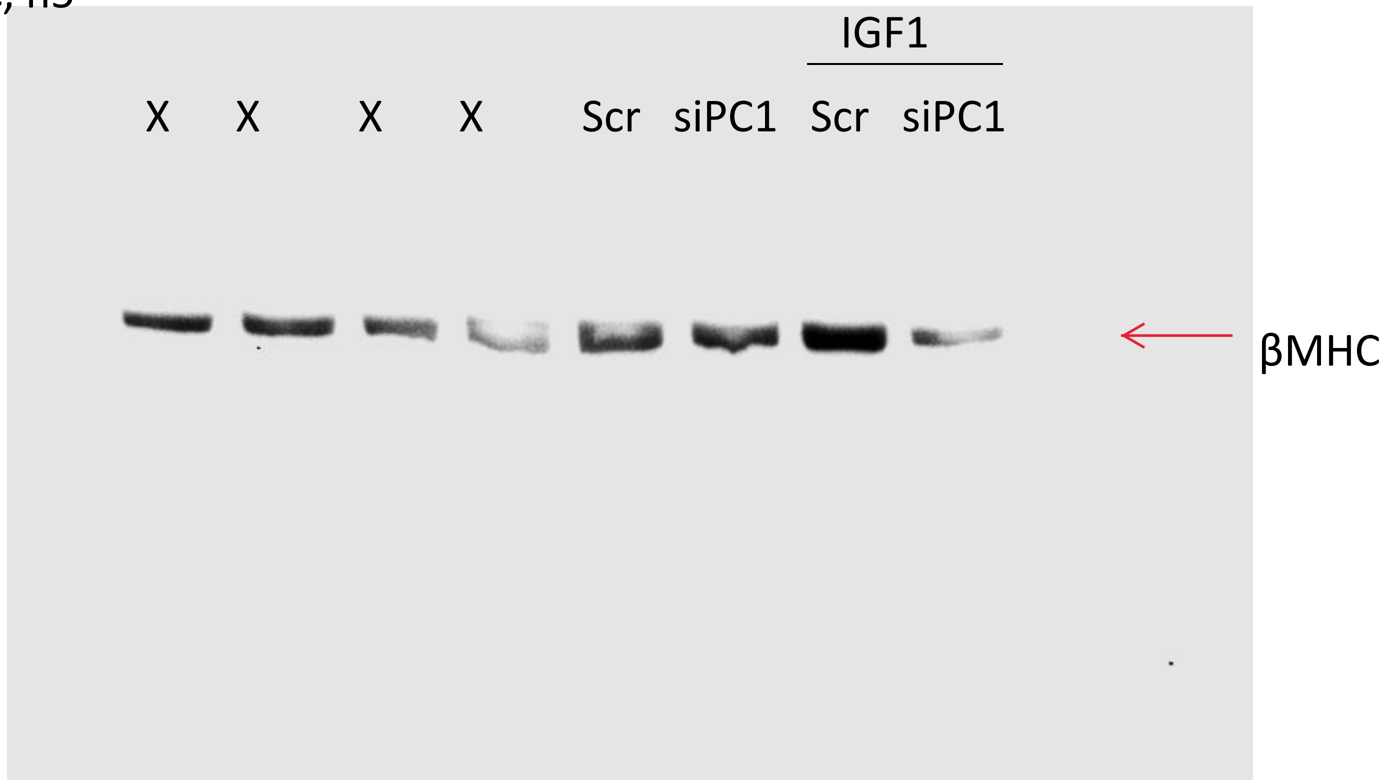
IGF1
X X X X Scr siPC1 Scr siPC1
βMHC

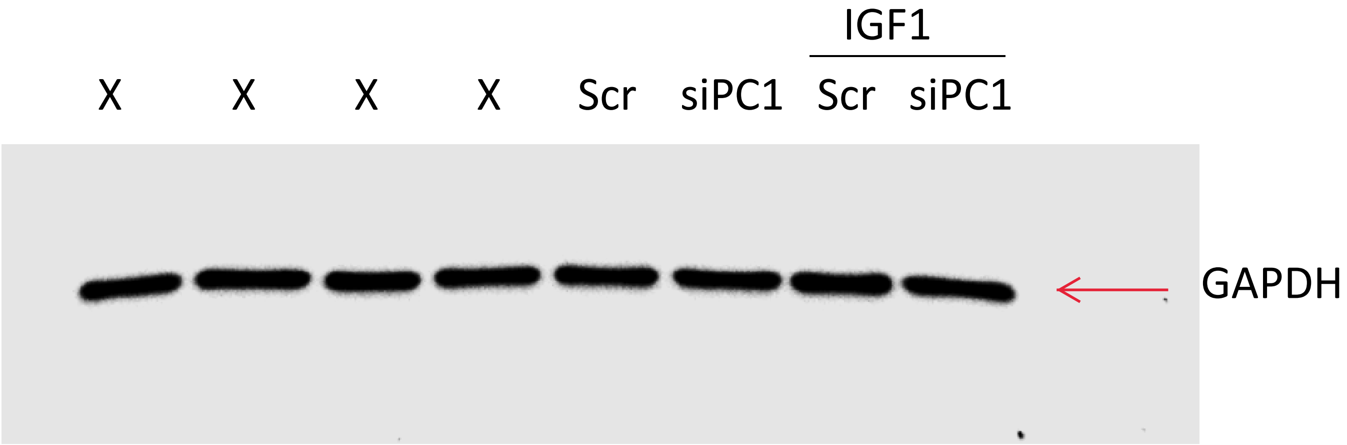
IGF1
X X X X Scr siPC1 Scr siPC1
GAPDH

Fig 3A
n1
Paper figure

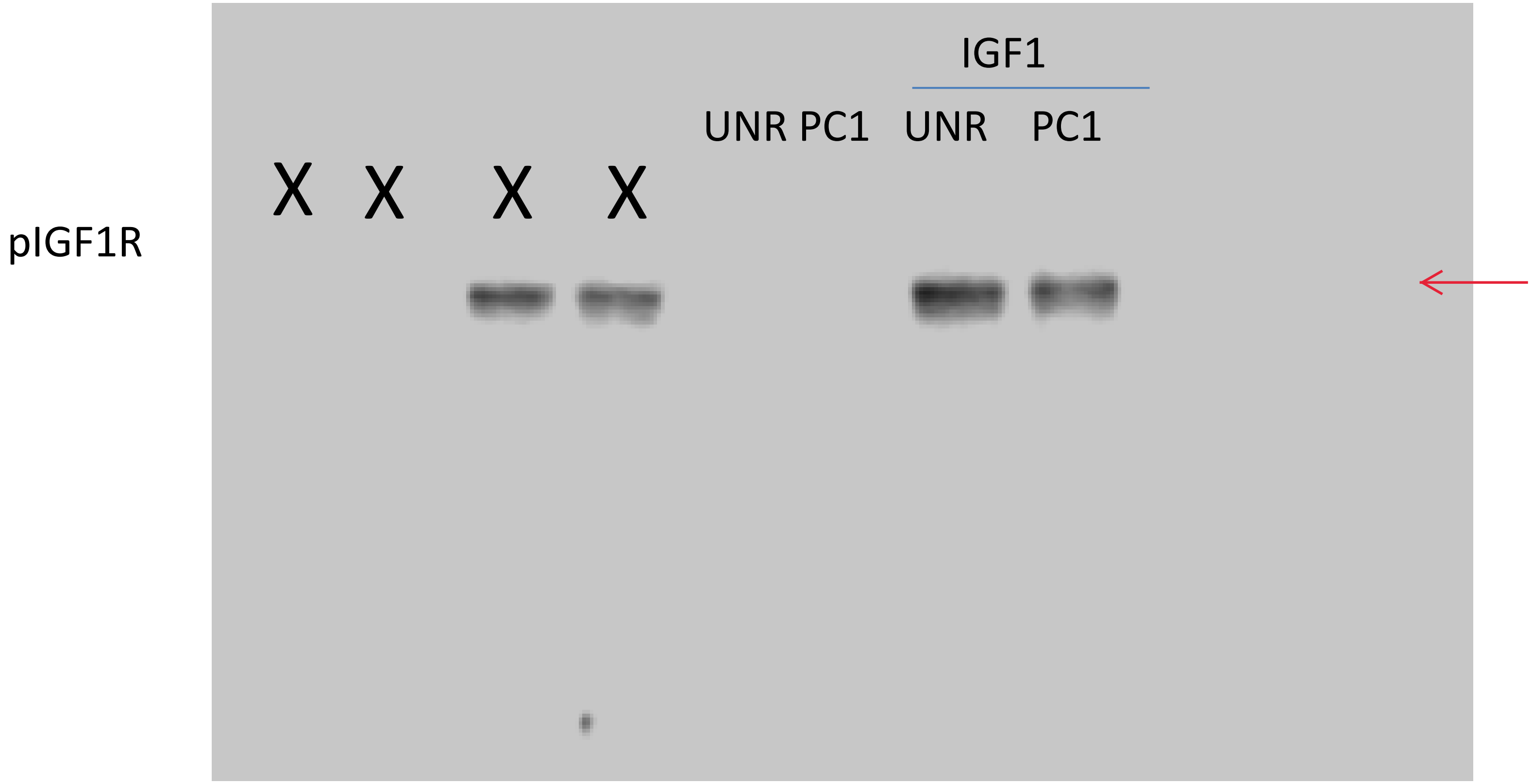

Detection method: EZ-ECL chemiluminescent detection HRP activity, automated

Fig 3A
n1
Paper figure

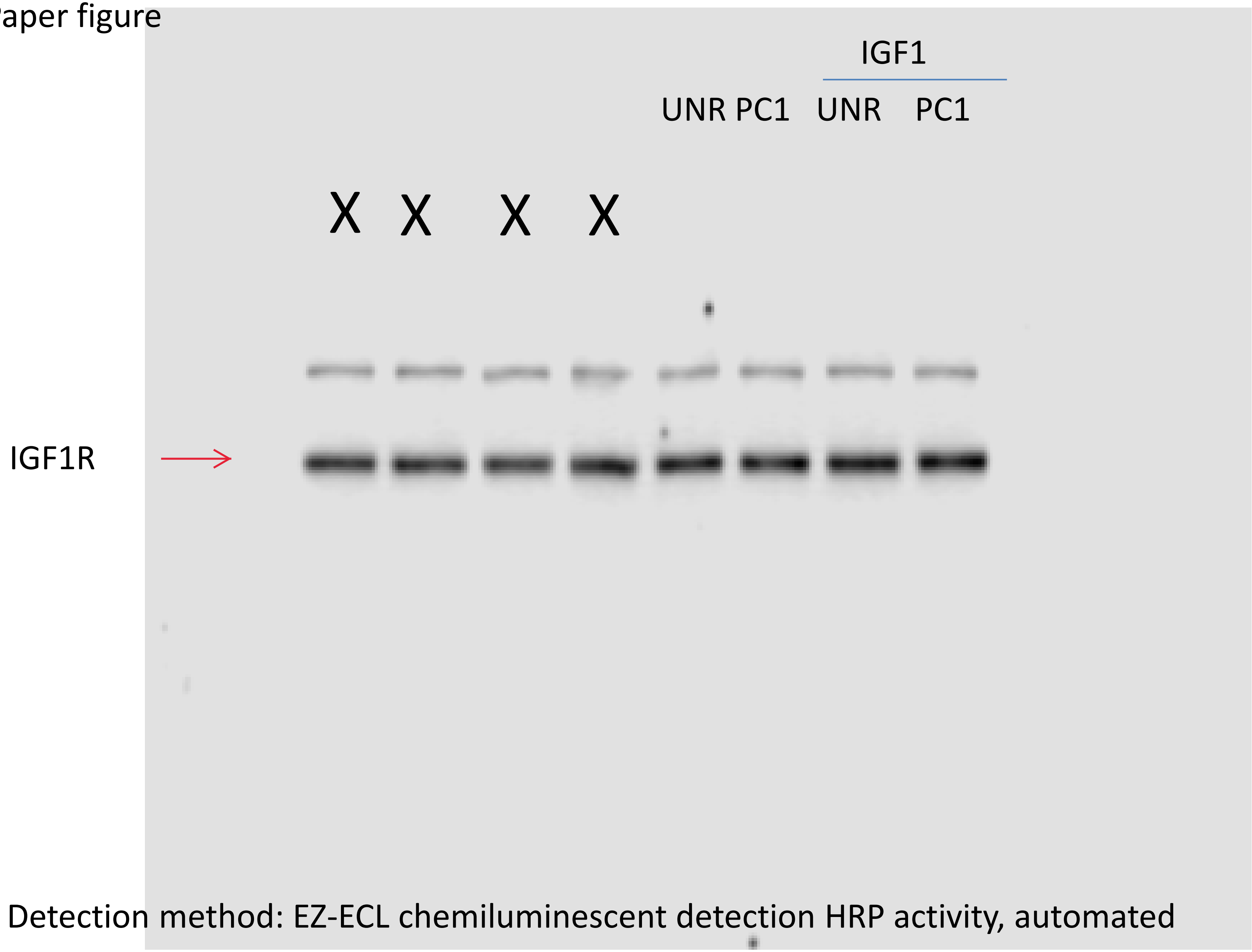

Detection method: EZ-ECL chemiluminescent detection HRP activity, automated

Fig 3A
n1
Paper figure

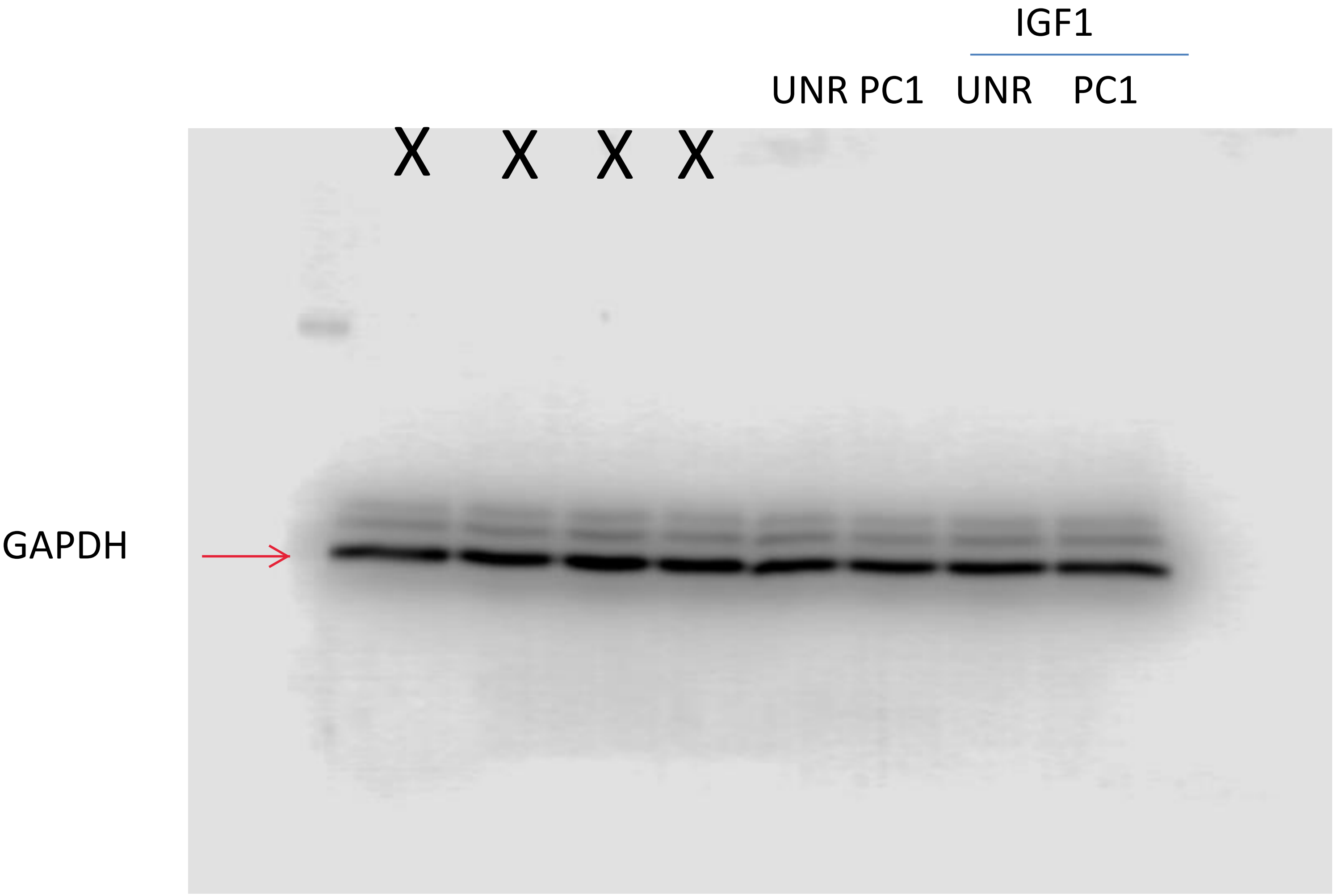

Detection method: EZ-ECL chemiluminescent detection HRP activity, automated

Fig 3A
n2 and n3

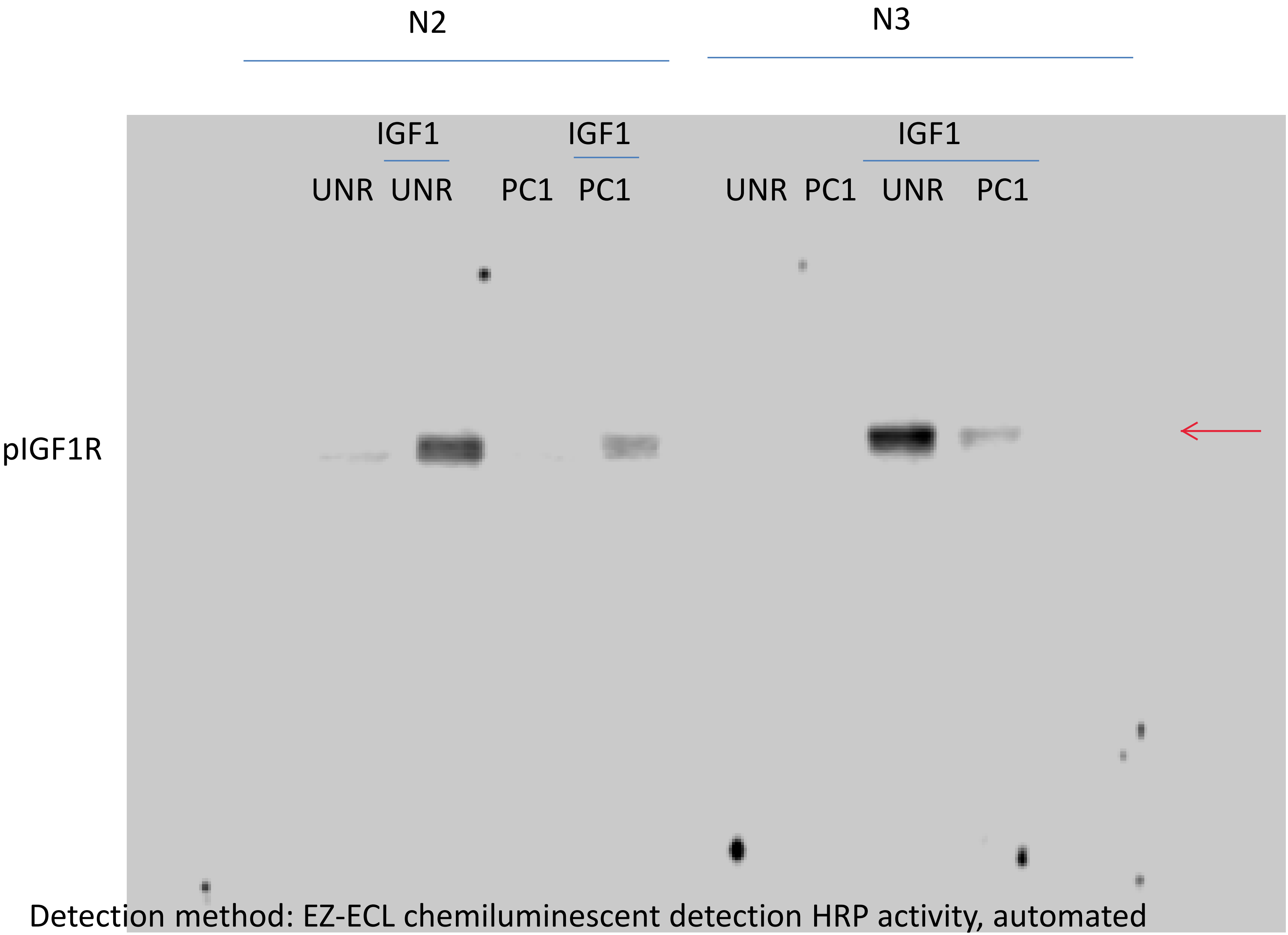

Detection method: EZ-ECL chemiluminescent detection HRP activity, automated

Fig 3A
n2 and n3

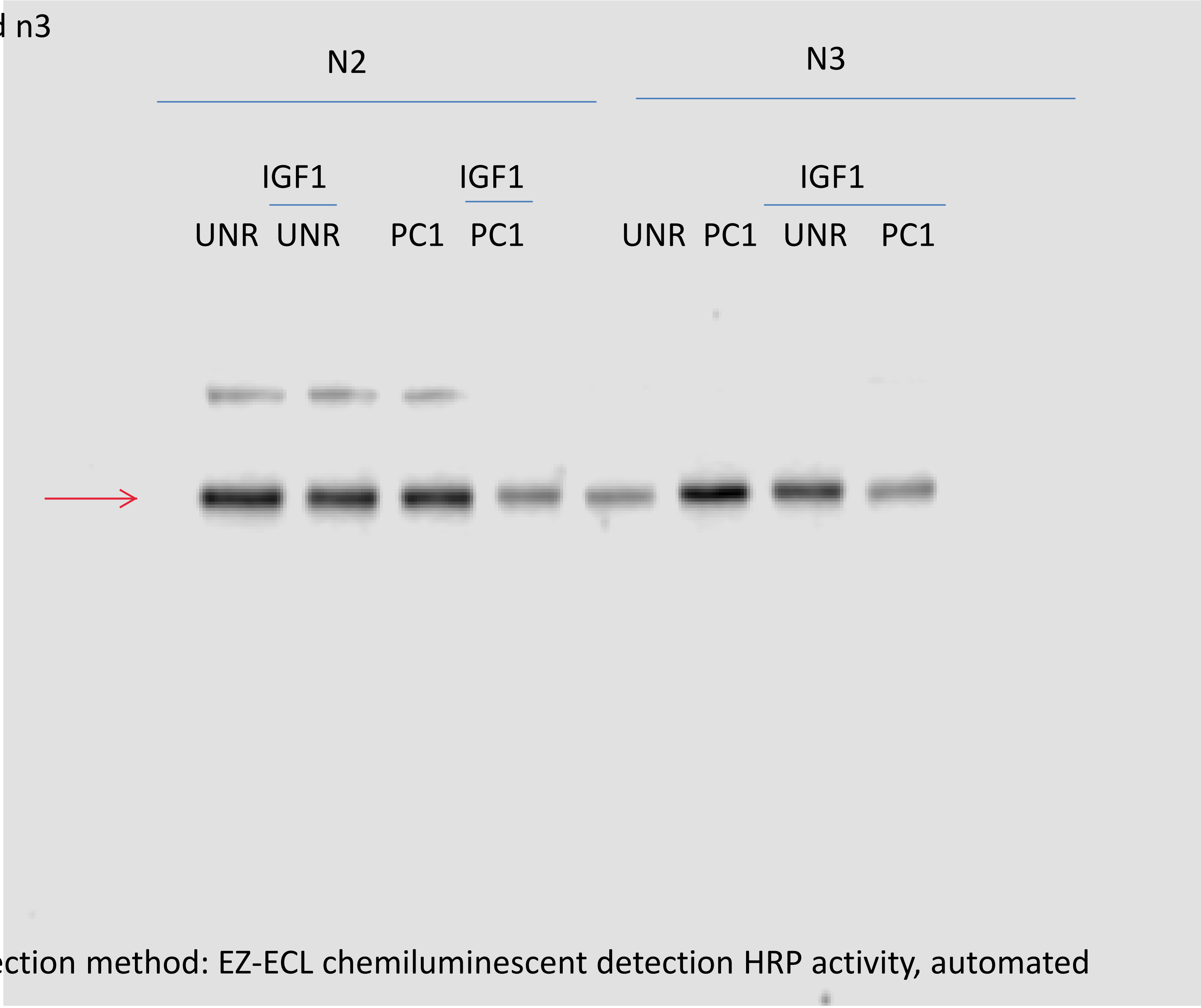

Detection method: EZ-ECL chemiluminescent detection HRP activity, automated

Fig 3A
n2 and n3

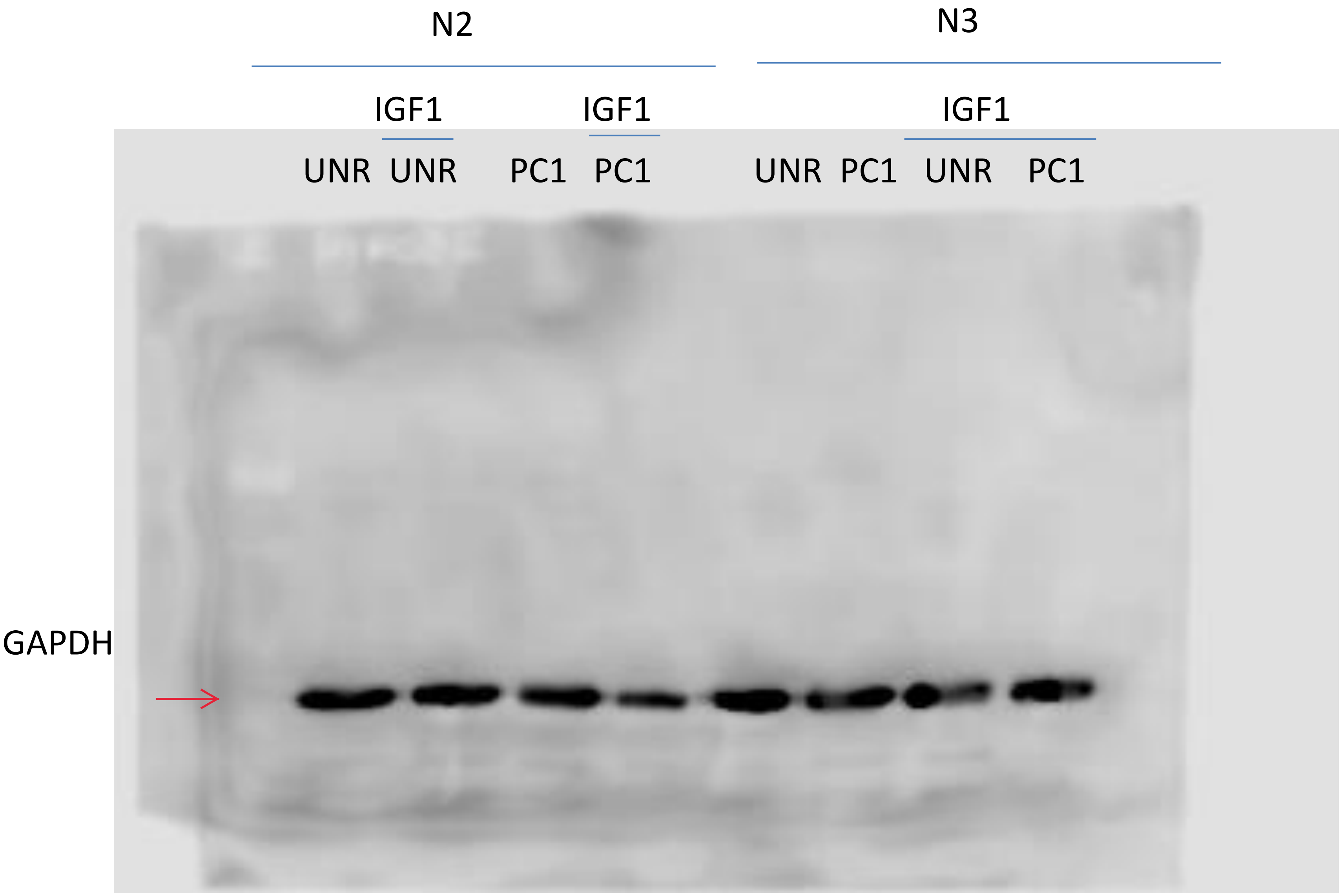

Detection method: EZ-ECL chemiluminescent detection HRP activity, automated

Fig 3A

n4 y n5

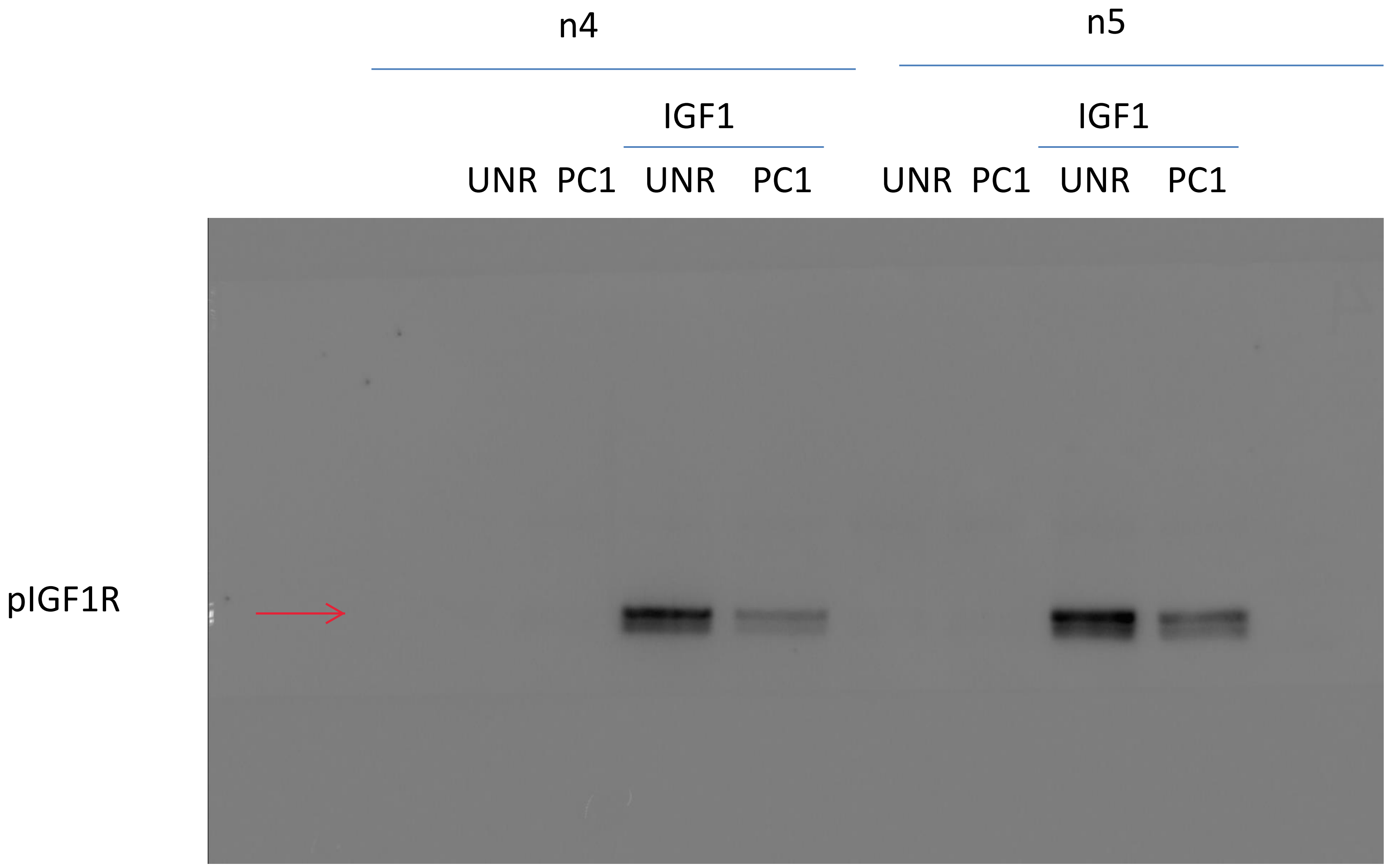

Detection method: EZ-ECL chemiluminescent detection HRP activity, automated

Fig 3A

n4 y n5

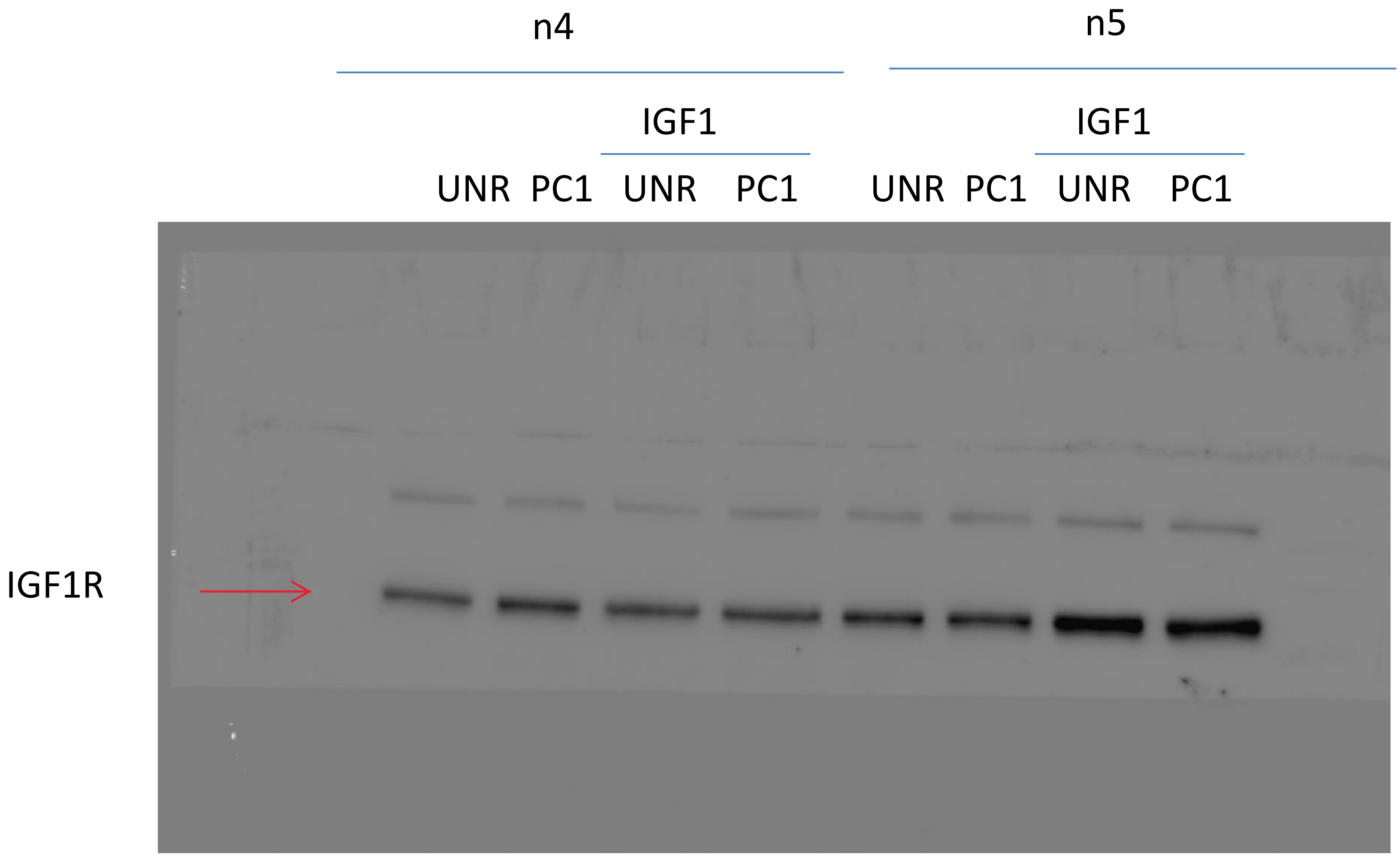

Detection method: EZ-ECL chemiluminescent detection HRP activity, automated

Fig 3A

n4 y n5

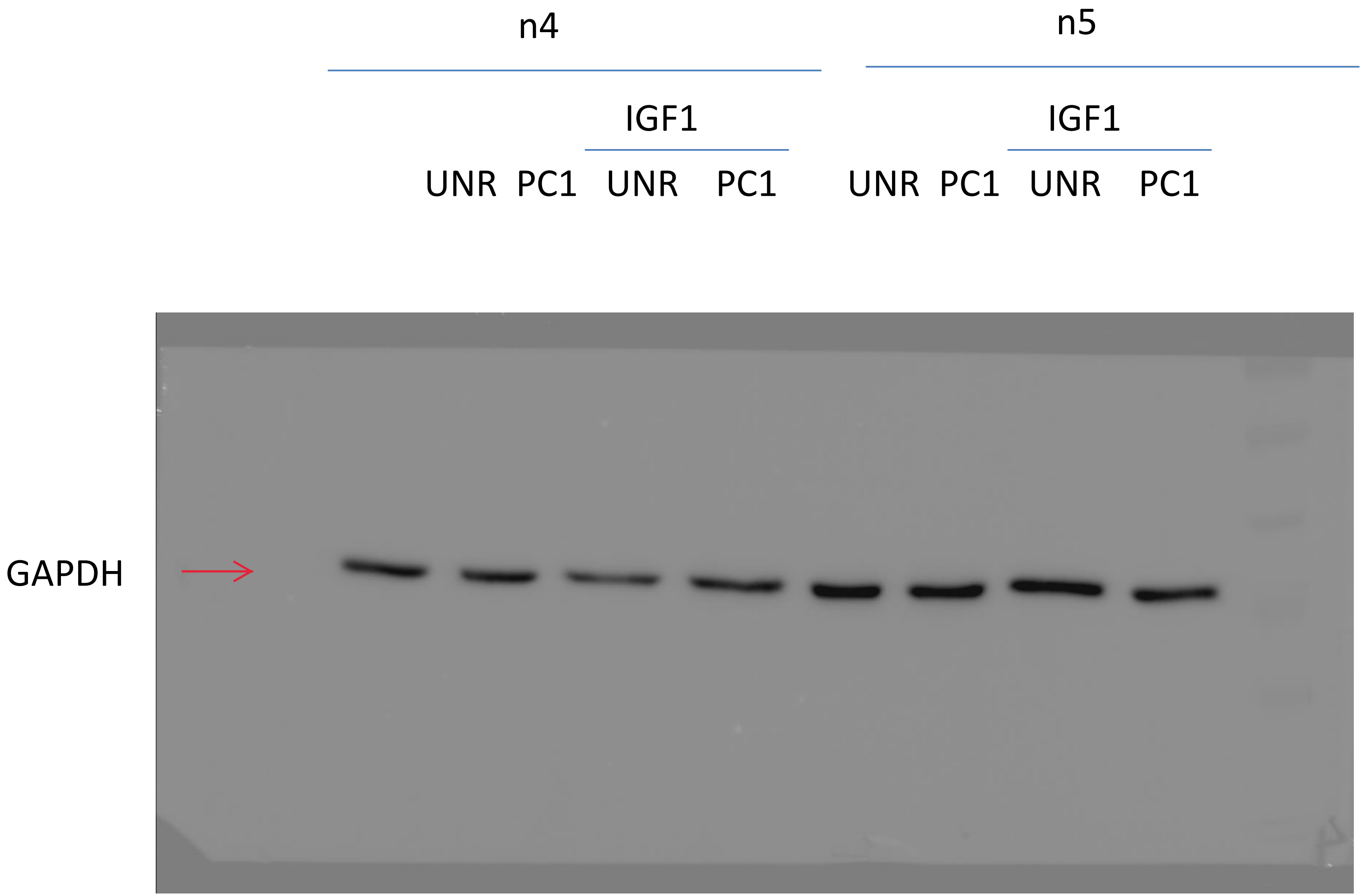

Detection method: EZ-ECL chemiluminescent detection HRP activity, automated

Fig 3B

n1

pAkt

GAPDH

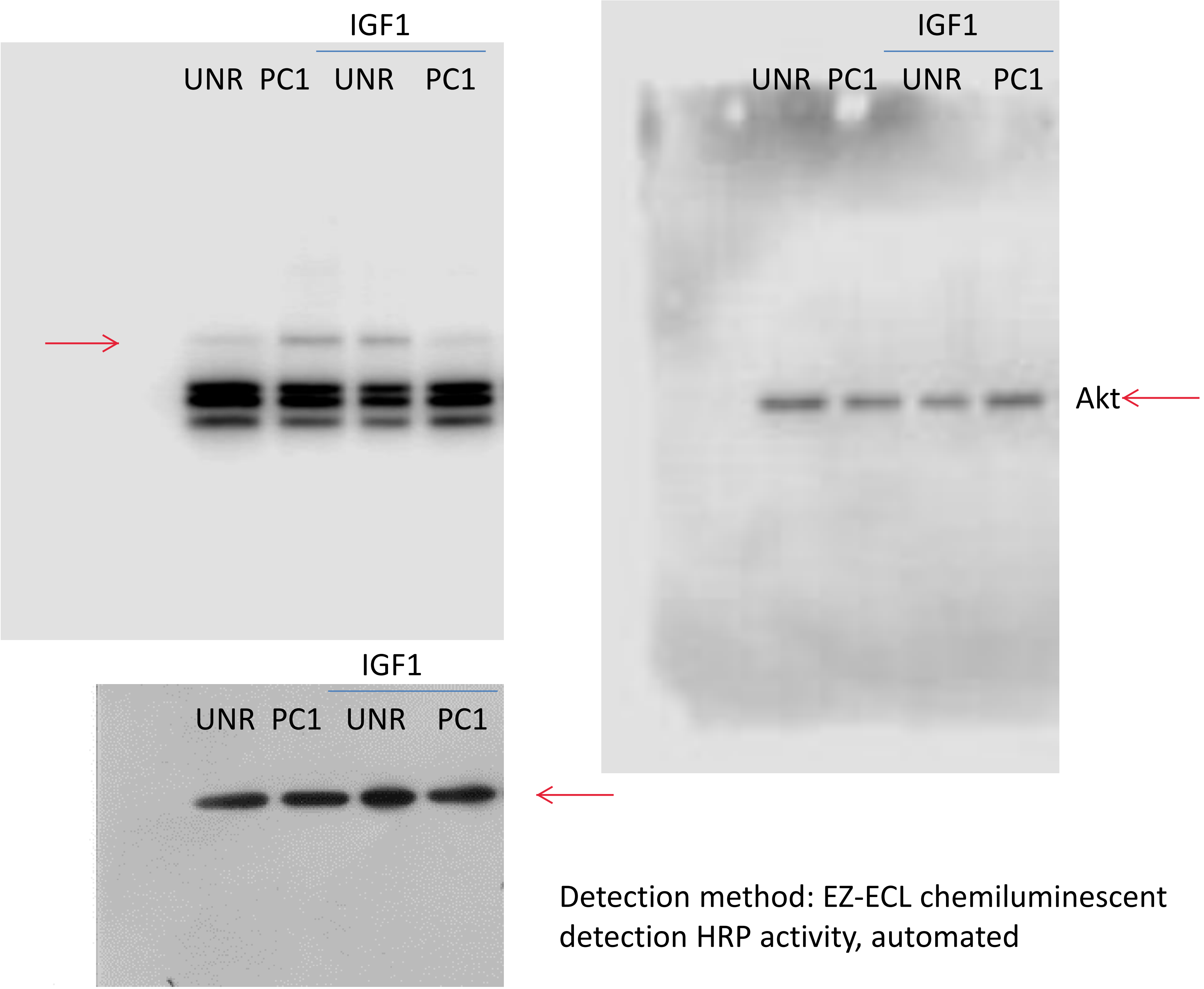

Detection method: EZ-ECL chemiluminescent detection HRP activity, automated

paper figure

Fig 3B, n2

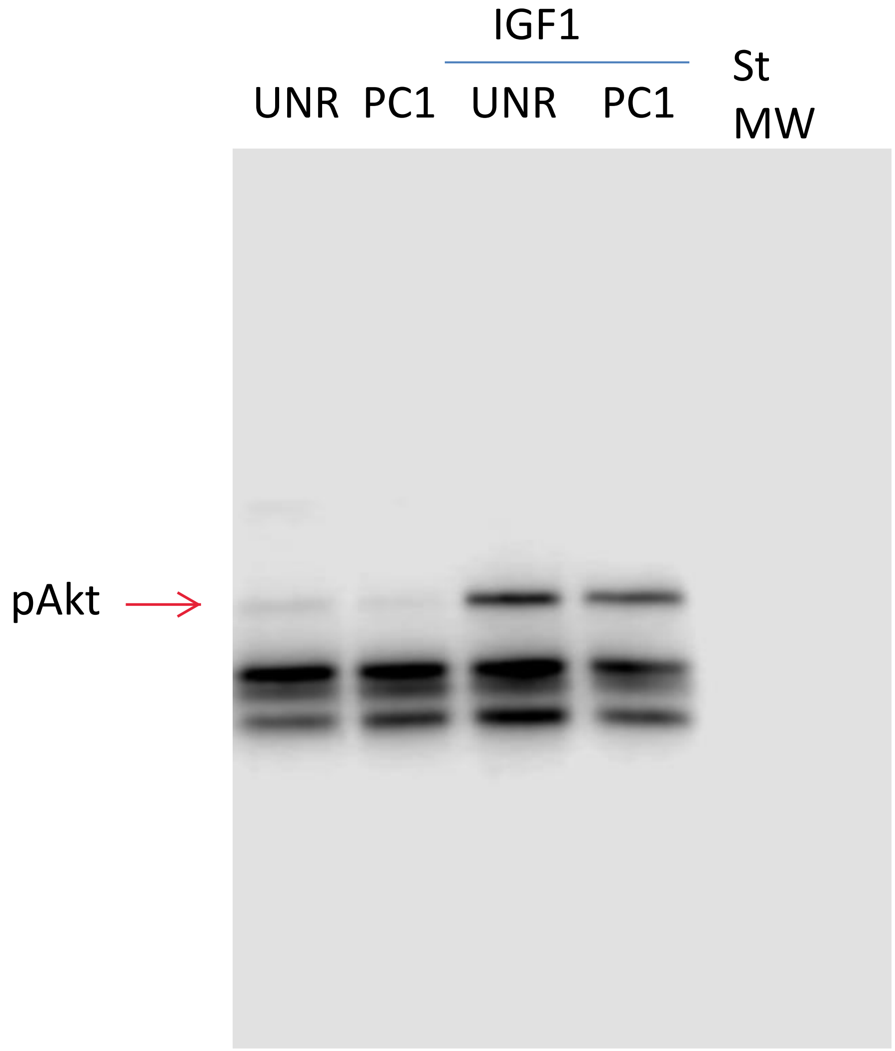

Detection method: EZ-ECL chemiluminescent detection HRP activity, automated

paper figure

Fig 3B, n2

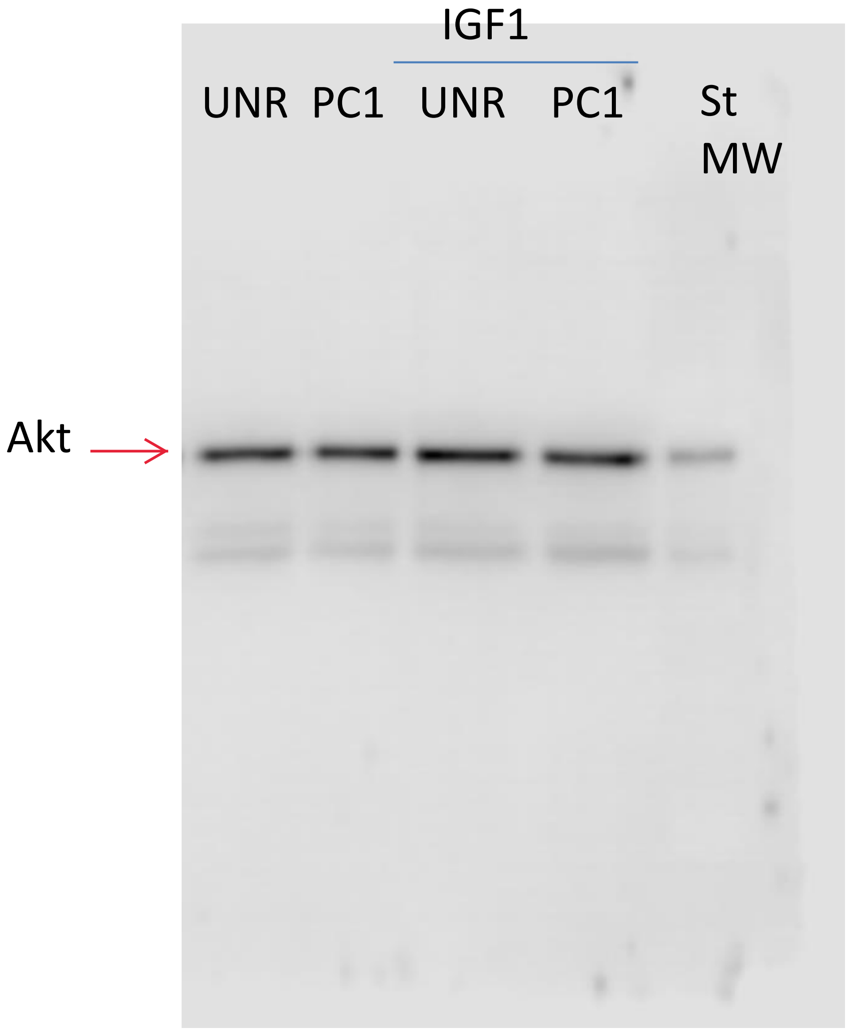

Detection method: EZ-ECL chemiluminescent detection HRP activity, automated

paper figure

Fig 3B, n2

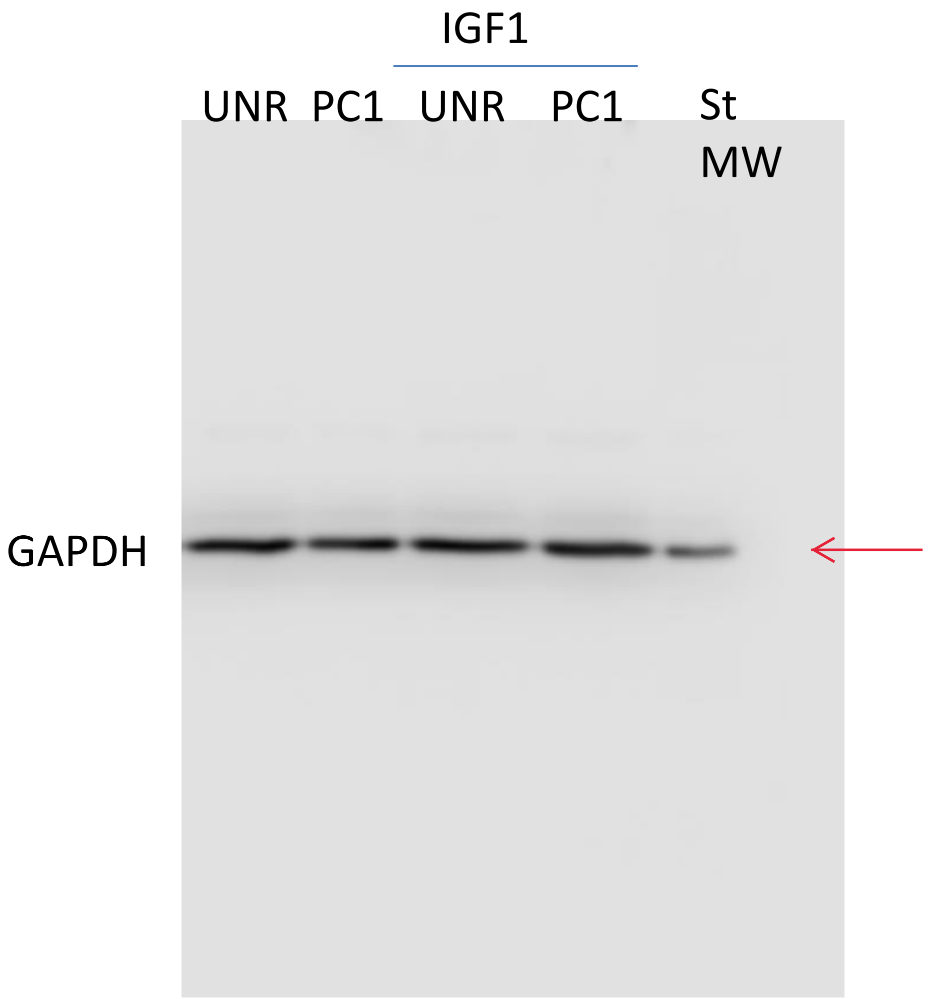

Detection method: EZ-ECL chemiluminescent detection HRP activity, automated

Fig 3B

n 3 y n 4

n3 n4

IGF1 IGF1

UNR PC1 UNR PC1 UNR PC1 UNR PC1

pAkt

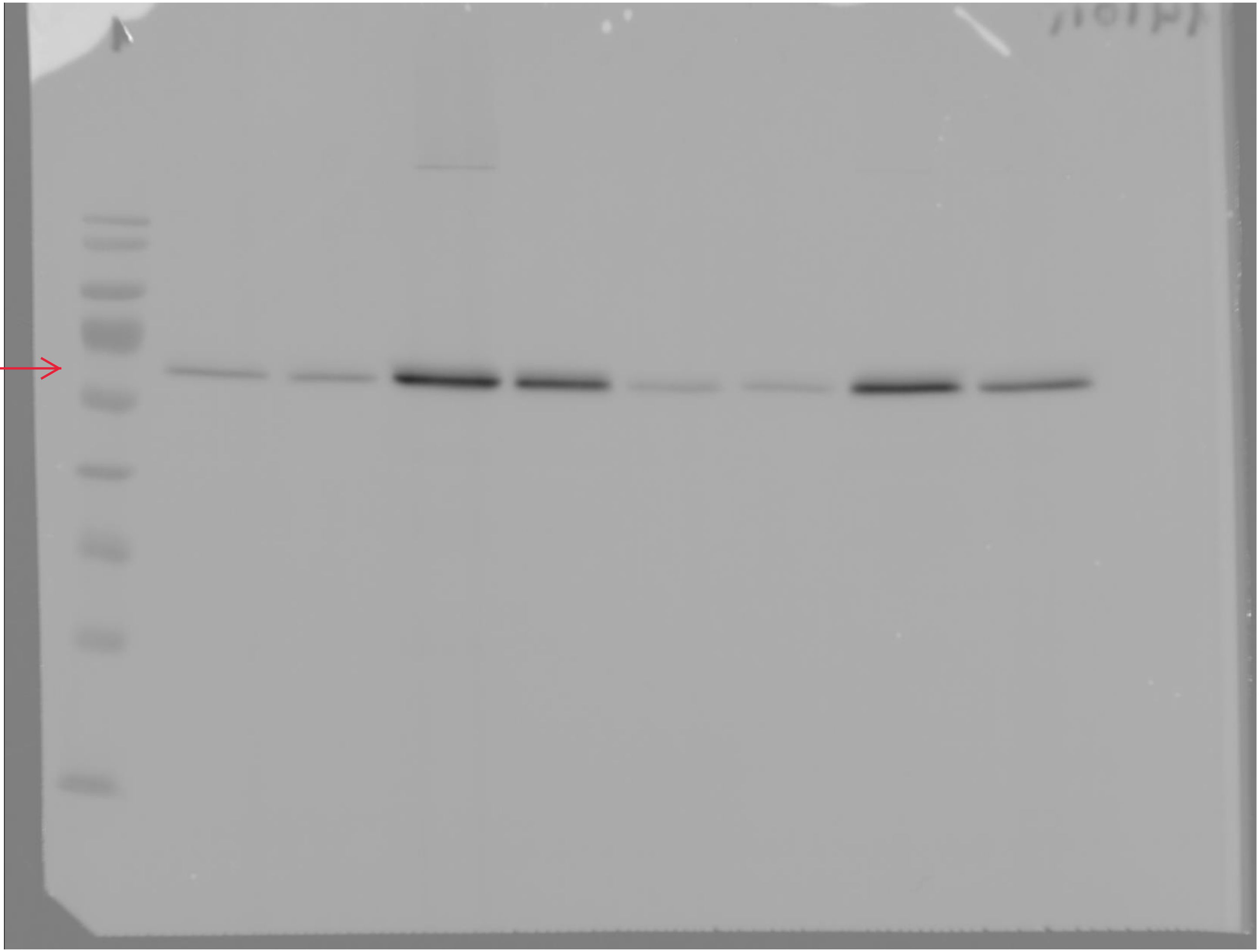

Detection method: EZ-ECL chemiluminescent detection HRP activity, automated

Fig 3B

n 3 y n 4

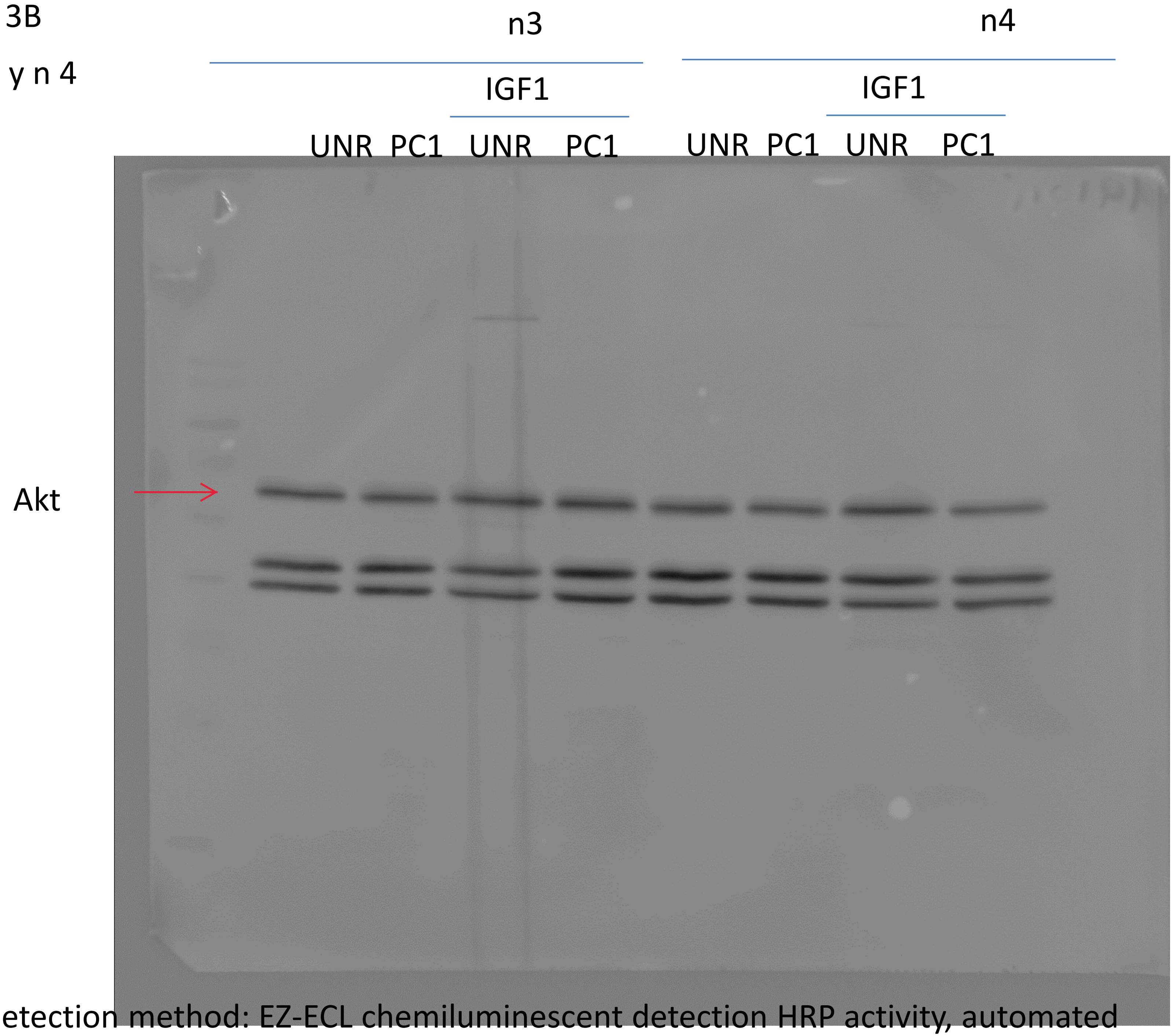

Detection method: EZ-ECL chemiluminescent detection HRP activity, automated

Fig 3B

n 3 y n 4

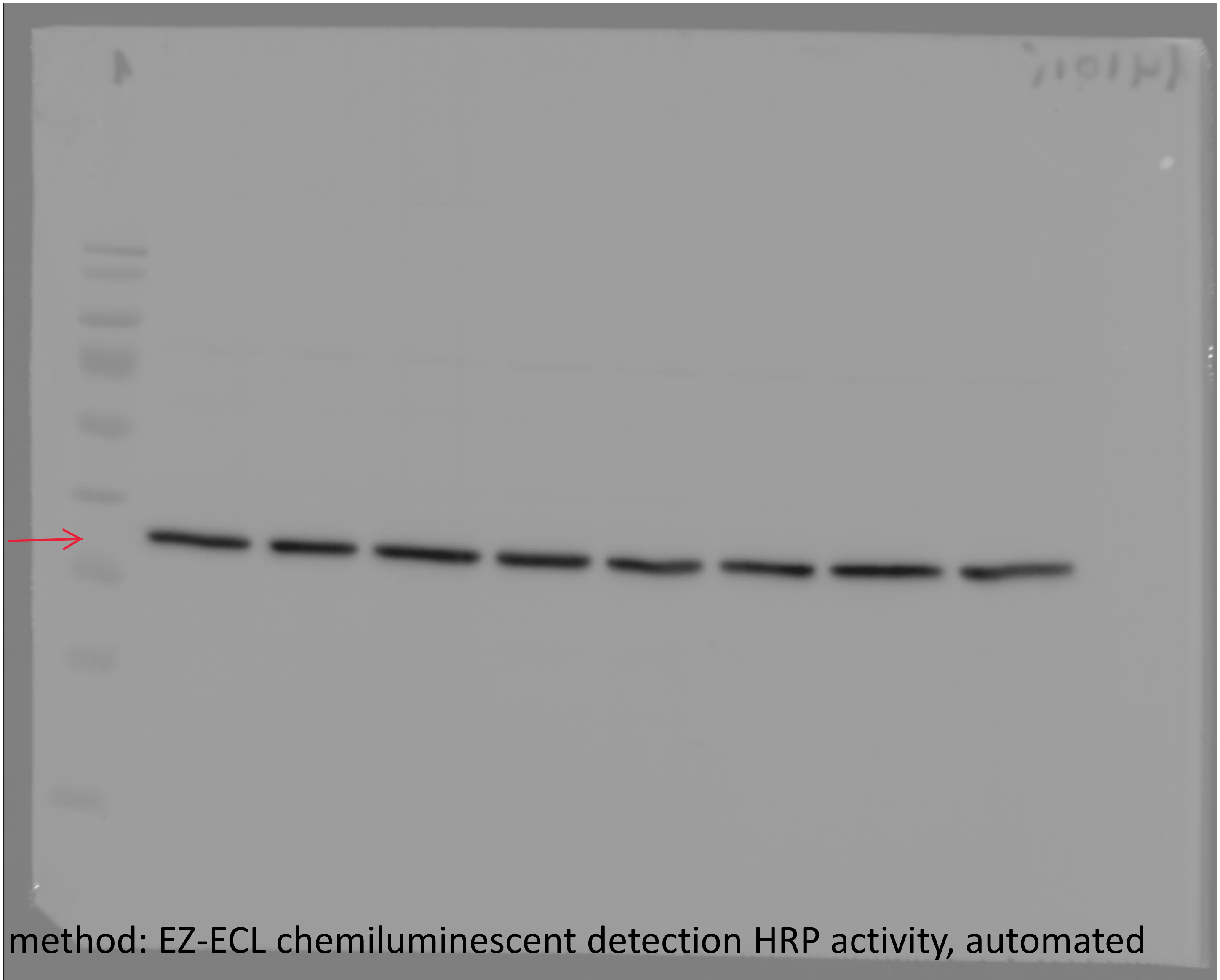

Detection method: EZ-ECL chemiluminescent detection HRP activity, automated

Fig 3B

n 5

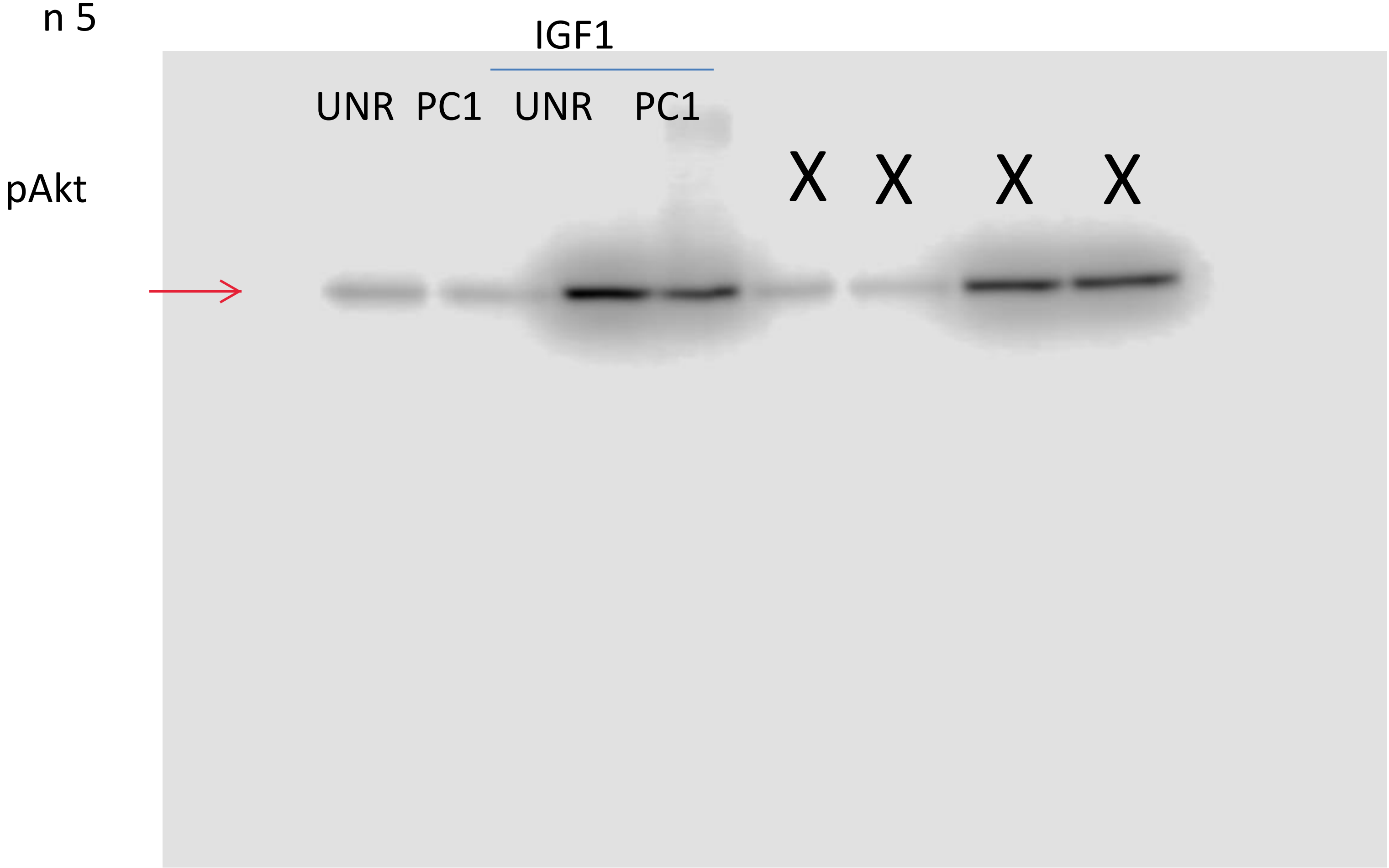

Detection method: EZ-ECL chemiluminescent detection HRP activity, automated

Fig 3B
n 5

GAPDH membrane pAKT

IGF1

UNR PC1 UNR PC1

GAPDH

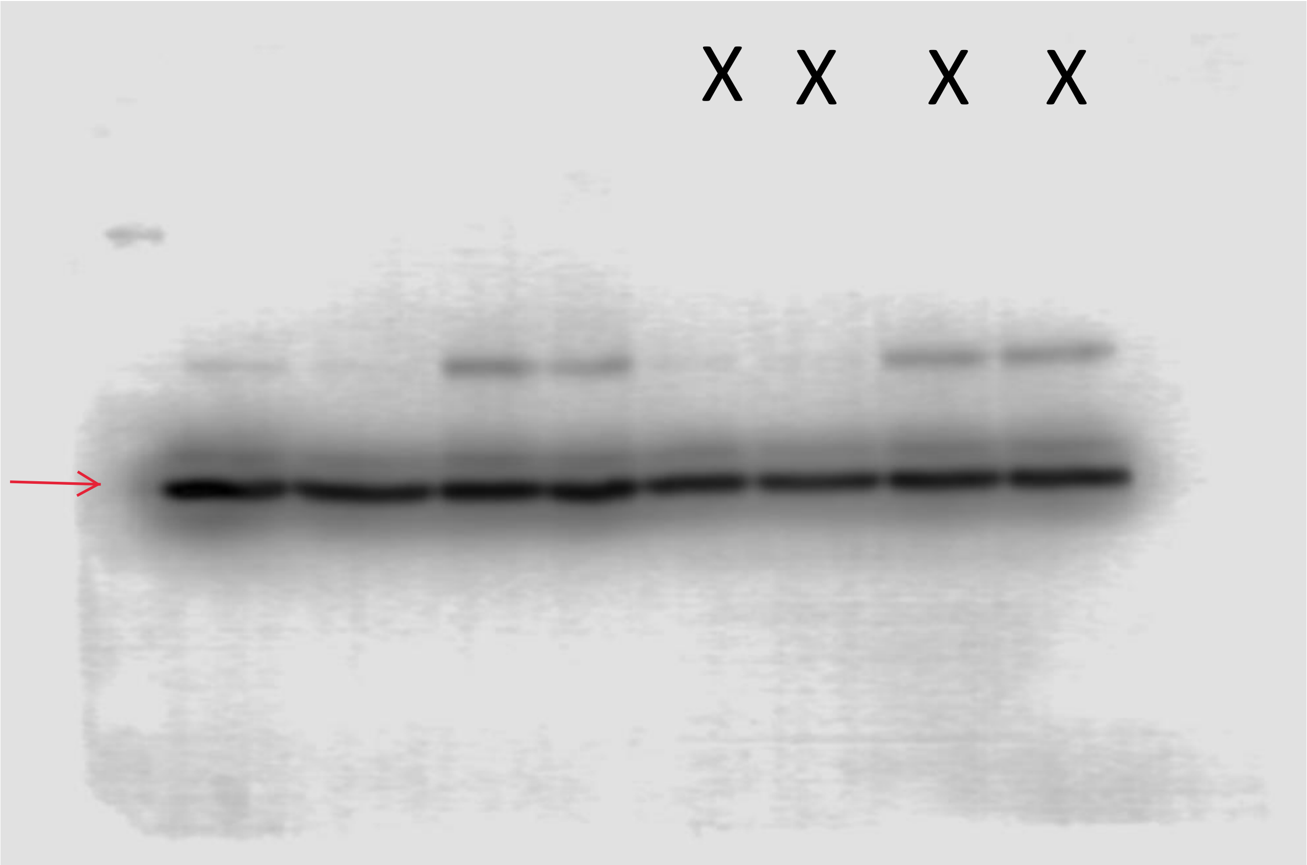

Detection method: EZ-ECL chemiluminescent detection HRP activity, automated

Fig 3B n 5

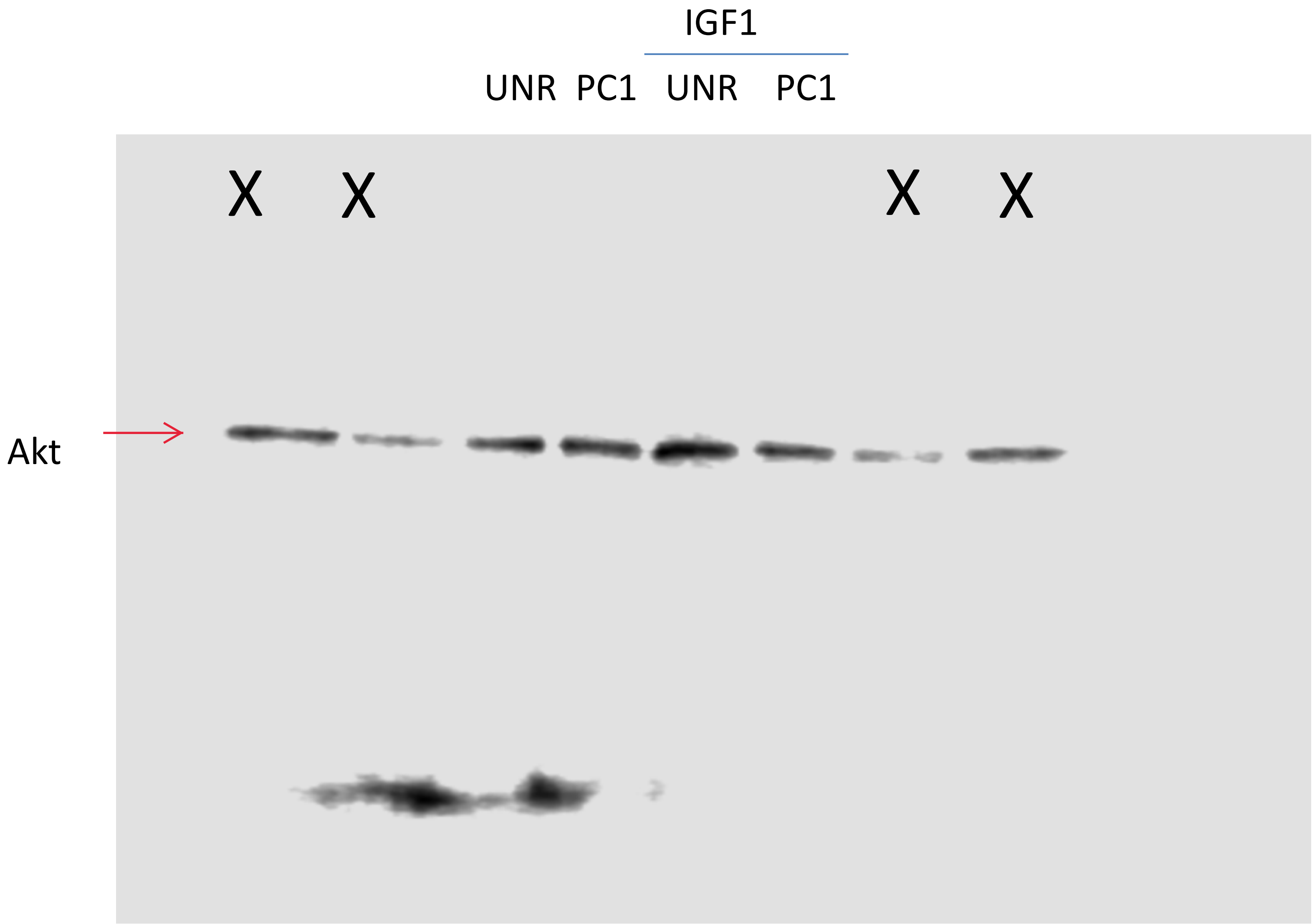

Detection method: EZ-ECL chemiluminescent detection HRP activity, automated

Fig 3B
n5
GAPDH membrane AKT

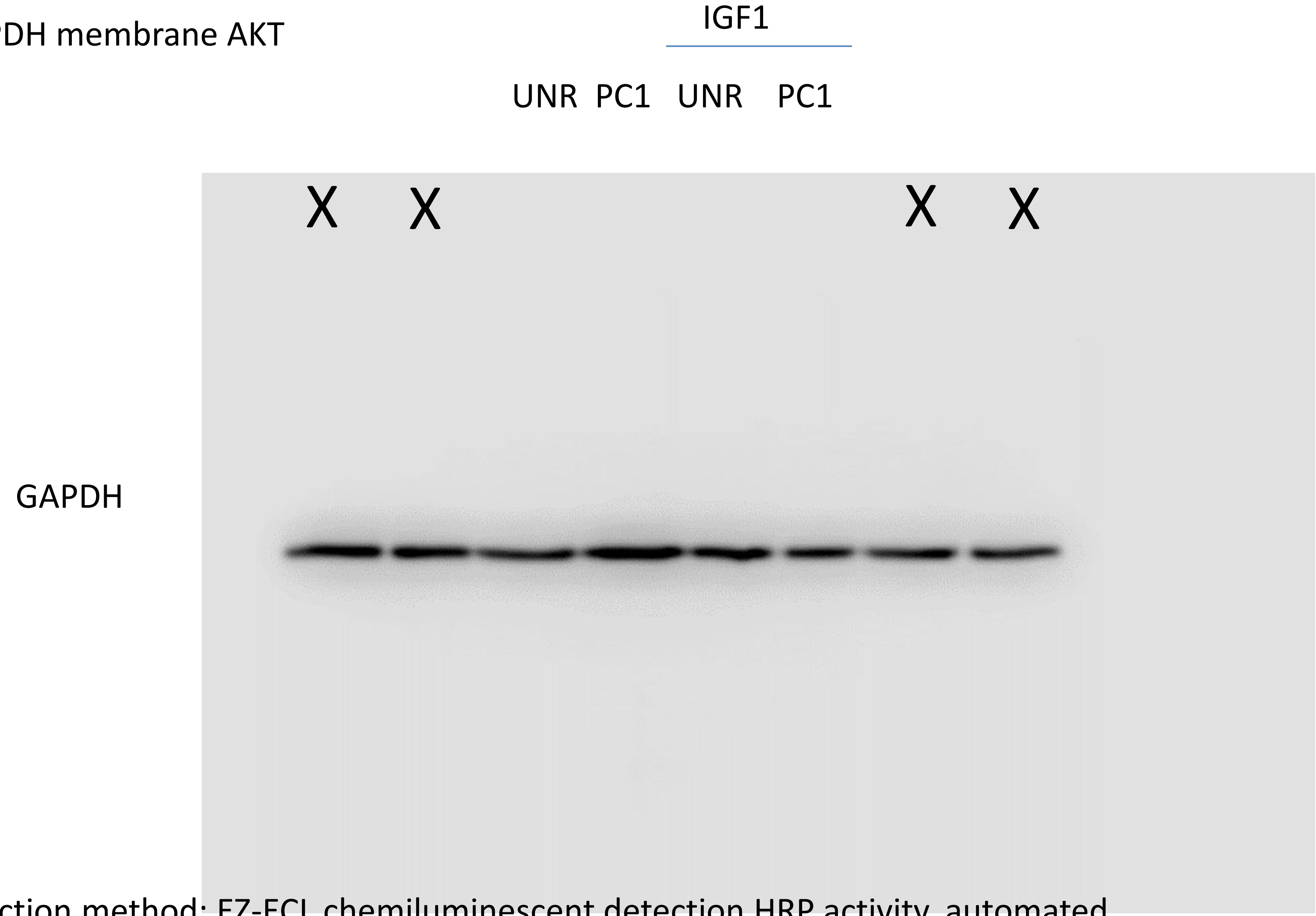

Detection method: EZ-ECL chemiluminescent detection HRP activity, automated

Fig 3C pERK

n1

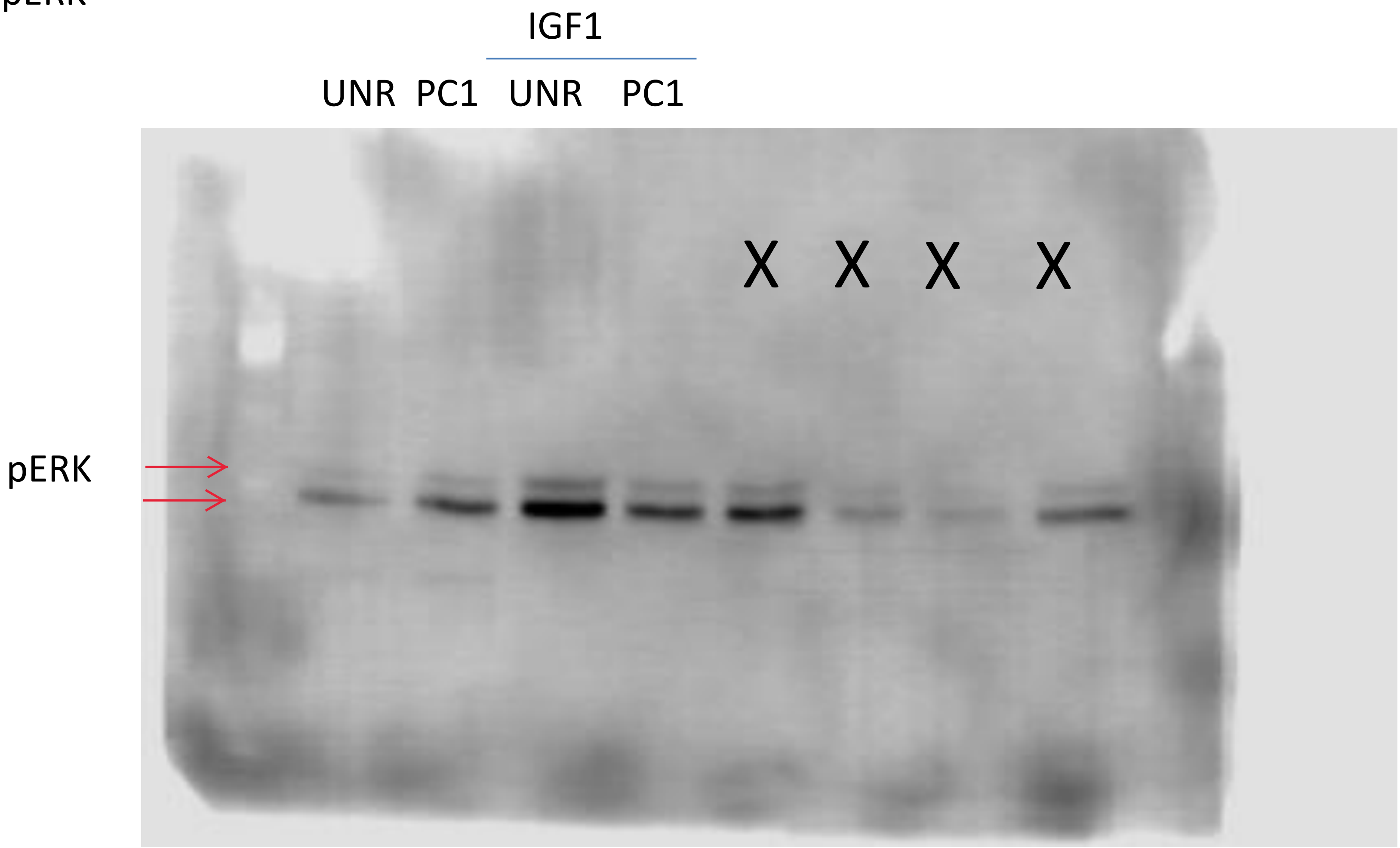

Detection method: EZ-ECL chemiluminescent detection HRP activity, automated

Fig 3C pERK

n1

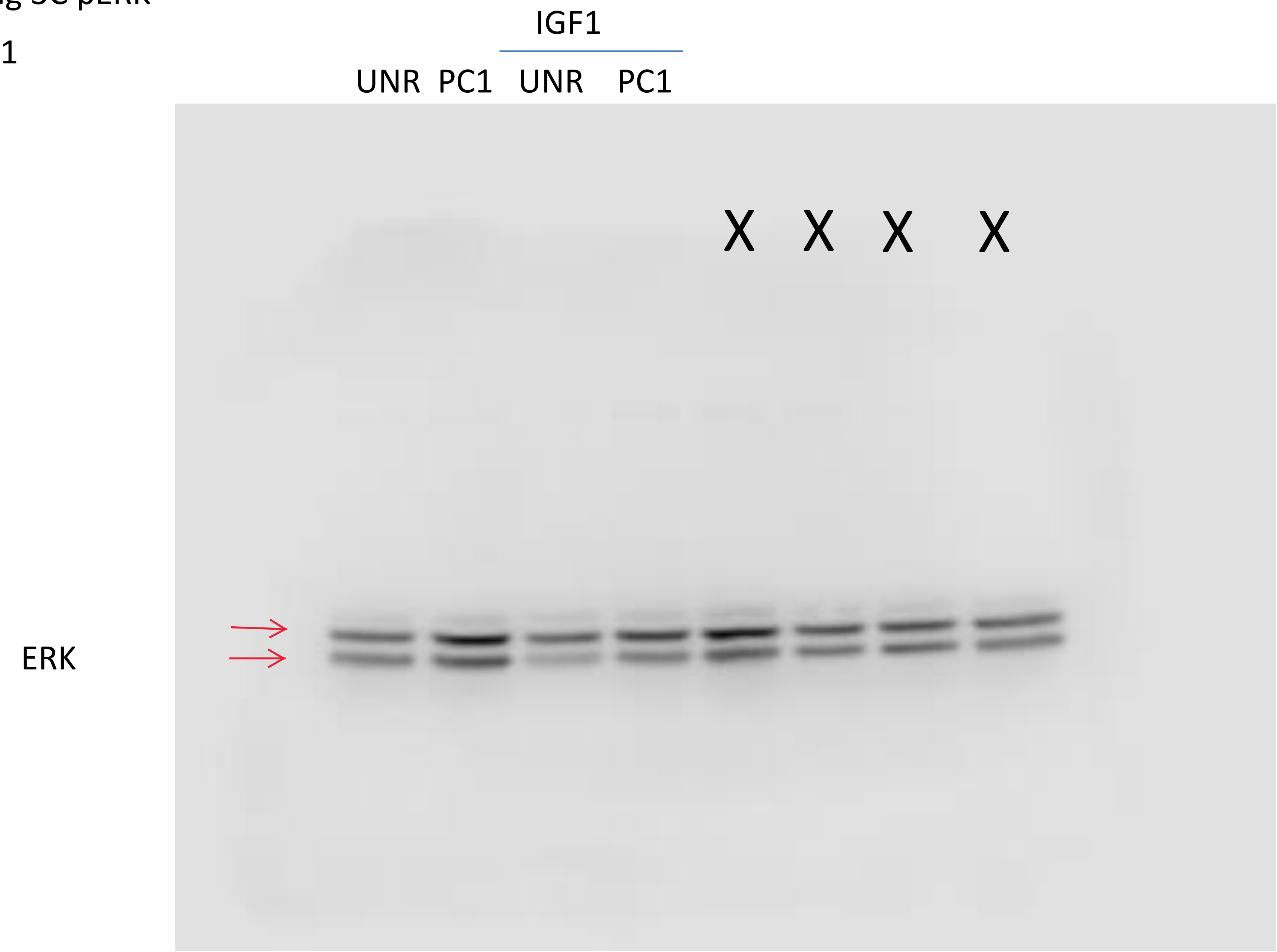

Detection method: EZ-ECL chemiluminescent detection HRP activity, automated

Fig 3C pERK

n1

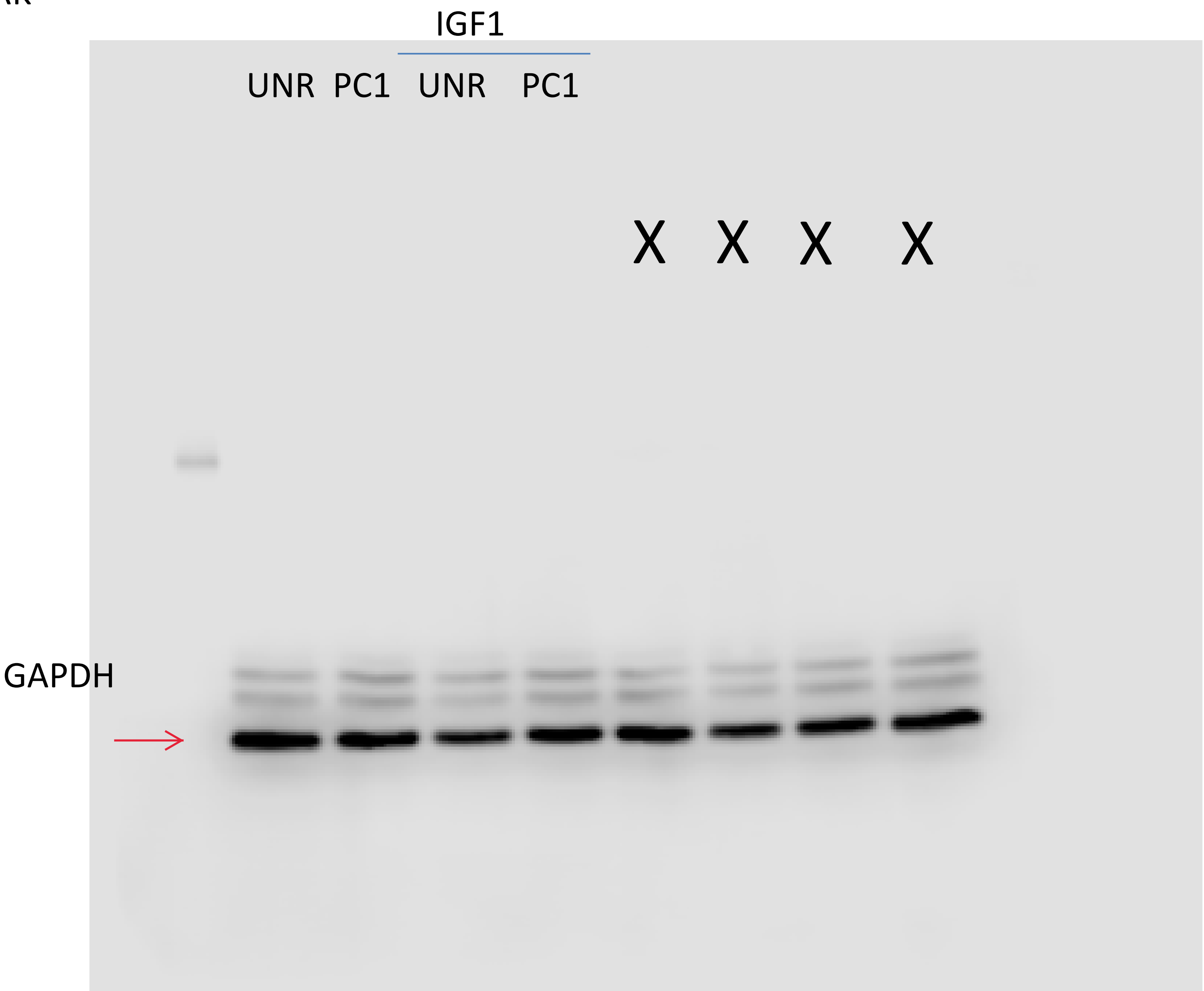

Fig 3C

n2

Paper figure

IGF1

UNR PC1 UNR PC1

pERK

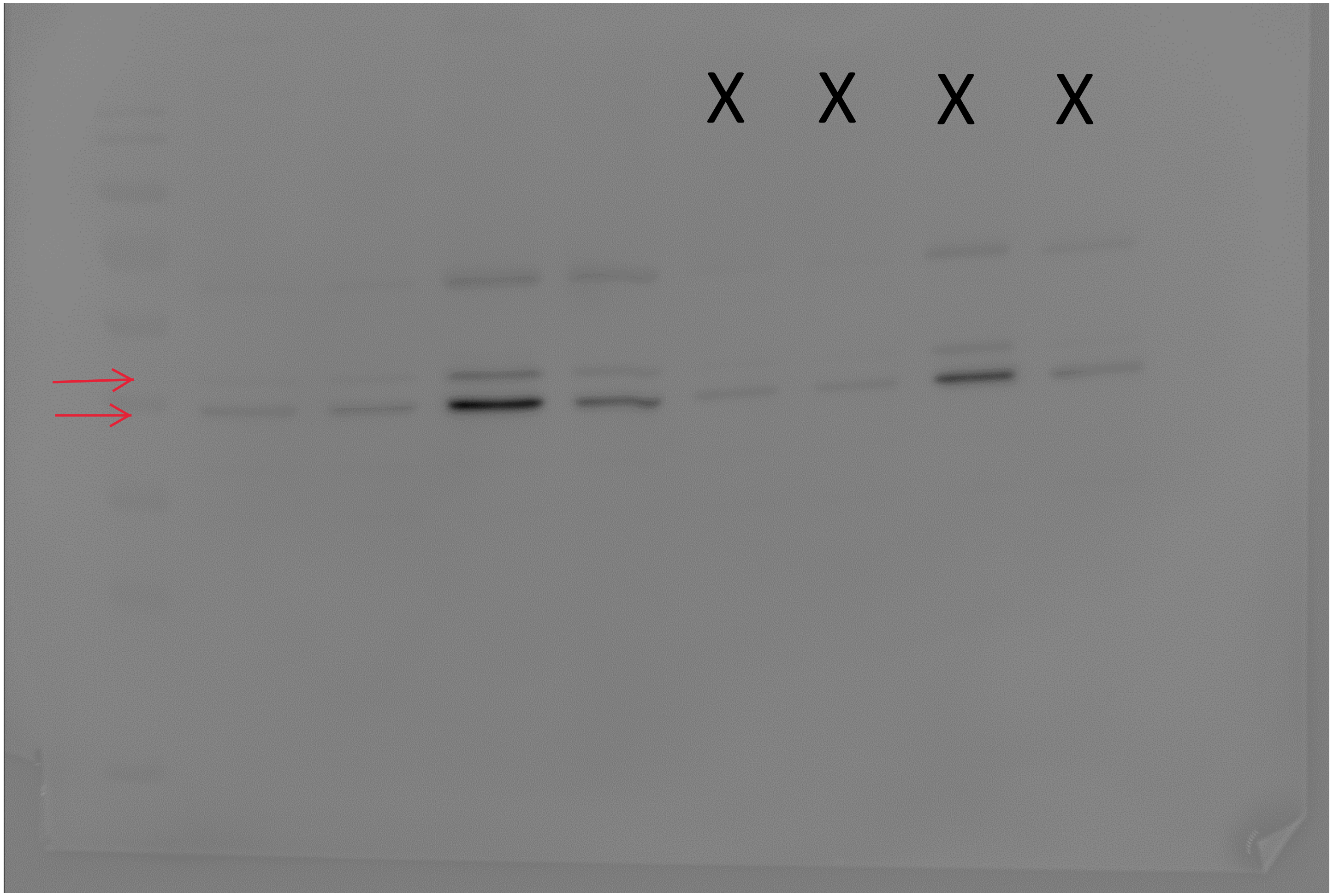

Detection method: EZ-ECL chemiluminescent detection HRP activity, automated

Fig 3C

n2

Paper figure

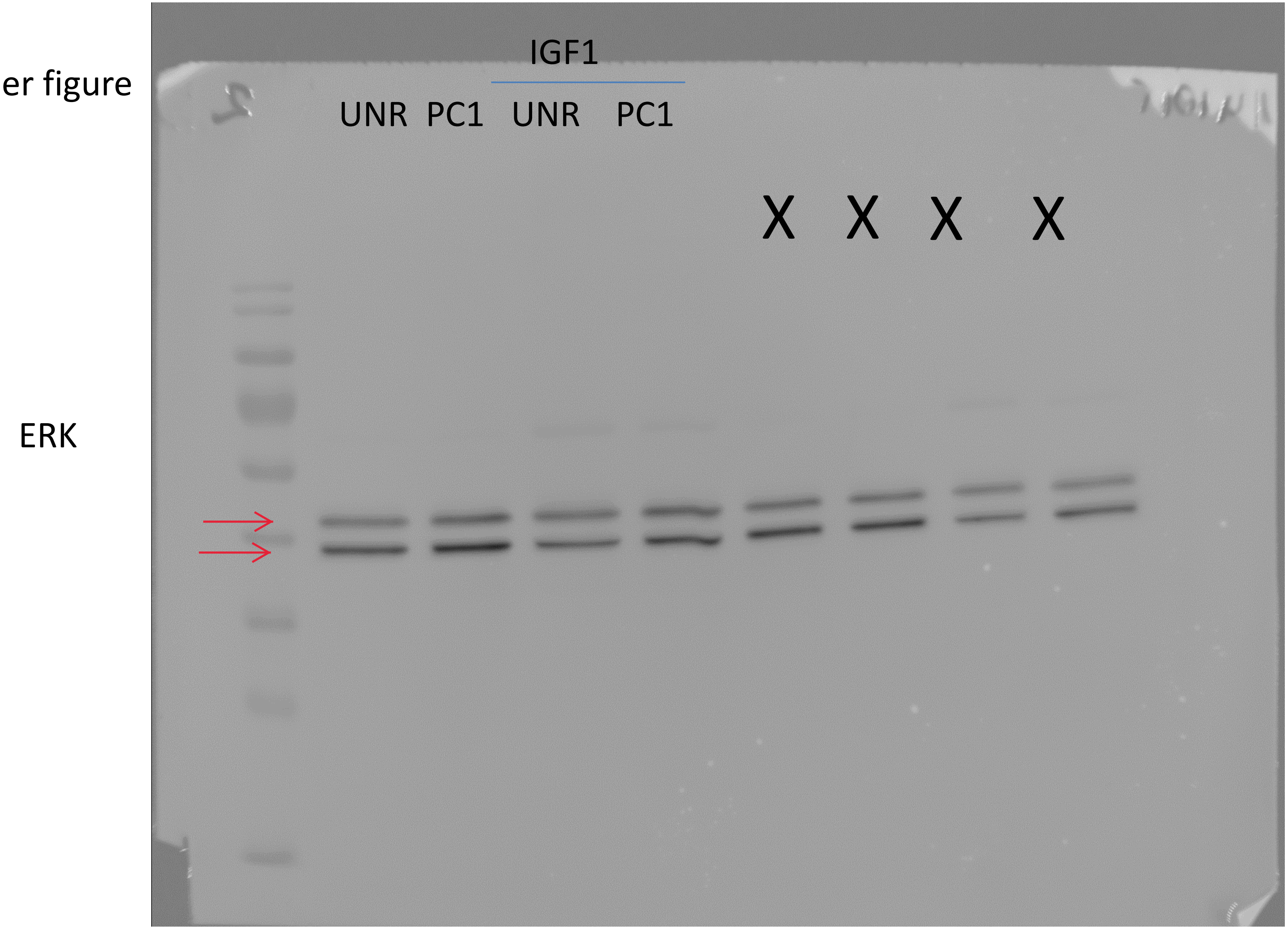

Detection method: EZ-ECL chemiluminescent detection HRP activity, automated

Fig 3C

n2

Paper figure

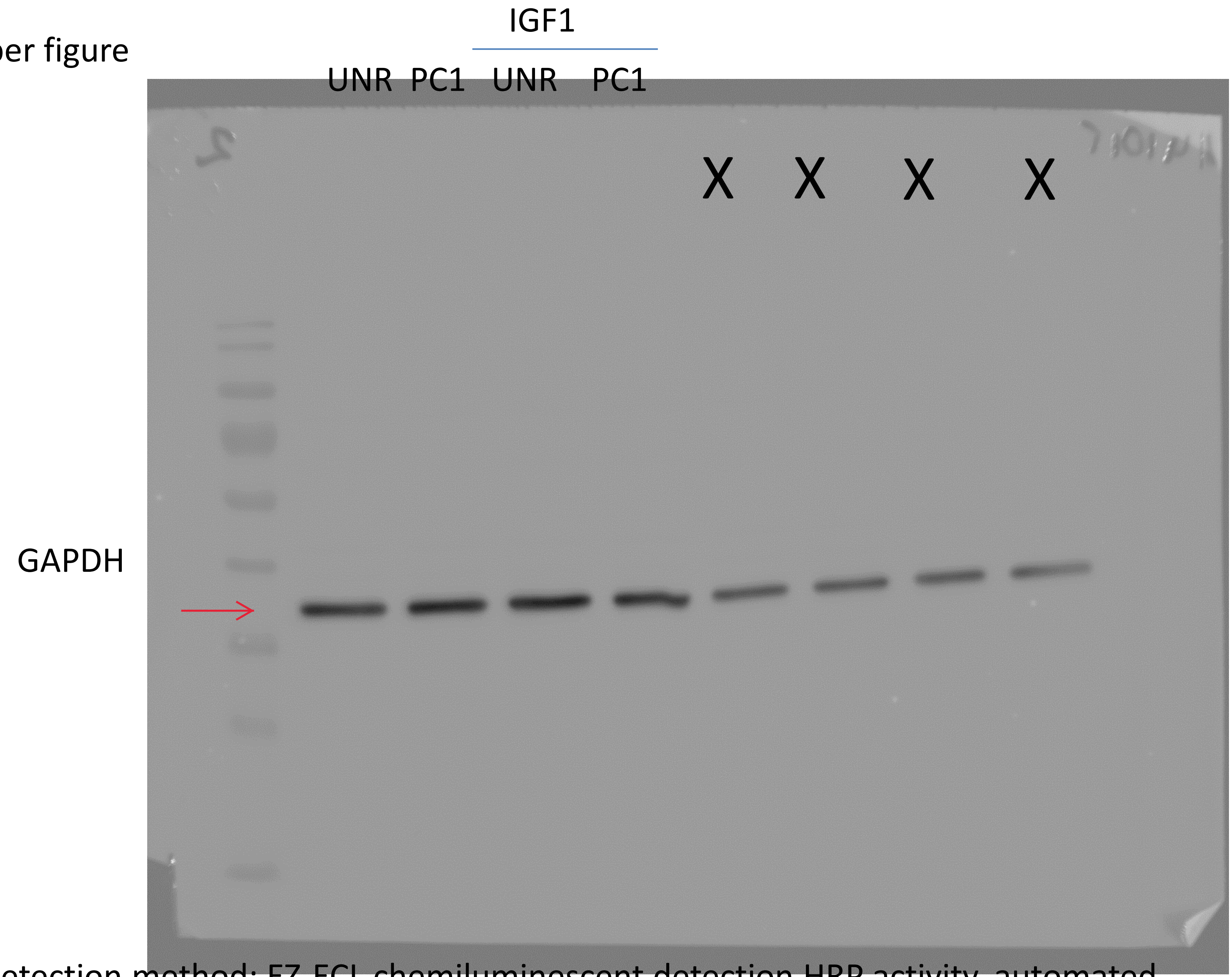

Detection method: EZ-ECL chemiluminescent detection HRP activity, automated

Fig 3C

n3

IGF1

UNR PC1 UNR PC1

pERK

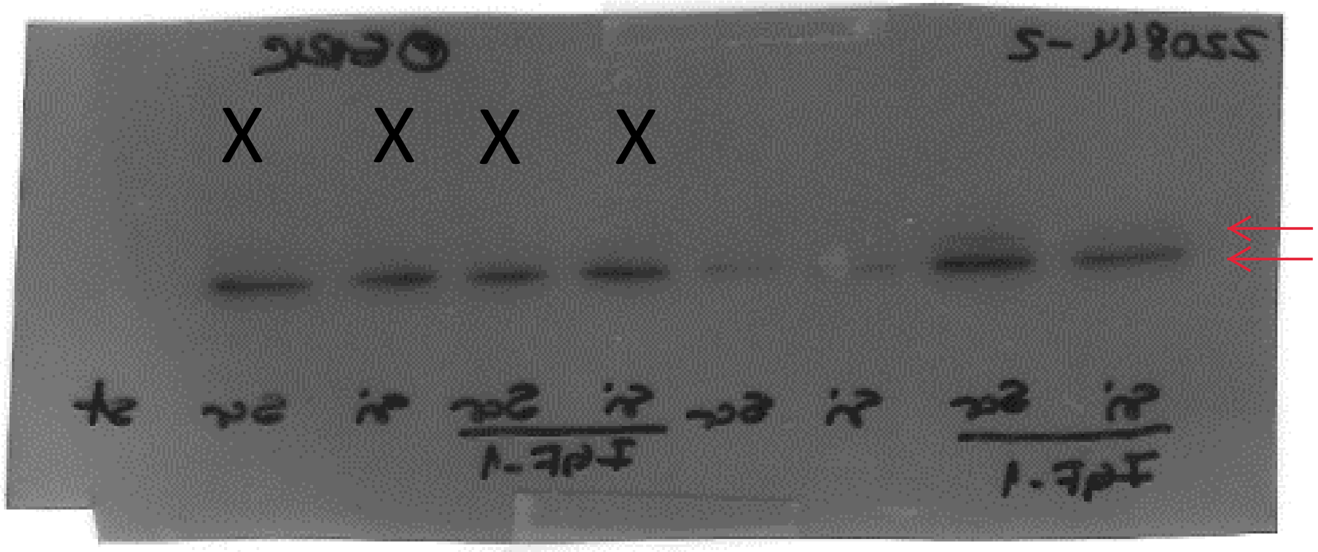

Detection method: EZ-ECL chemiluminescent detection HRP activity, manual development

Fig 3C

n3

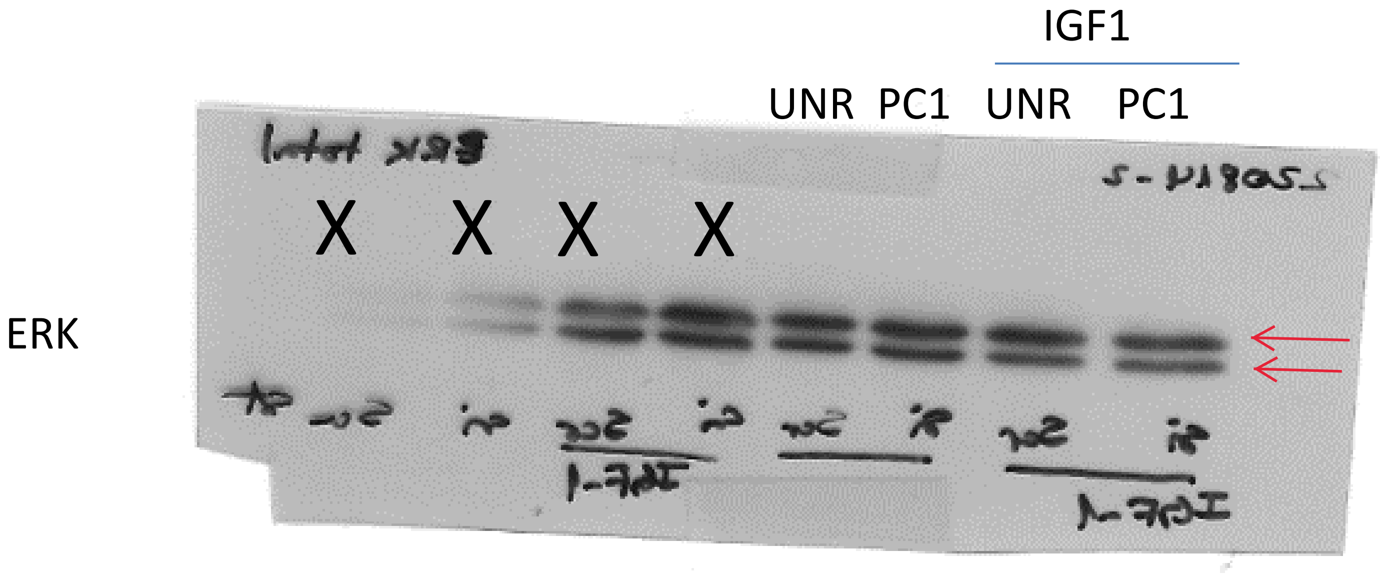

Detection method: EZ-ECL chemiluminescent detection HRP activity, manual development

Fig 3C

n3

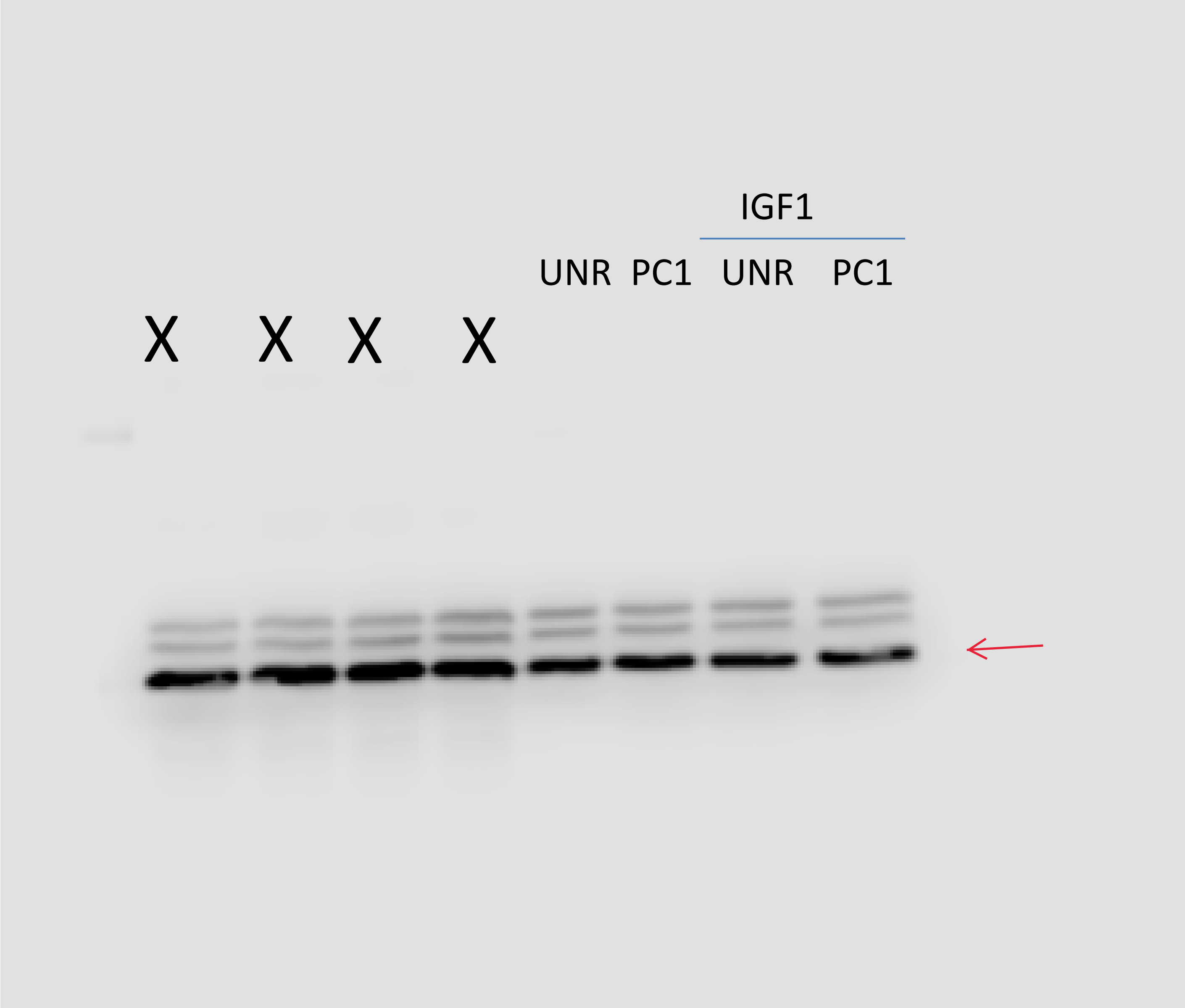

GAPDH

Detection method: EZ-ECL chemiluminescent detection HRP activity, automated

Fig 3C

n4

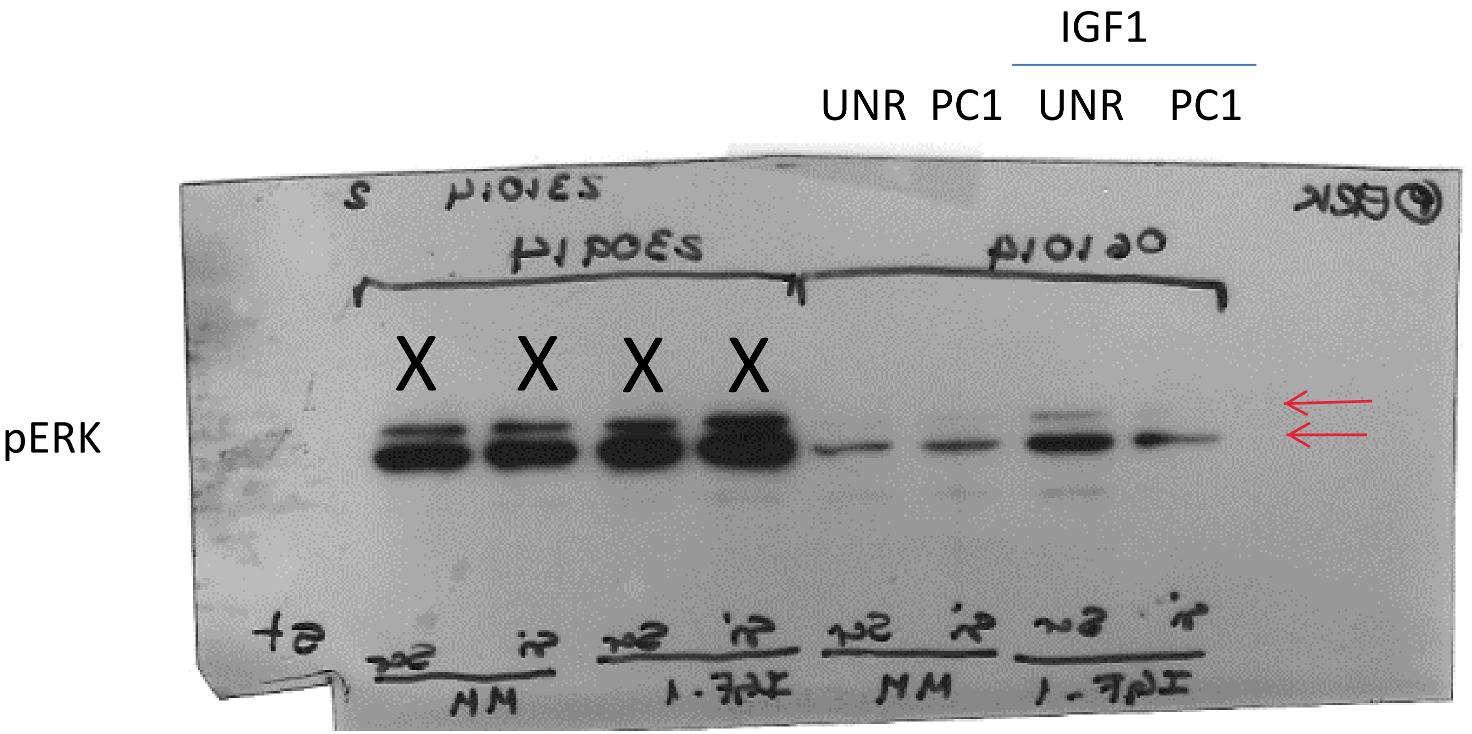

Detection method: EZ-ECL chemiluminescent detection HRP activity, manual development

Fig 3C

n4

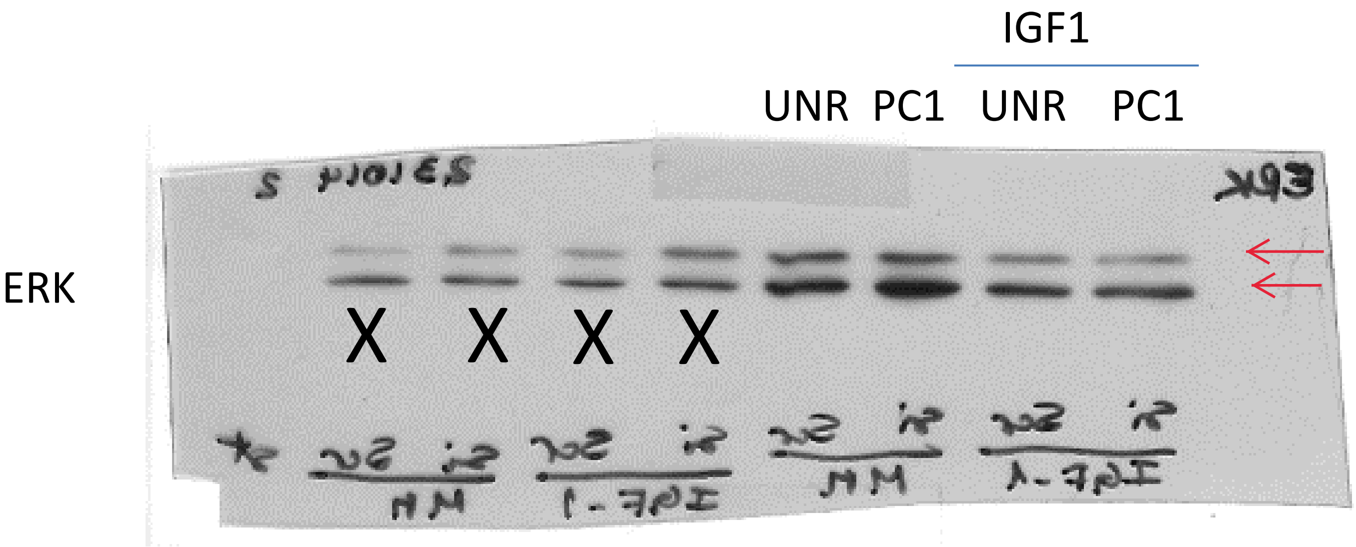

Detection method: EZ-ECL chemiluminescent detection HRP activity, manual development

Fig 3C

n4

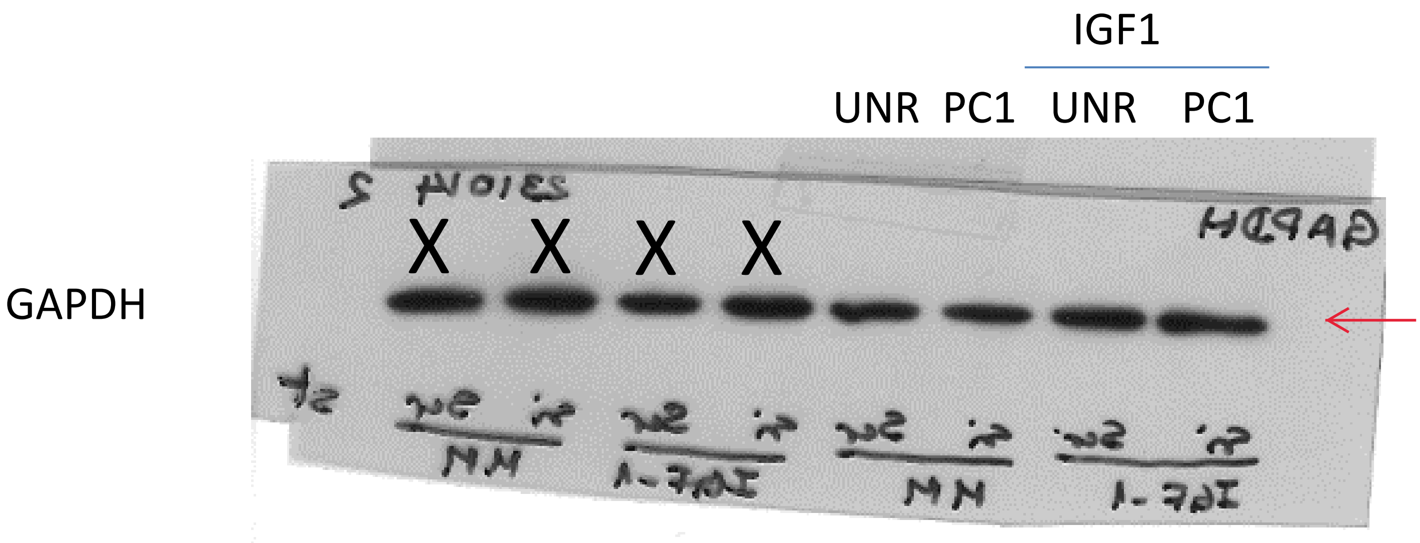

Detection method: EZ-ECL chemiluminescent detection HRP activity, manual development

Fig 3D
n1

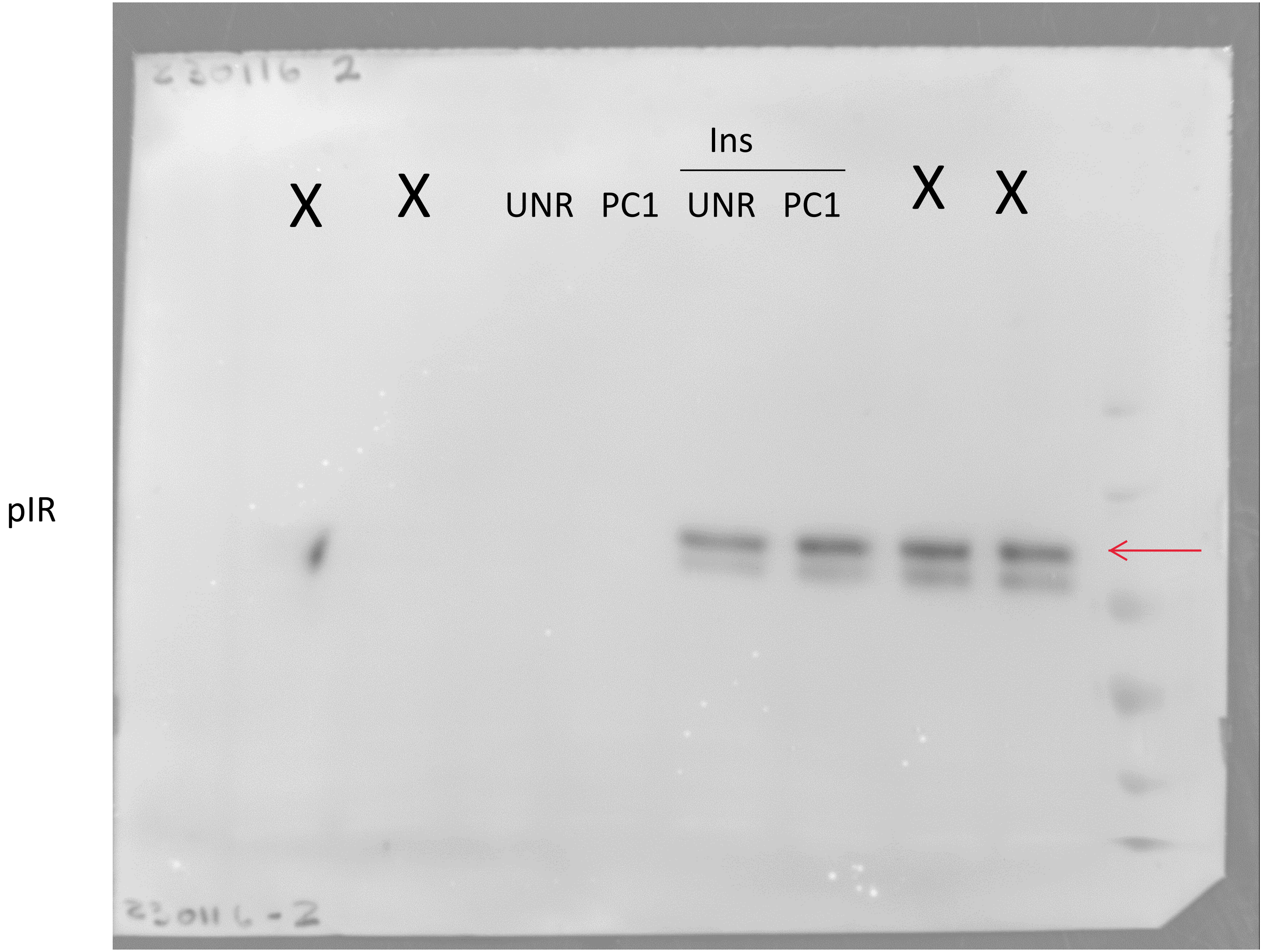

Detection method: EZ-ECL chemiluminescent detection HRP activity, automated

Fig 3D
n1

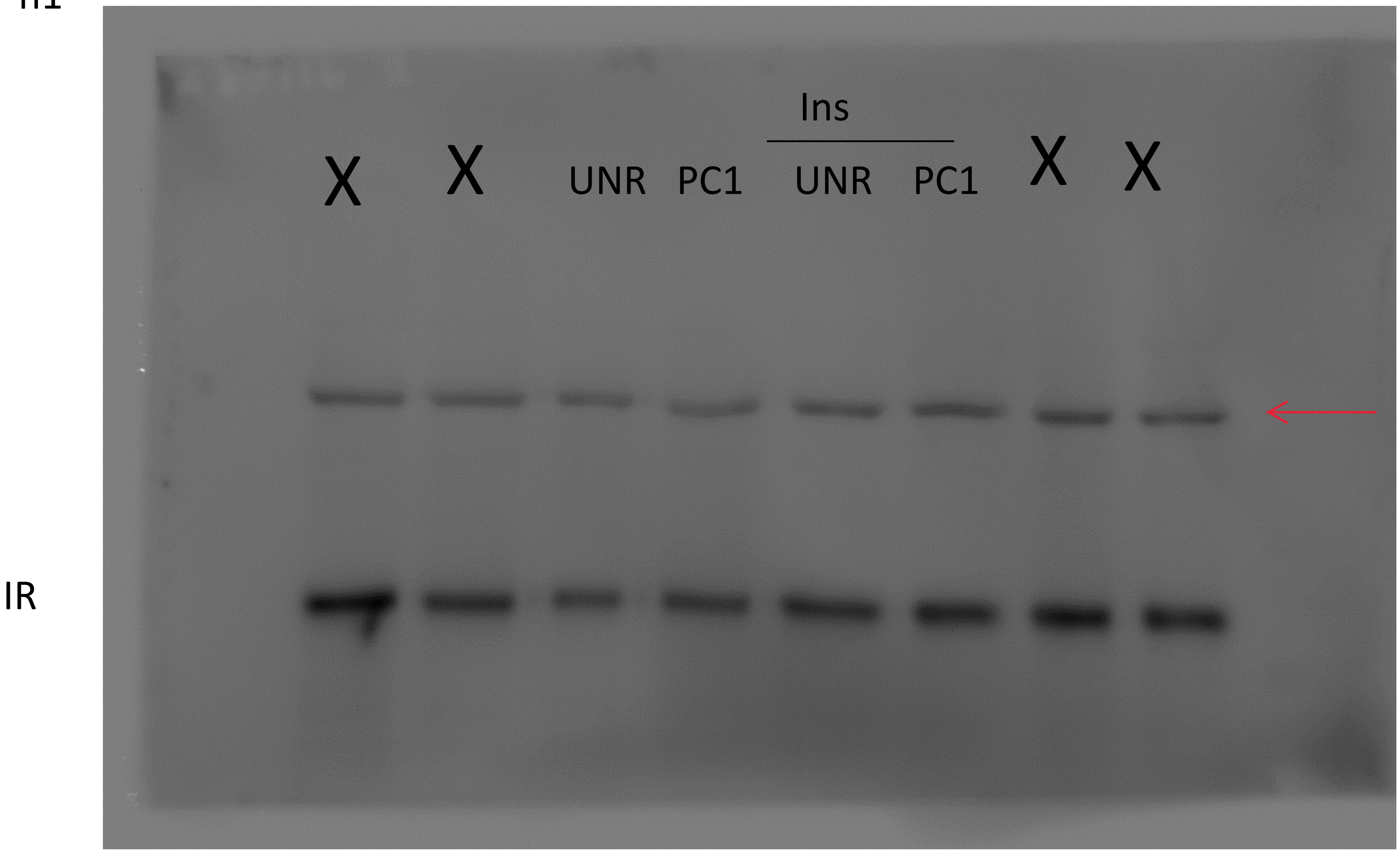

Detection method: EZ-ECL chemiluminescent detection HRP activity, automated

Fig 3D
n2

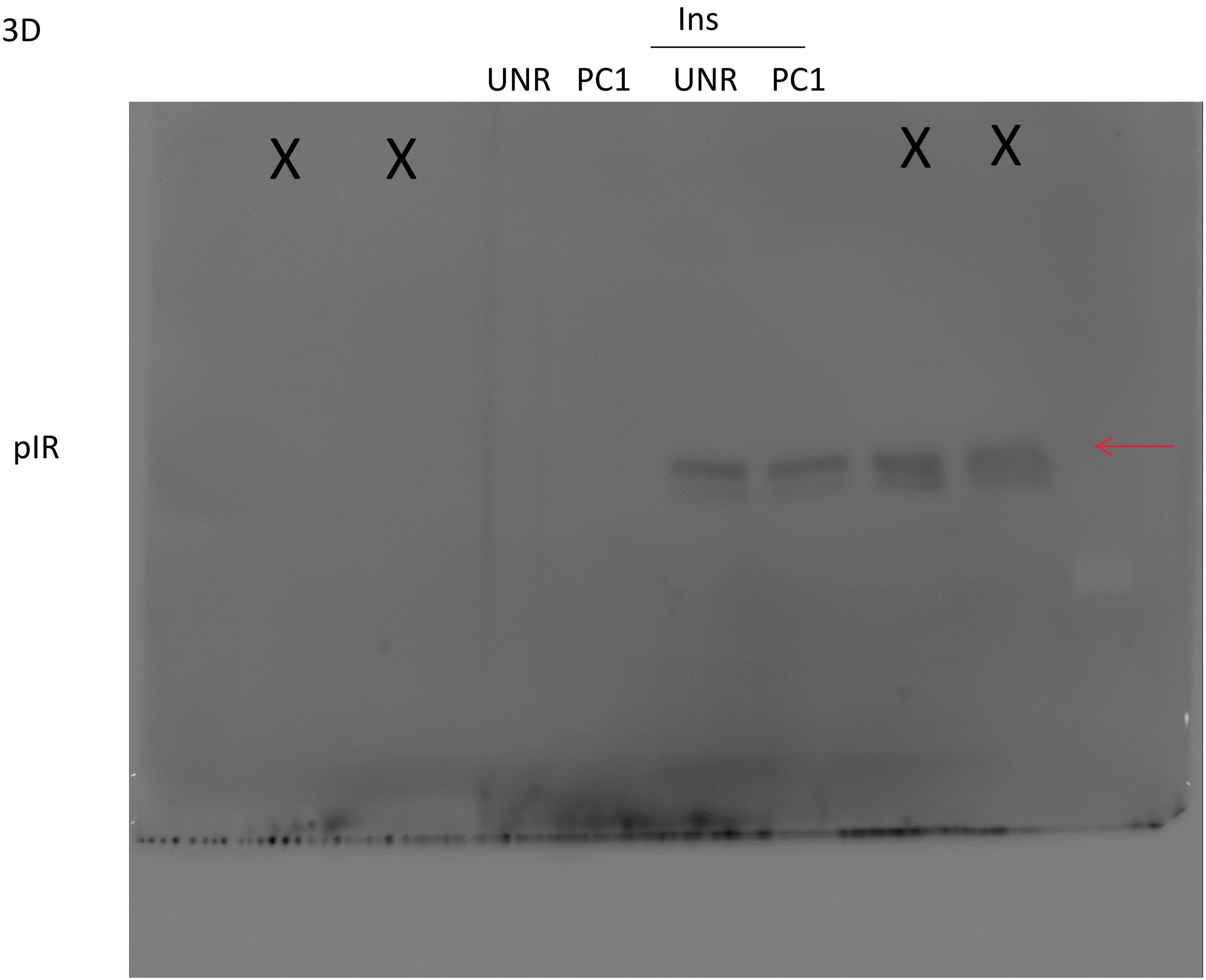

Detection method: EZ-ECL chemiluminescent detection HRP activity, automated

Fig 3D
n2

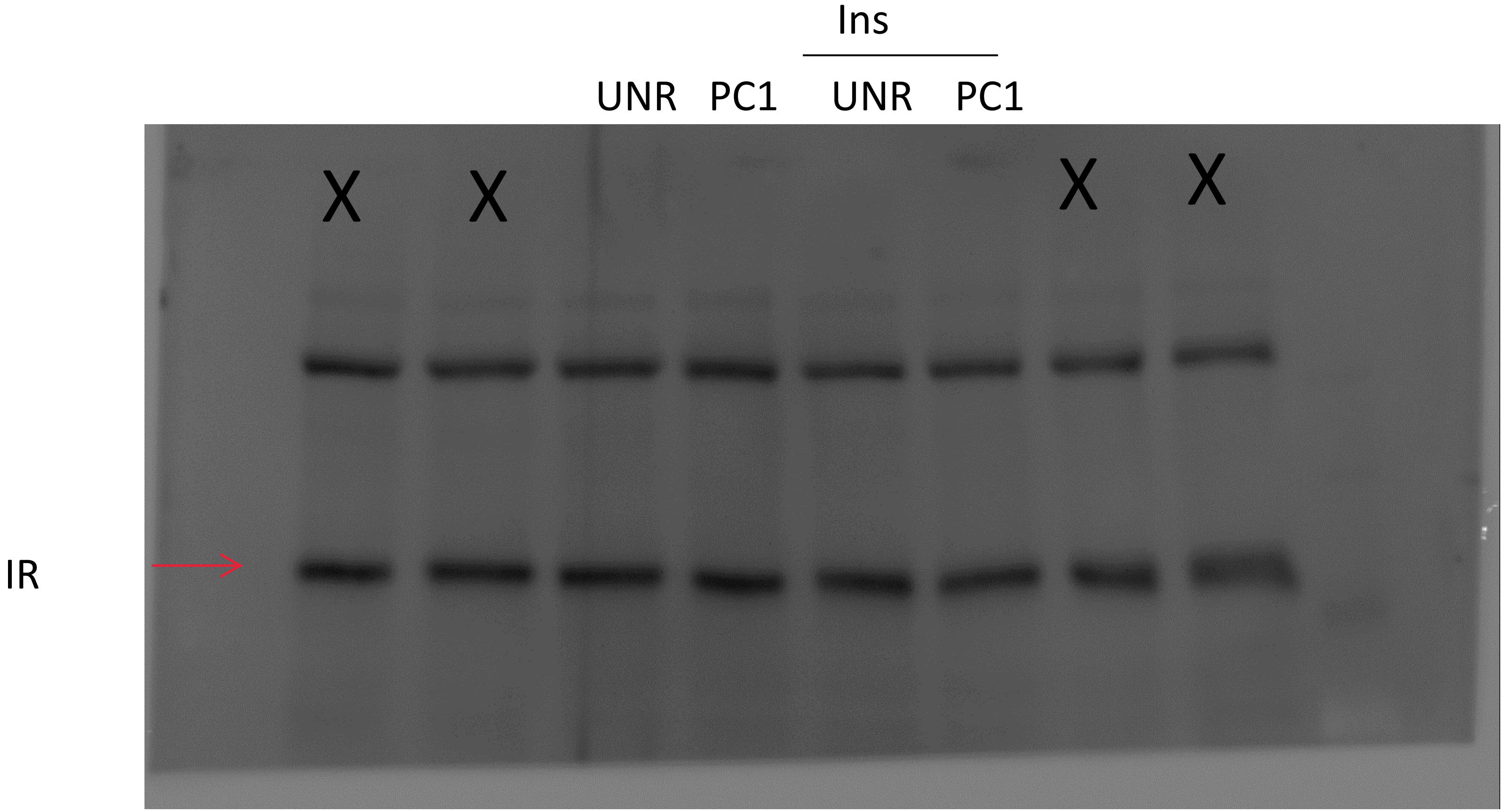

Detection method: EZ-ECL chemiluminescent detection HRP activity, automated

Fig 3D
n3 paper figure

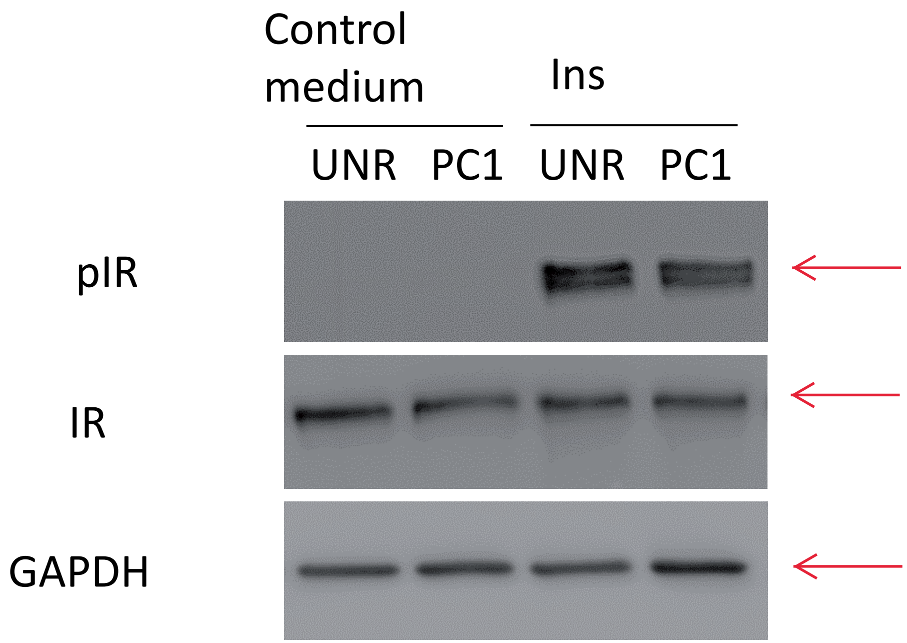

Detection method: EZ-ECL chemiluminescent detection HRP activity, automated

Fig 4A
n1

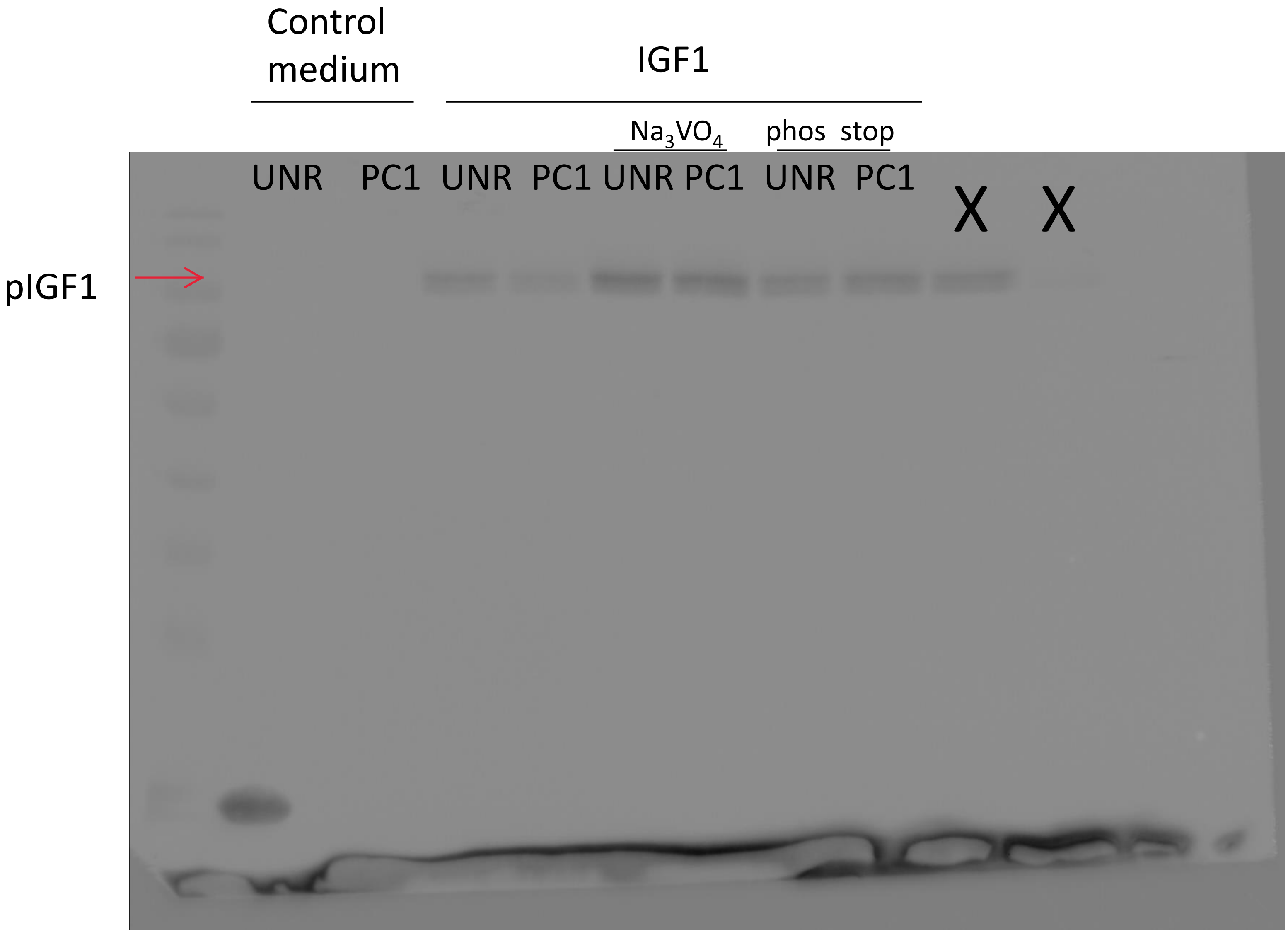

Detection method: EZ-ECL chemiluminescent detection HRP activity, automated

Fig 4A
n1

Control medium | IGF1

Na$_3$VO$_4$ | phos stop

UNR PC1 UNR PC1 UNR PC1 UNR PC1

IGF1R

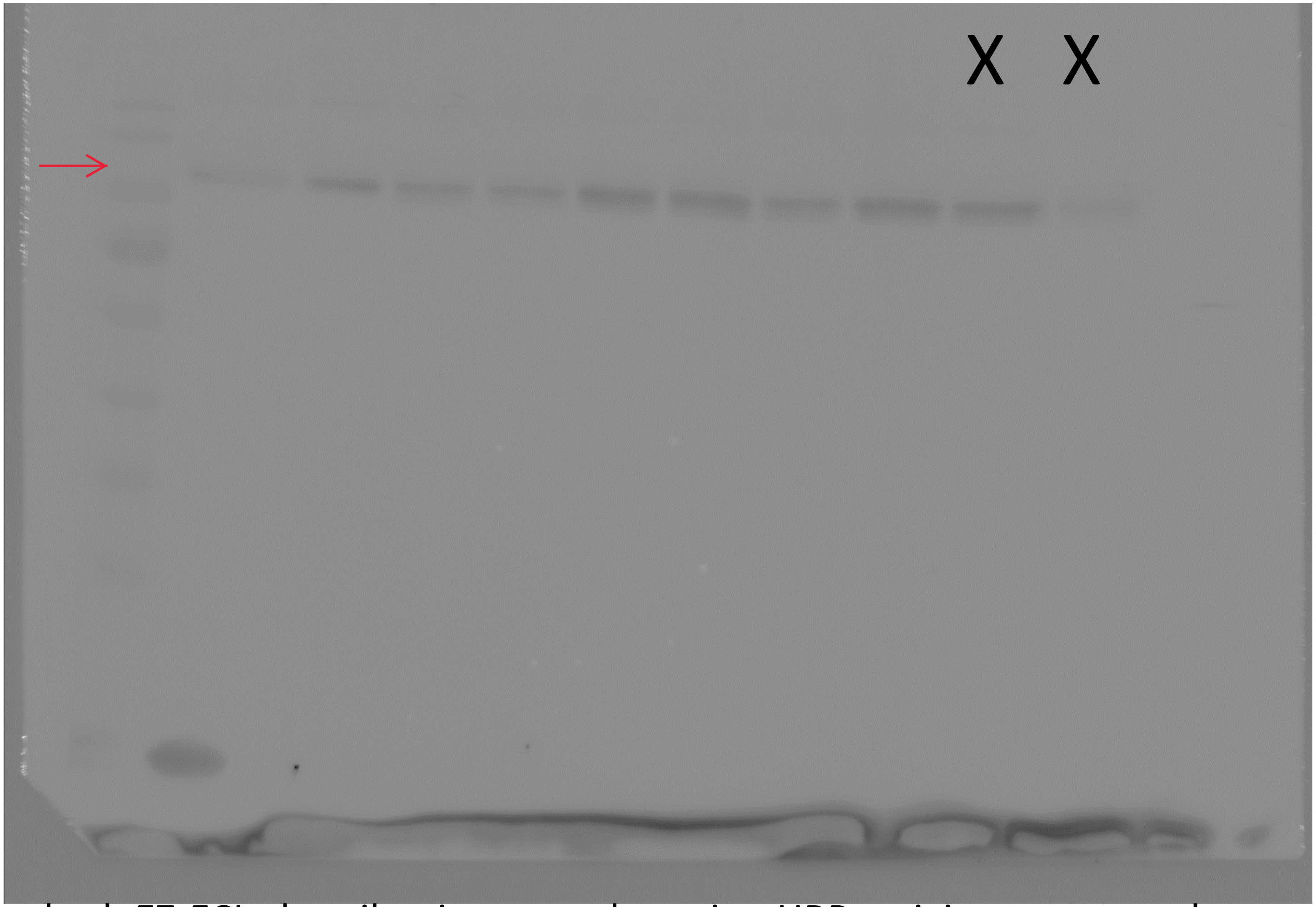

Detection method: EZ-ECL chemiluminescent detection HRP activity, automated

Fig 4A
n1

Control medium

IGF1

Na_3VO_4 phos stop

UNR PC1 UNR PC1 UNR PC1 UNR PC1

GAPDH

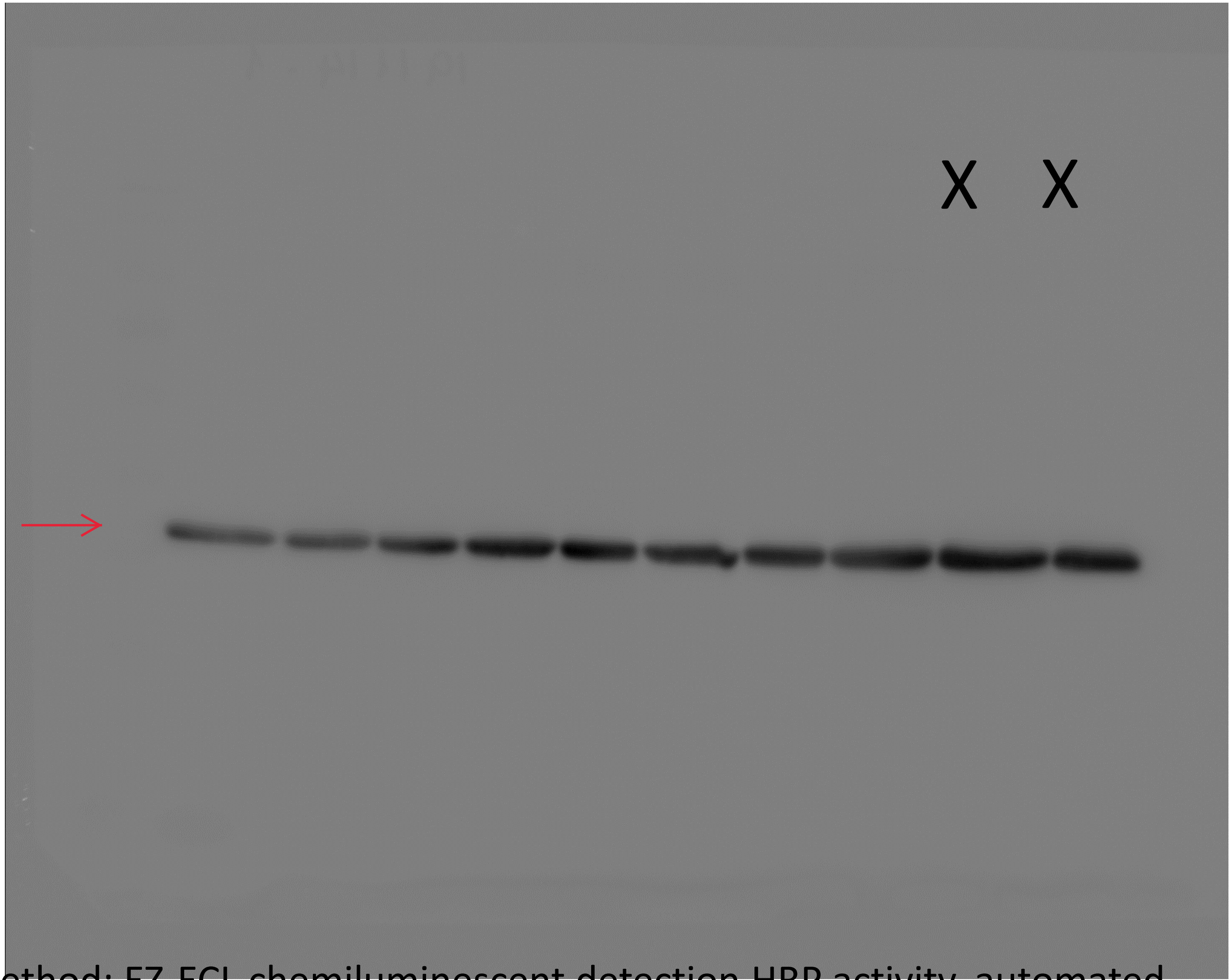

Detection method: EZ-ECL chemiluminescent detection HRP activity, automated

Fig 4A
Paper figure
n2

Control medium | IGF1

Phos stop | Na_3VO_4

UNR PC1 UNR PC1 UNR PC1 UNR PC1

pIGF1

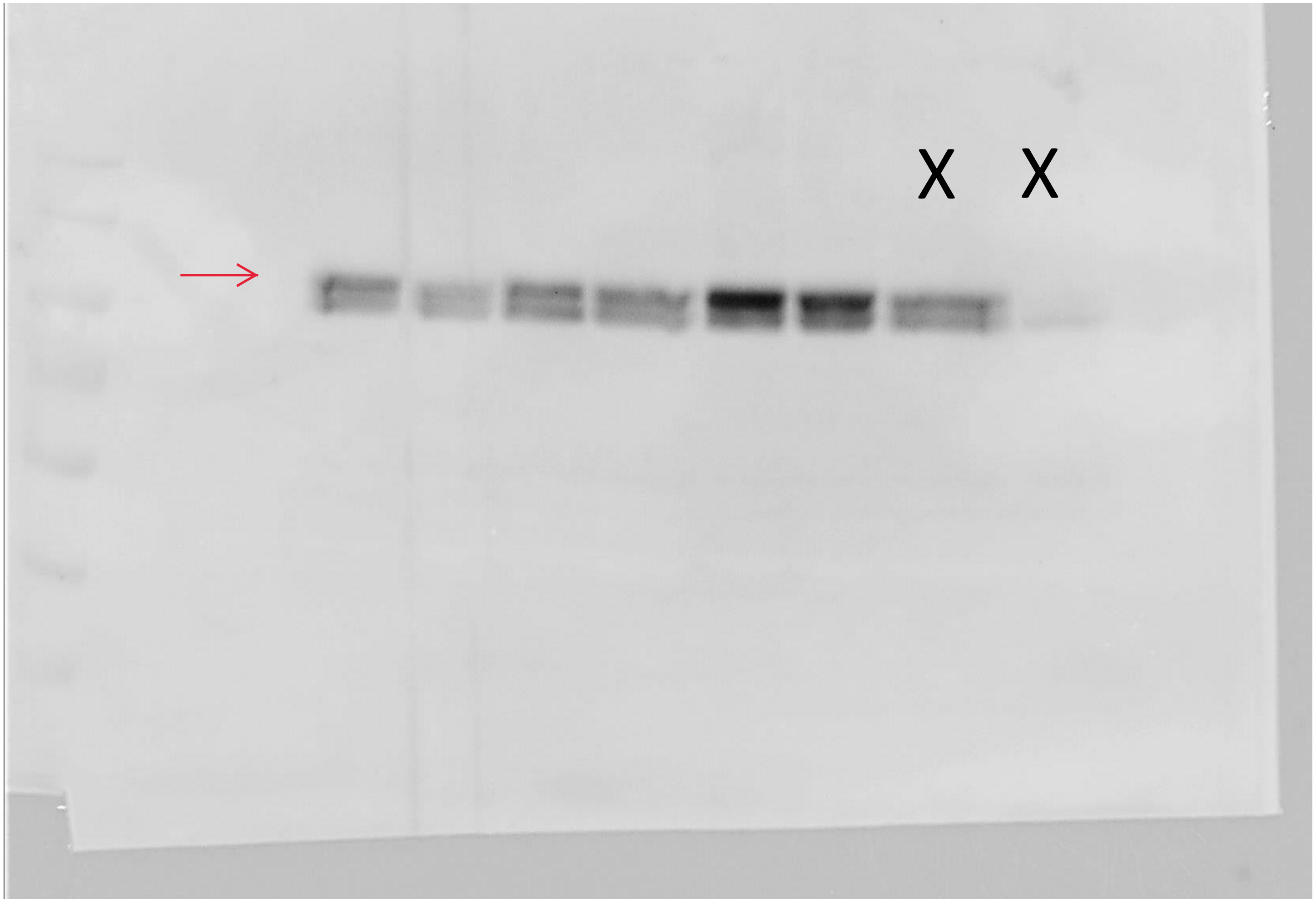

Detection method: EZ-ECL chemiluminescent detection HRP activity, automated

Fig 4A

Paper figure

n2

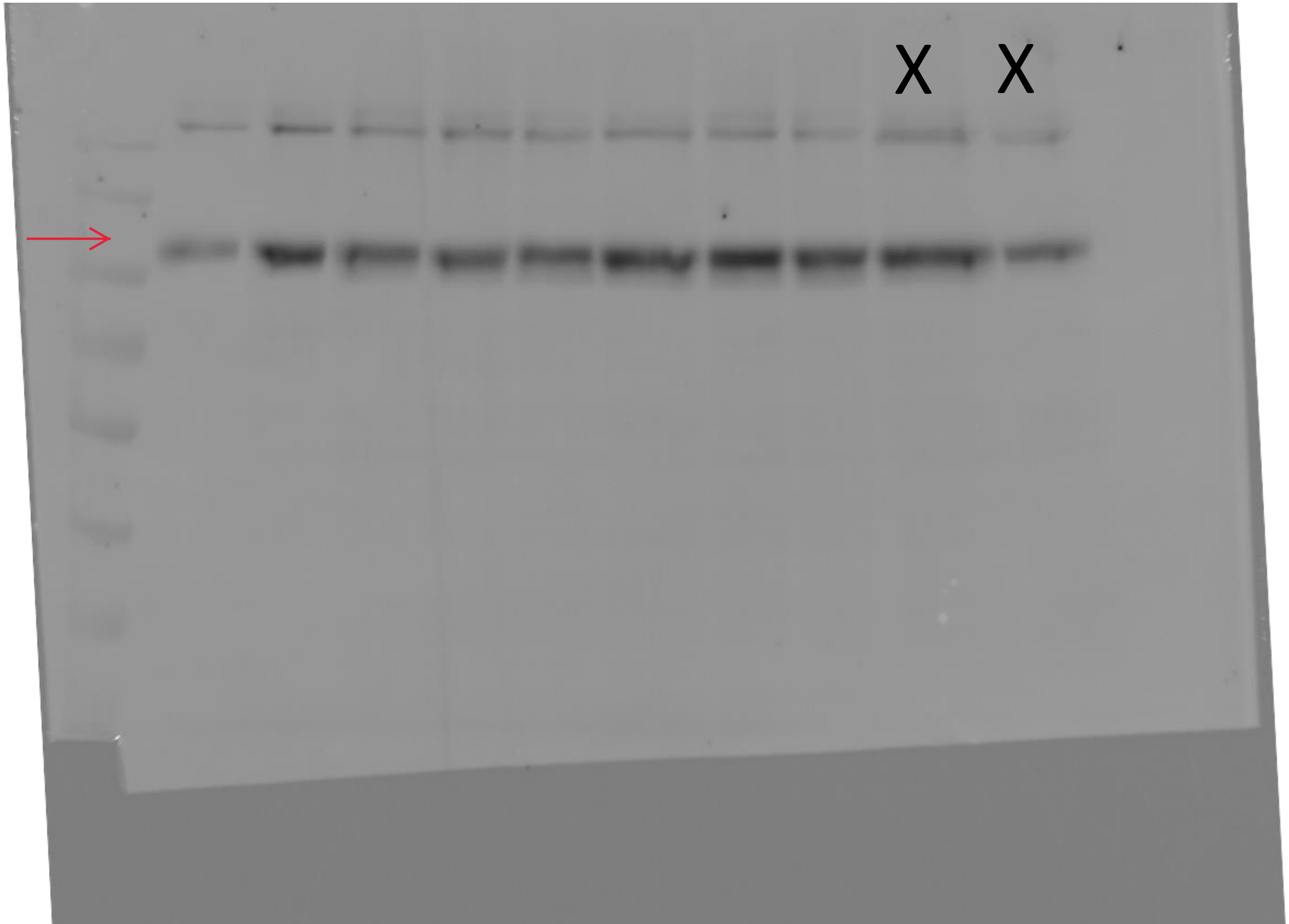

Detection method: EZ-ECL chemiluminescent detection HRP activity, automated

Fig 4A

Paper figure

n2

Control medium

IGF1

Phos stop

Na_3VO_4

UNR PC1 UNR PC1 UNR PC1 UNR PC1

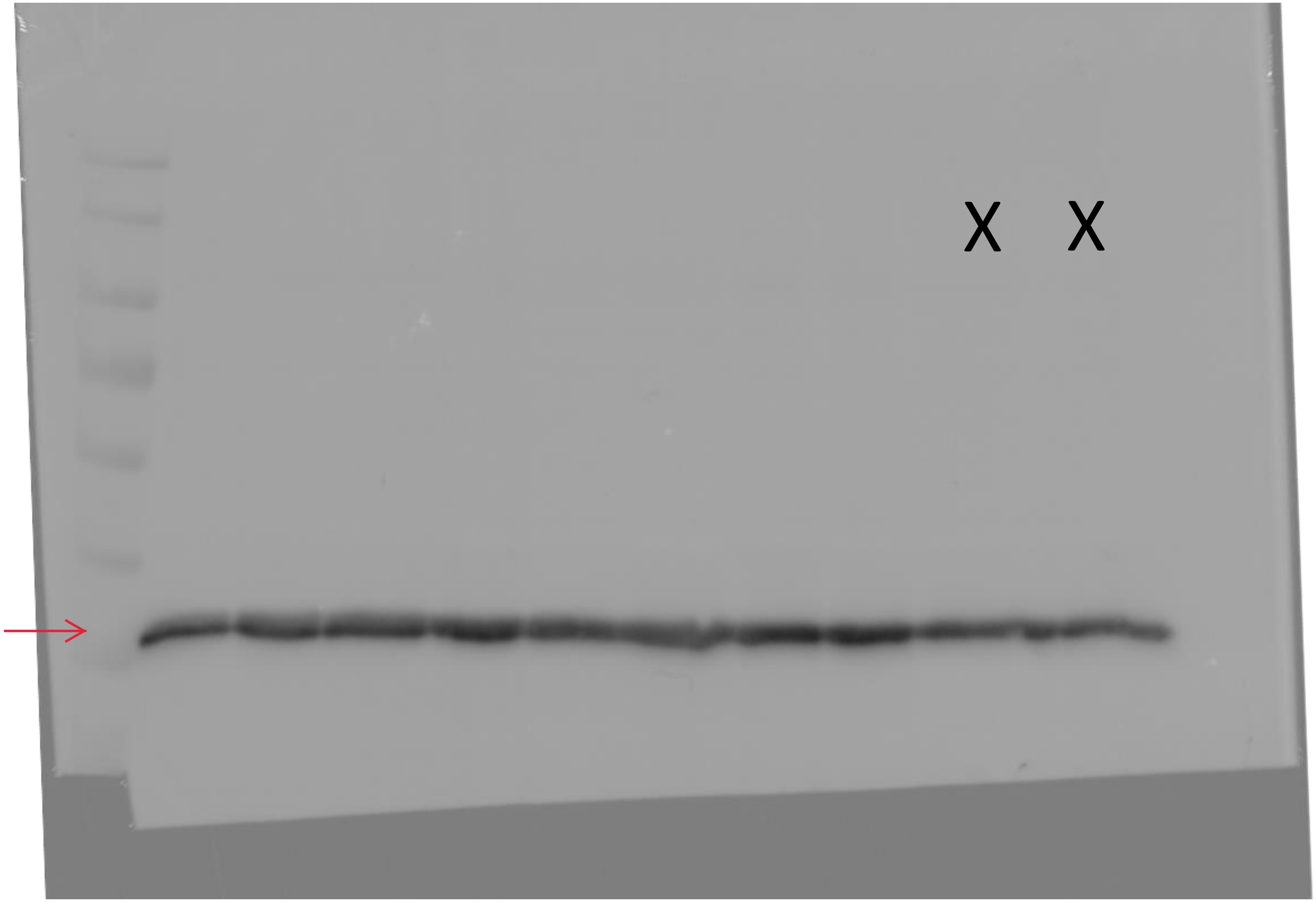

GAPDH

Detection method: EZ-ECL chemiluminescent detection HRP activity, automated

Fig 4A
n3

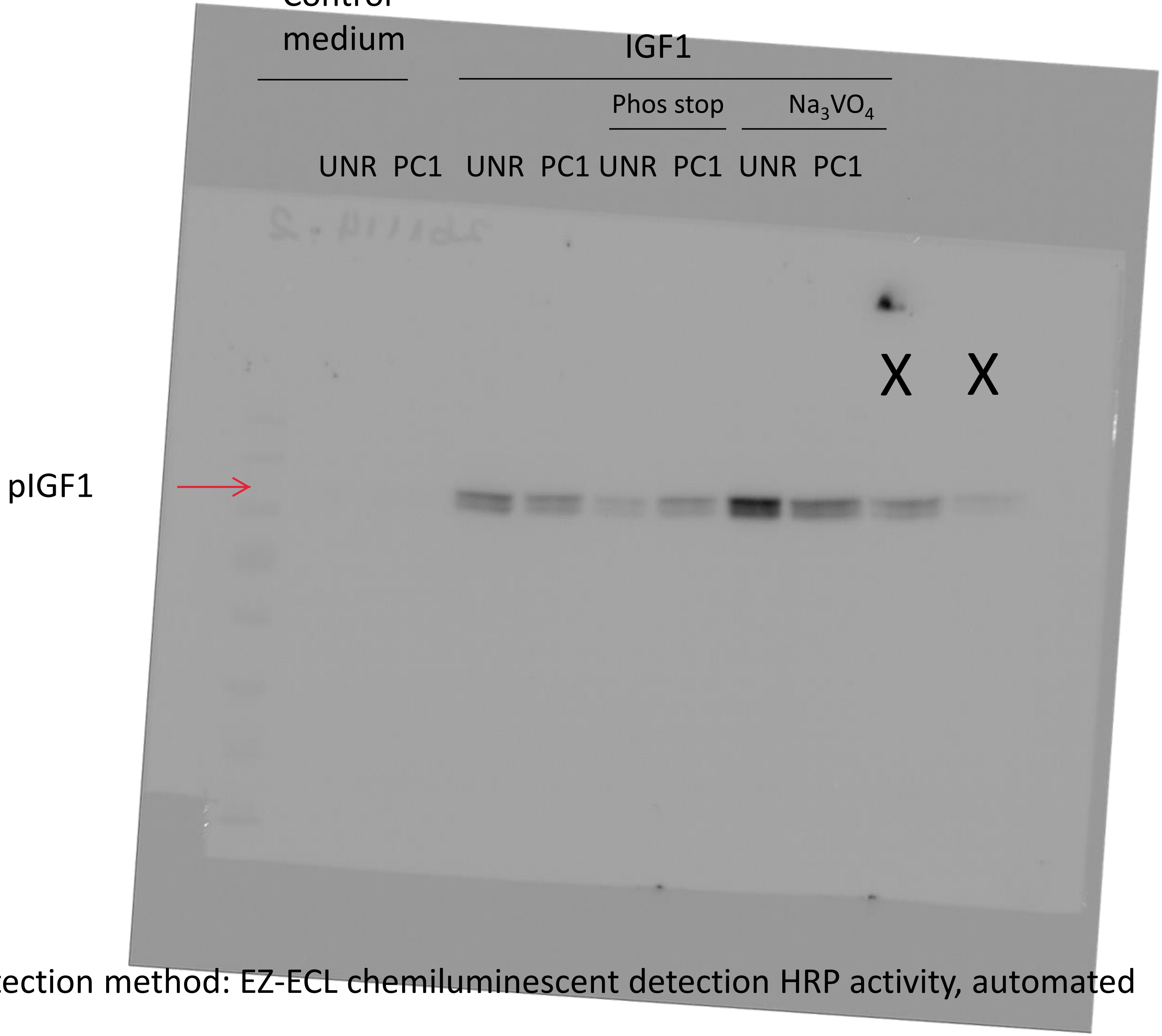

Detection method: EZ-ECL chemiluminescent detection HRP activity, automated

Fig 4A
n3

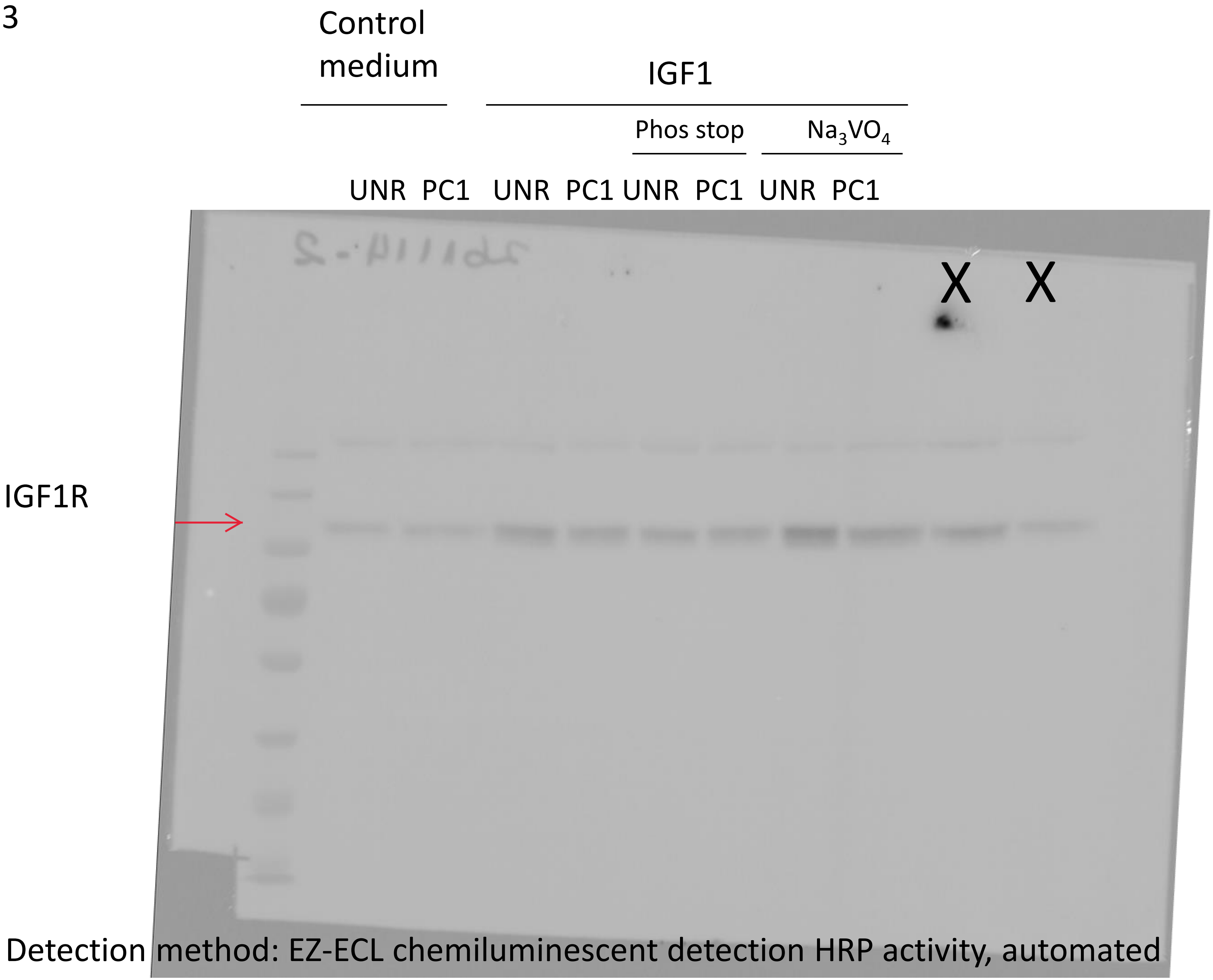

Detection method: EZ-ECL chemiluminescent detection HRP activity, automated

Fig 4A
n3

GAPDH

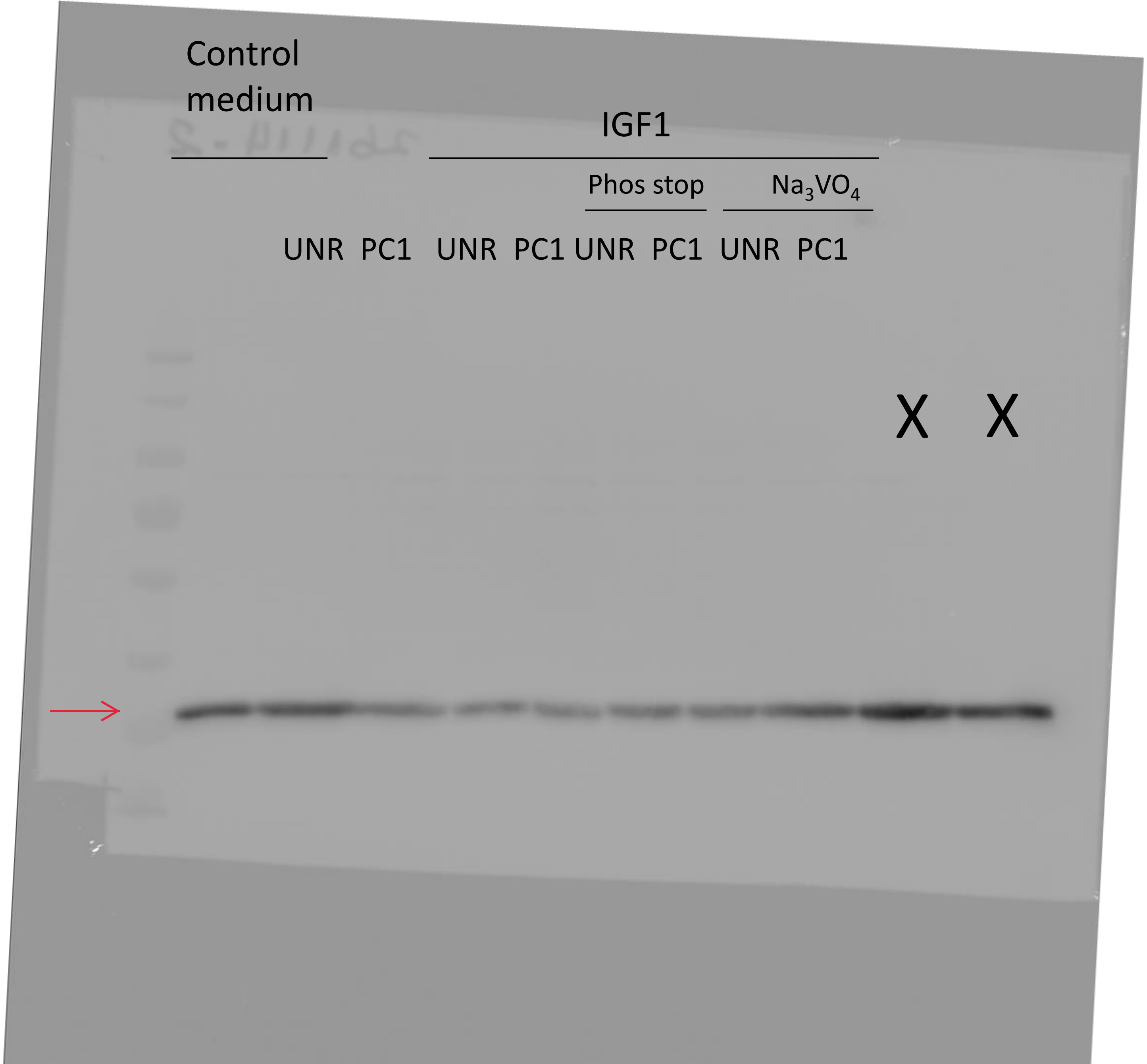

Detection method: EZ-ECL chemiluminescent detection HRP activity, automated

Fig 4A
n4

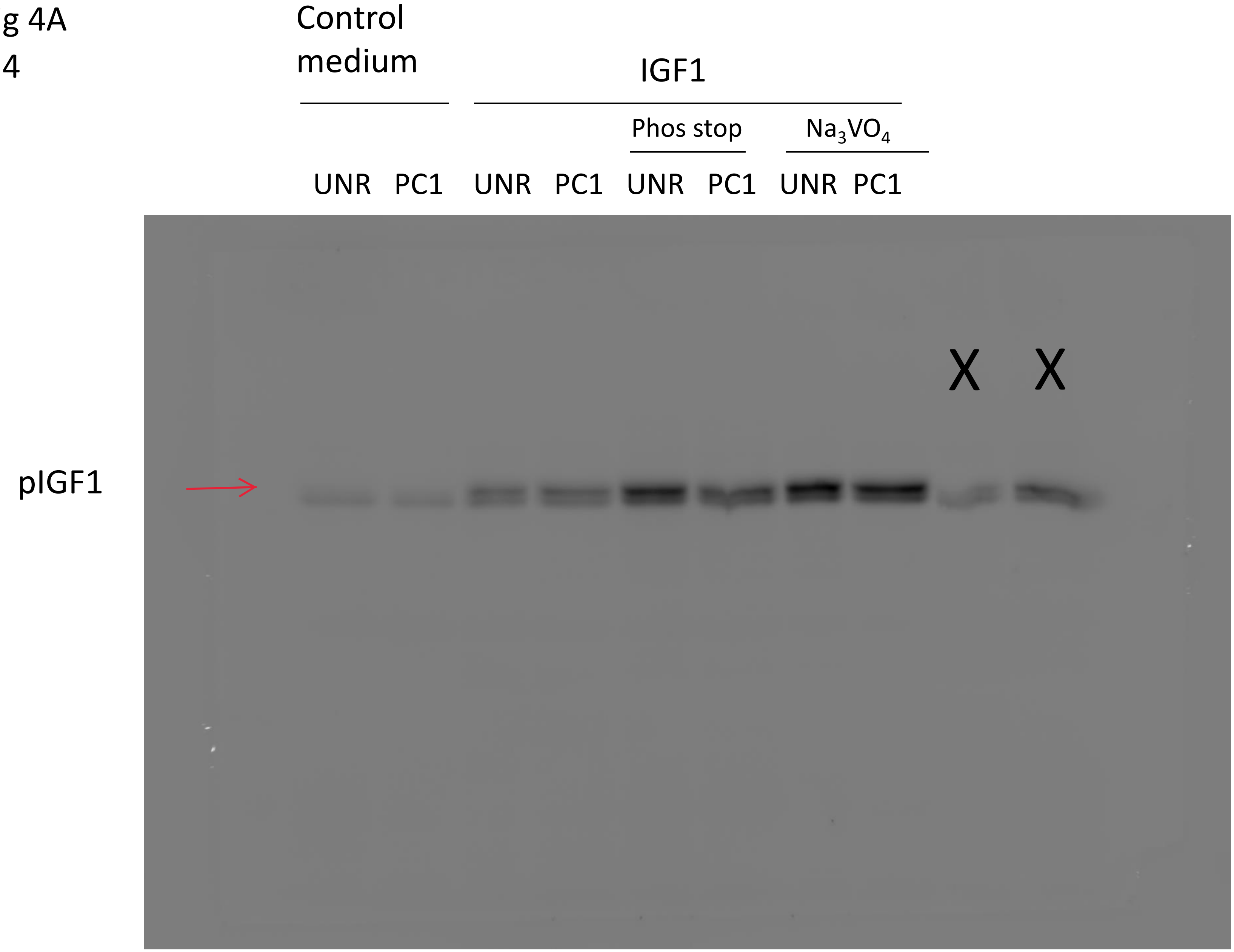

Detection method: EZ-ECL chemiluminescent detection HRP activity, automated

Fig 4A
n4

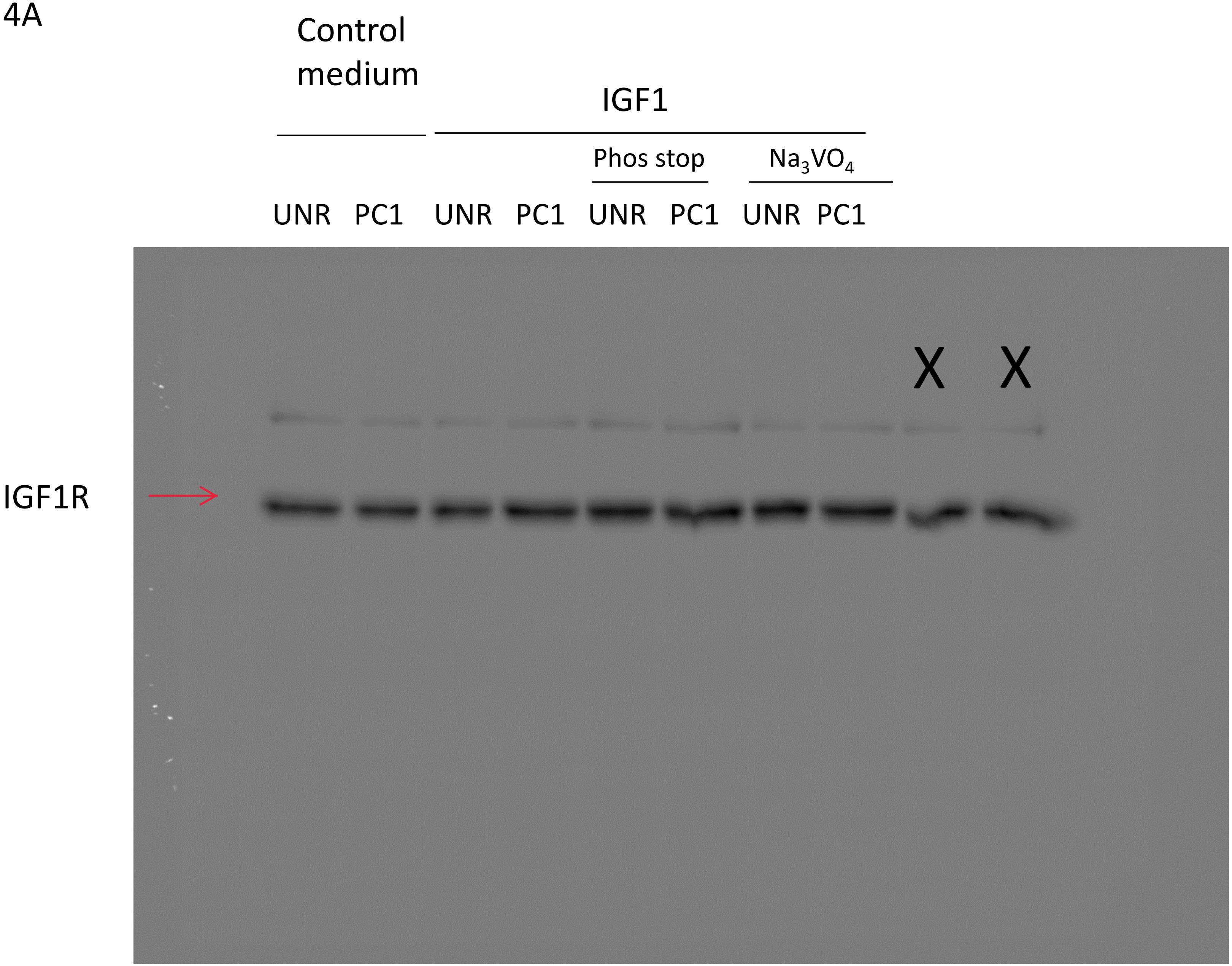

Detection method: EZ-ECL chemiluminescent detection HRP activity, automated

Fig 4A

n4

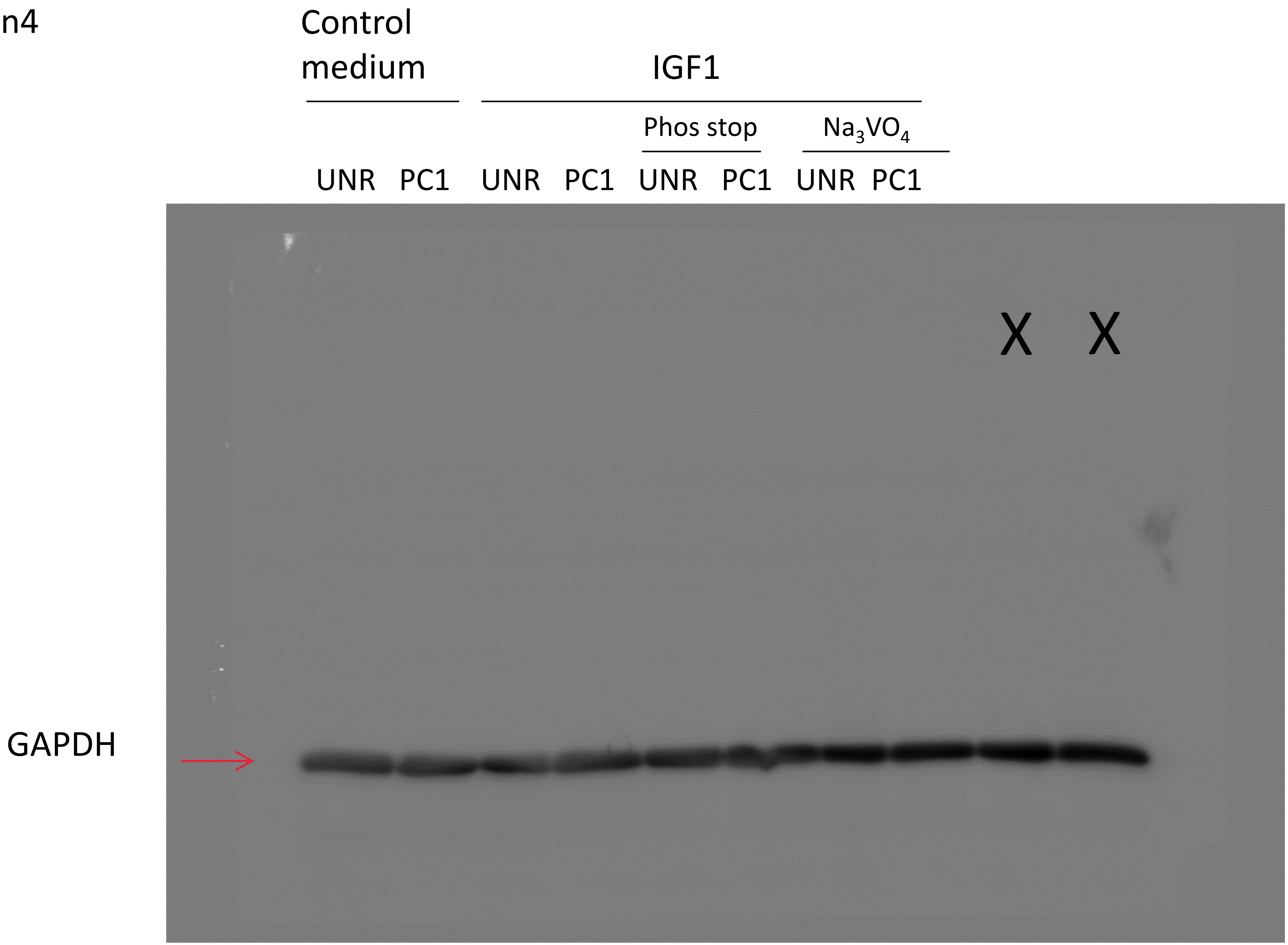

Detection method: EZ-ECL chemiluminescent detection HRP activity, automated

Fig 4B

n1

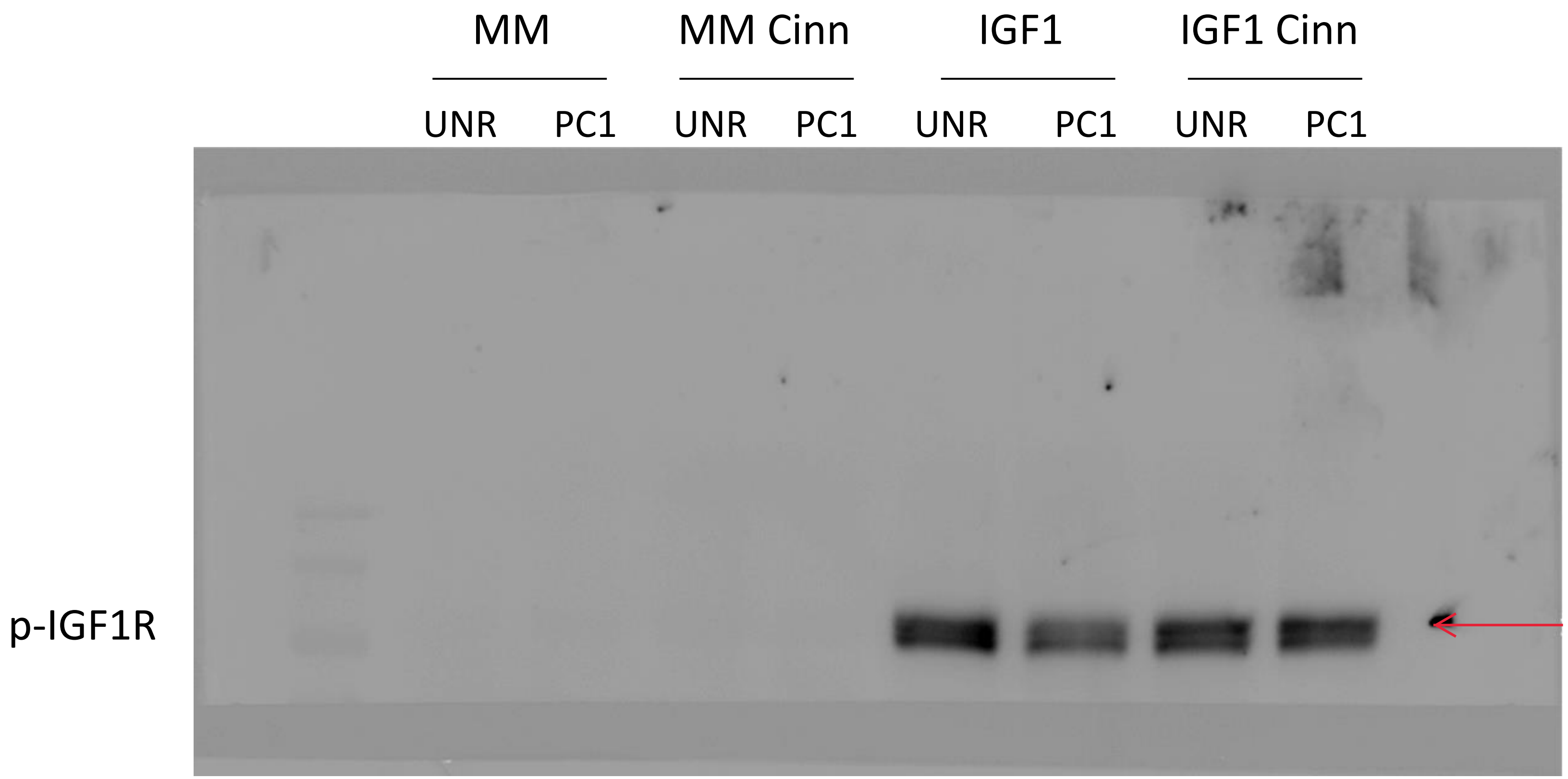

Detection method: EZ-ECL chemiluminescent detection HRP activity, automated

Fig 4B
n1

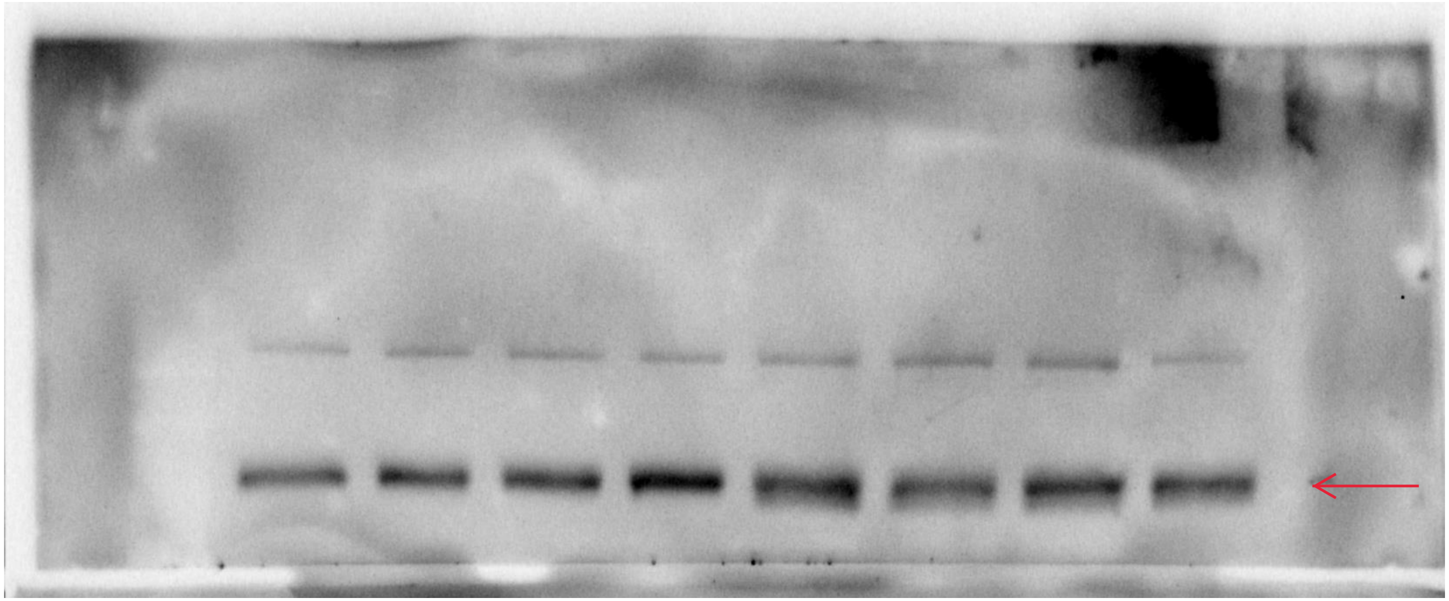

Detection method: EZ-ECL chemiluminescent detection HRP activity, automated

Fig 4B
n1

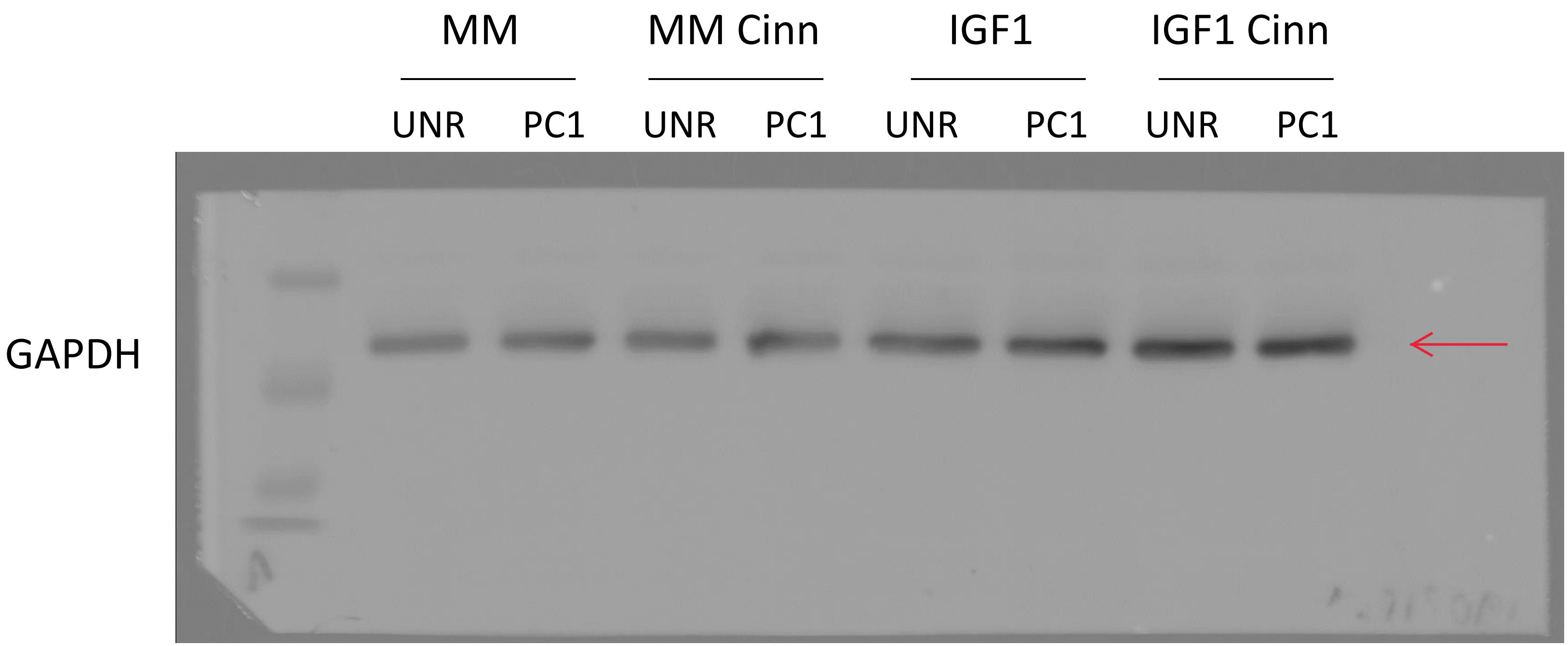

Detection method: EZ-ECL chemiluminescent detection HRP activity, automated

Fig 4B

n2

Paper figure

MM		MM Cinn		IGF1		IGF1 Cinn	
UNR	PC1	UNR	PC1	UNR	PC1	UNR	PC1

p-IGF1R

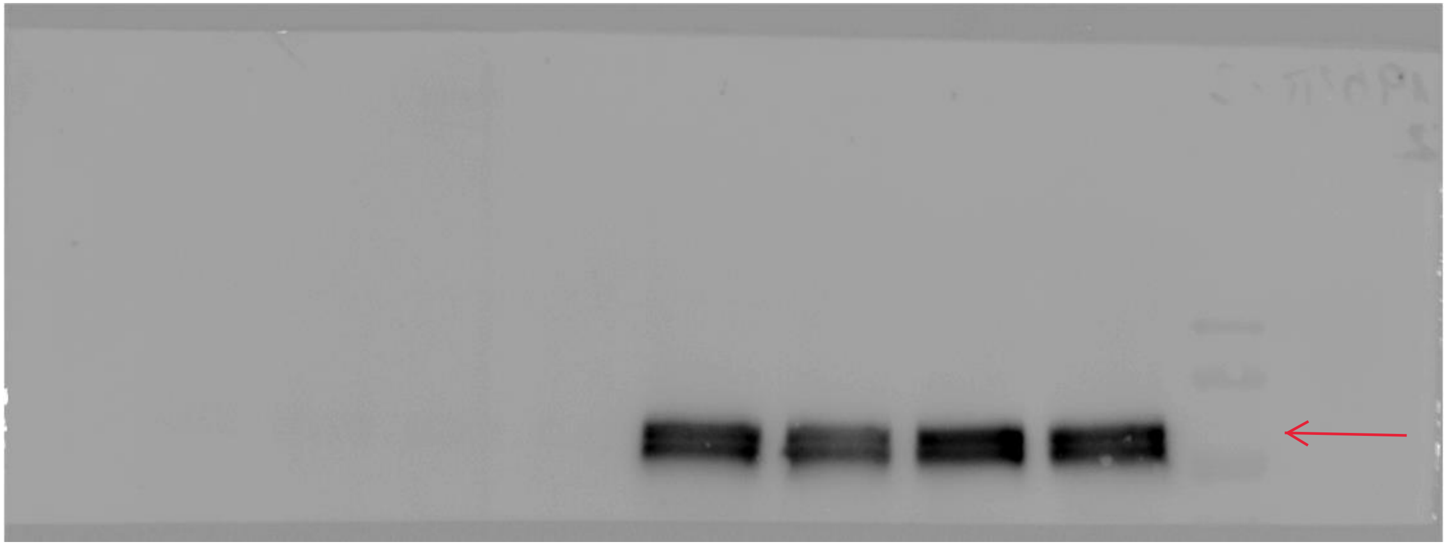

Detection method: EZ-ECL chemiluminescent detection HRP activity, automated

Fig 4B

n2

Paper figure

MM | MM Cinn | IGF1 | IGF1 Cinn

UNR PC1 UNR PC1 UNR PC1 UNR PC1

IGF1R

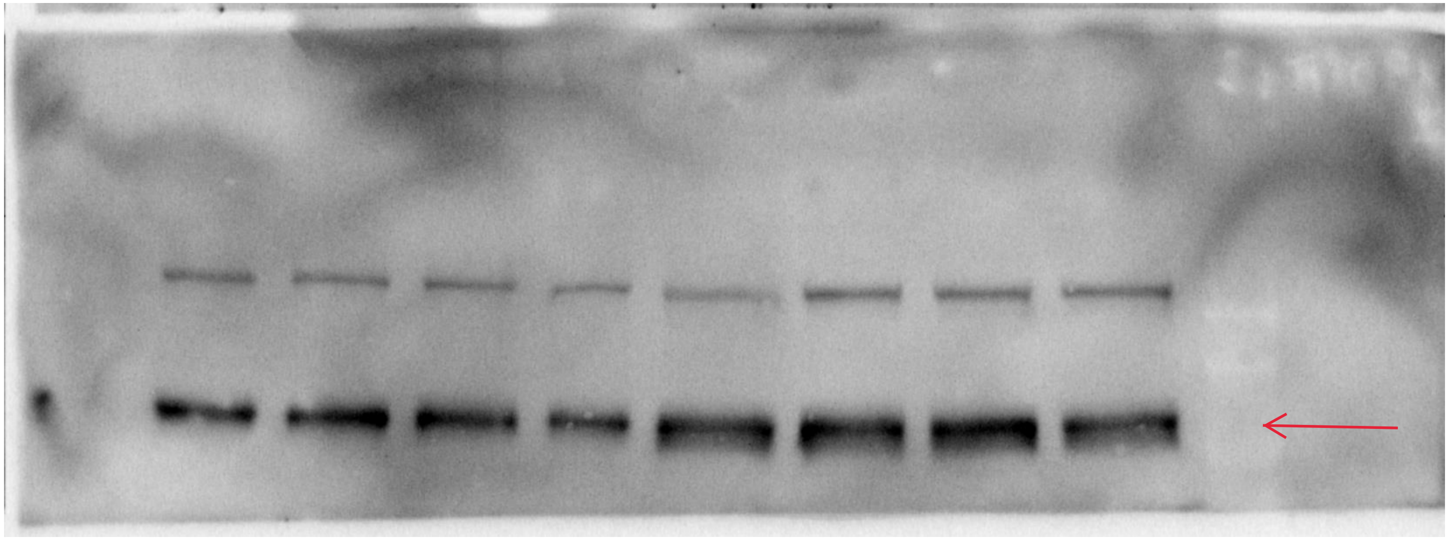

Detection method: EZ-ECL chemiluminescent detection HRP activity, automated

Fig 4B

n2

Paper figure

MM		MM Cinn		IGF1		IGF1 Cinn	
UNR	PC1	UNR	PC1	UNR	PC1	UNR	PC1

GAPDH

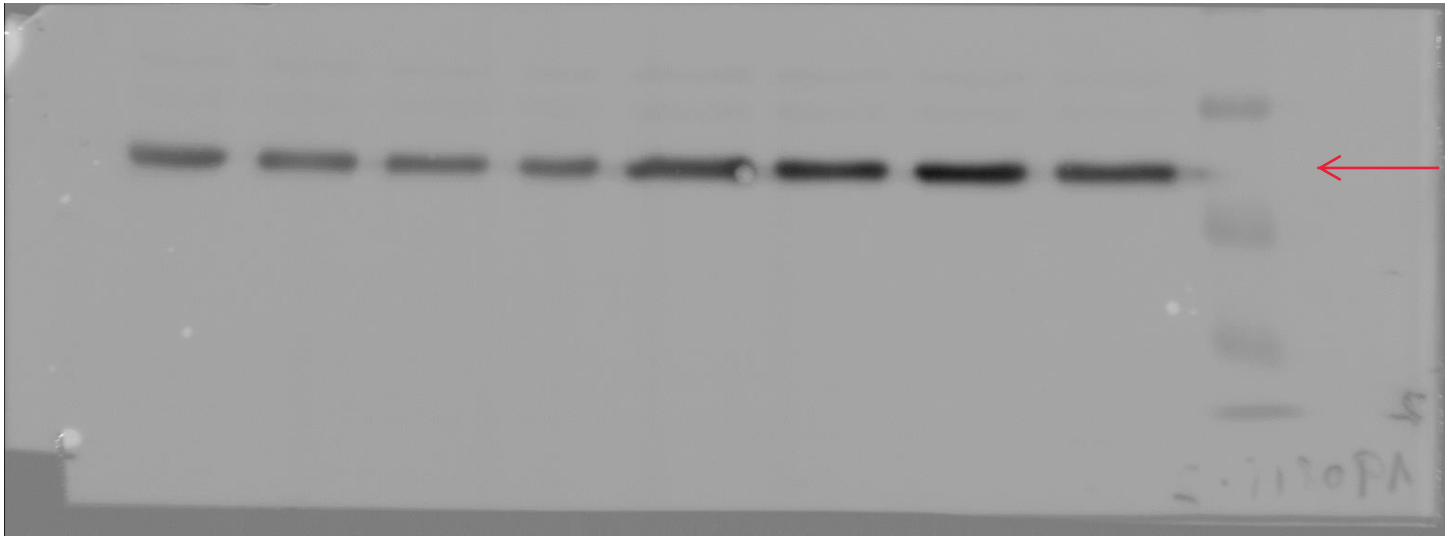

Detection method: EZ-ECL chemiluminescent detection HRP activity, automated

Fig 4B
n3

IGF1 — UNR, PC1
IGF1 Cinn — UNR, PC1

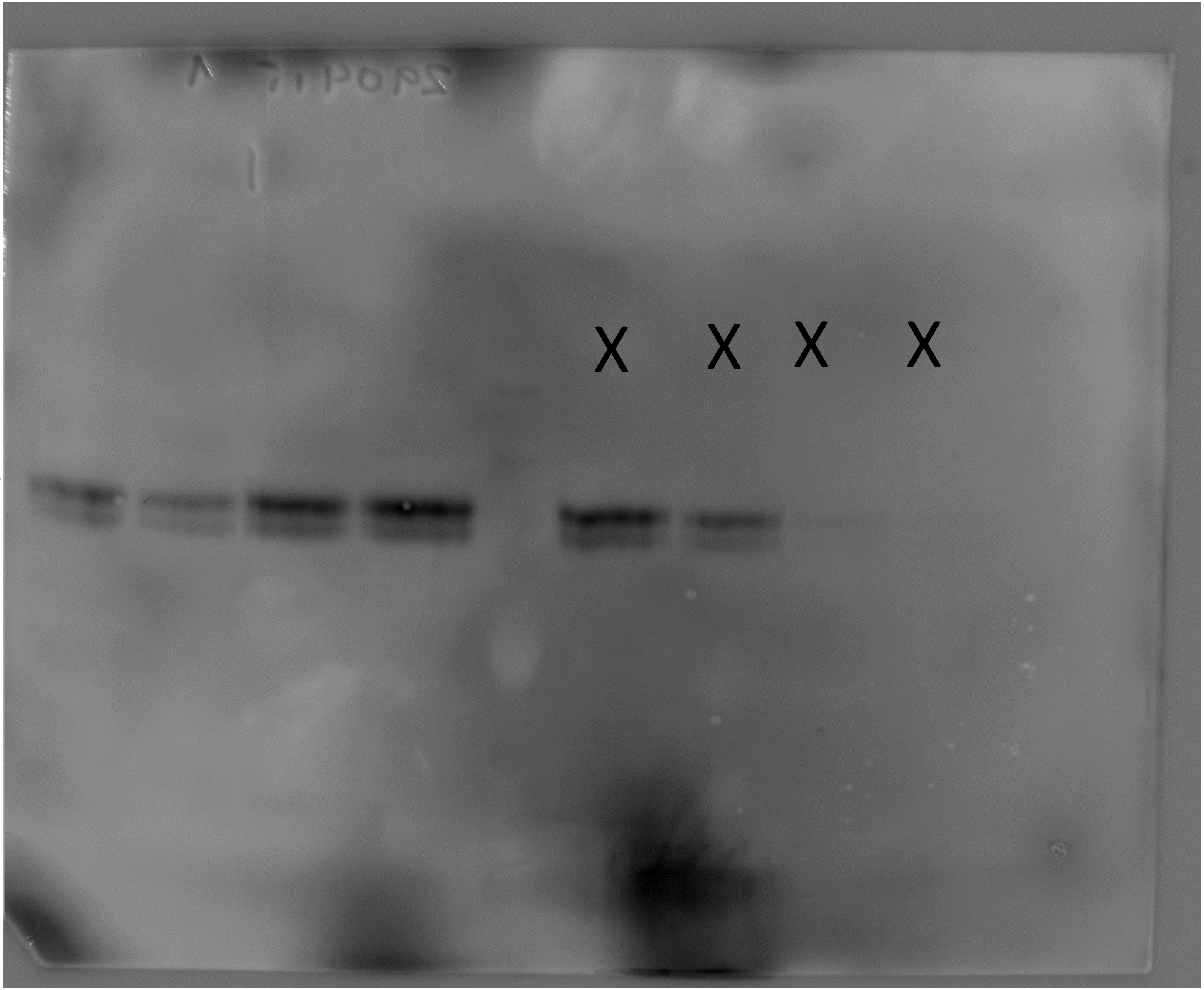

pIGF1R →

Detection method: EZ-ECL chemiluminescent detection HRP activity, automated

Fig 4B
n3

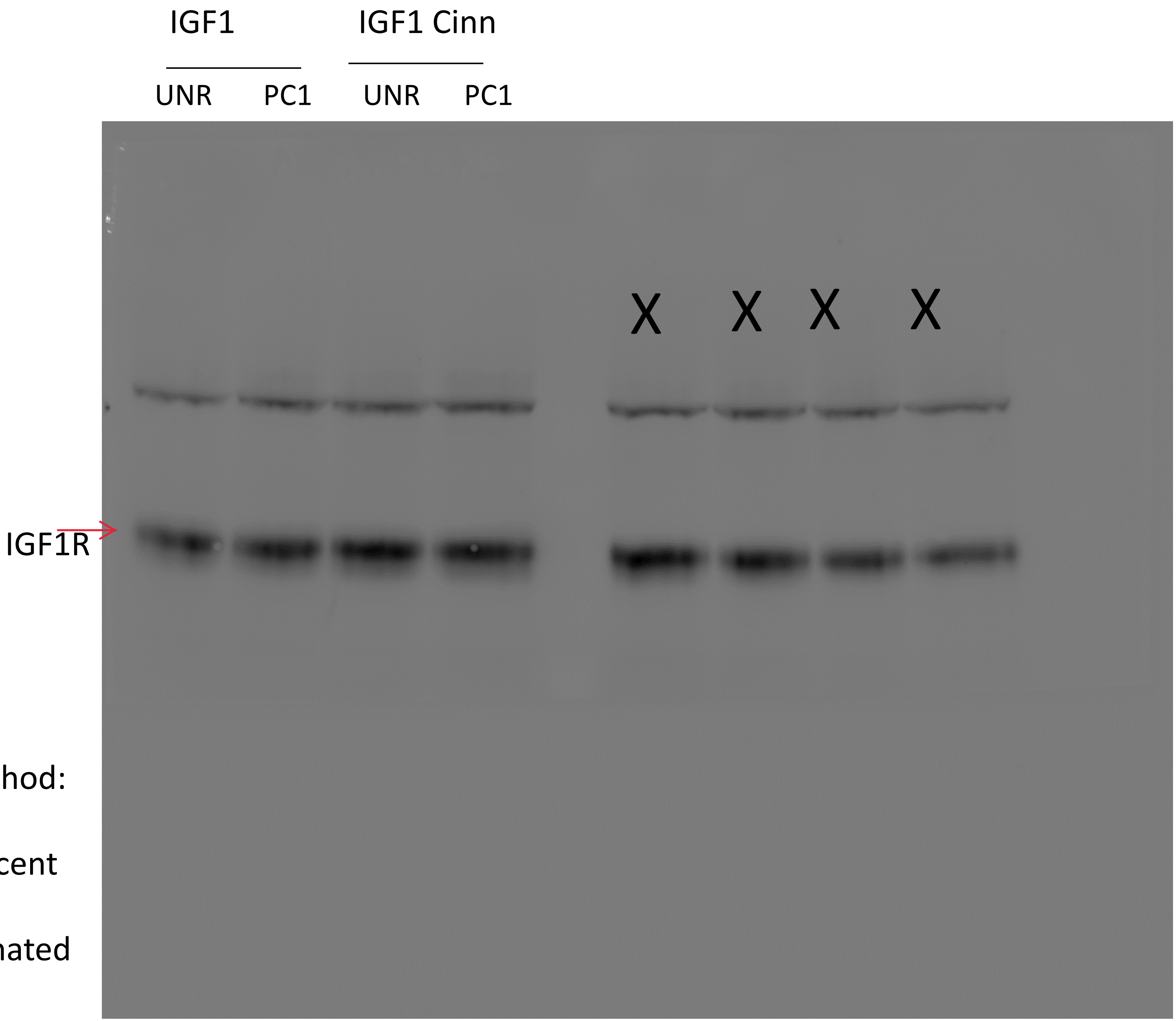

Detection method: EZ-ECL chemiluminescent detection HRP activity, automated

Fig 4B
n4

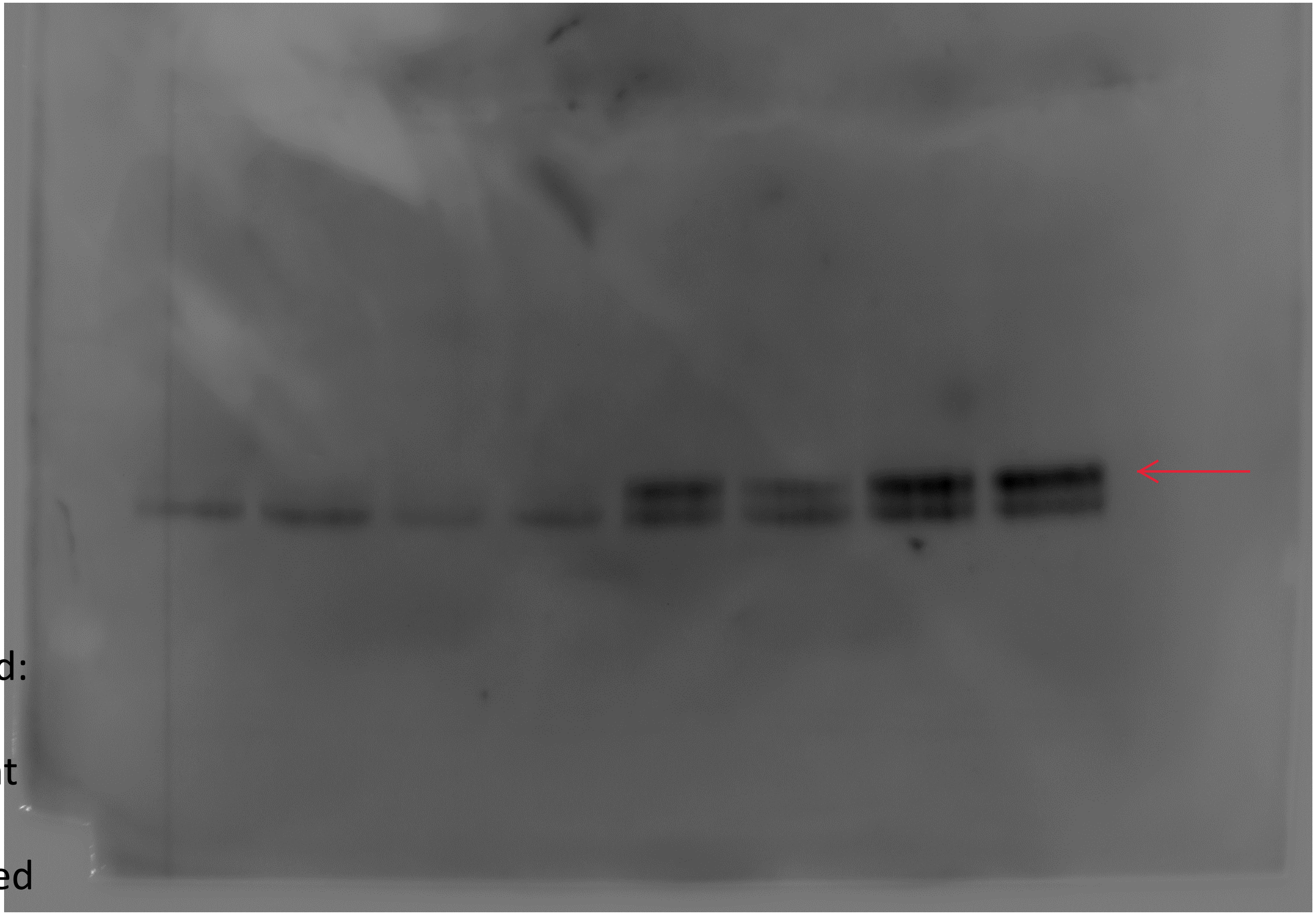
MM
MM Cinn
IGF1
IGF1 Cinn
UNR PC1 UNR PC1 UNR PC1 UNR PC1
pIGF1R
Detection method: EZ-ECL chemiluminescent detection HRP activity, automated

Fig 4B
n4

4B

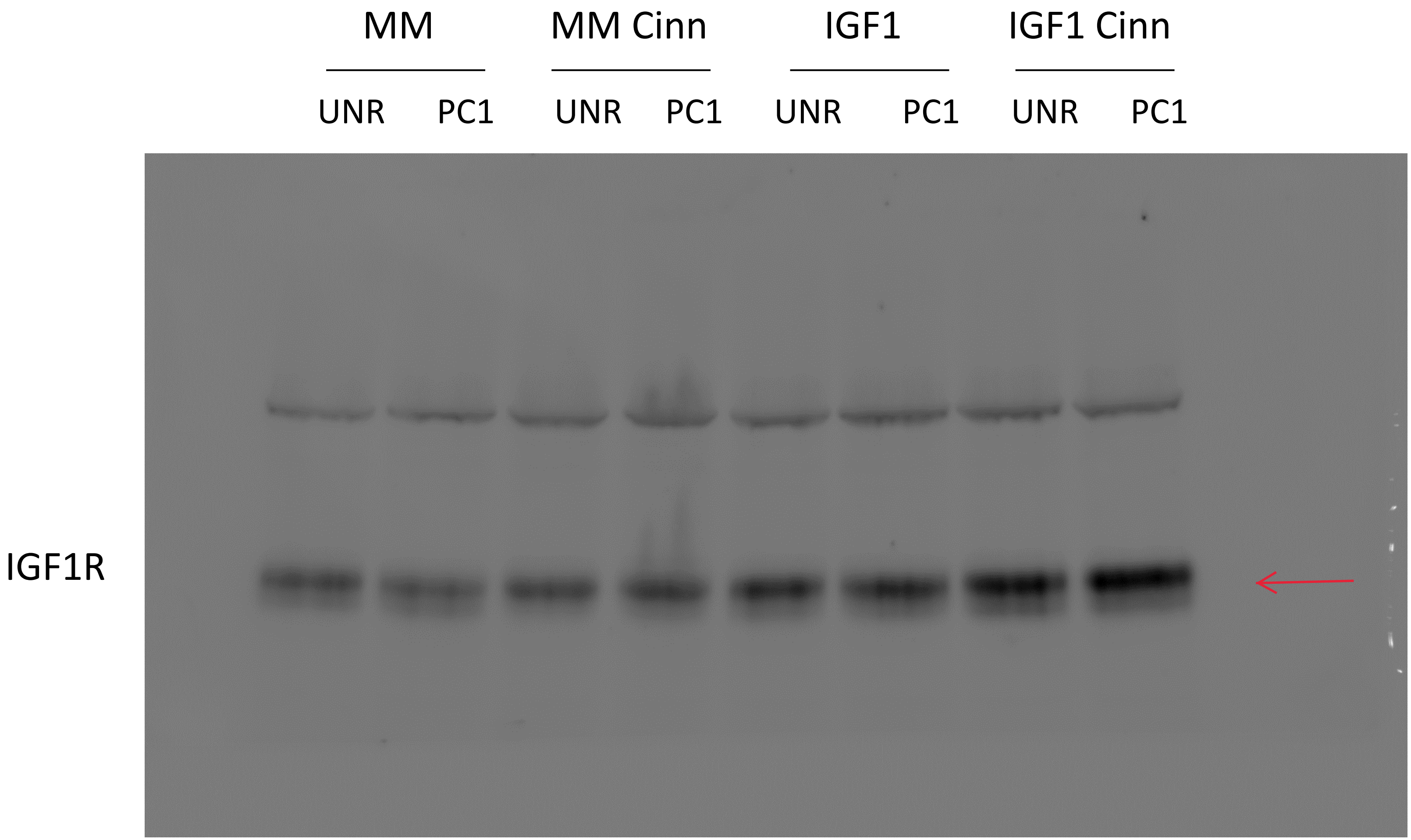

Detection method: EZ-ECL chemiluminescent detection HRP activity, automated

Fig 4C
n1
Paper figure

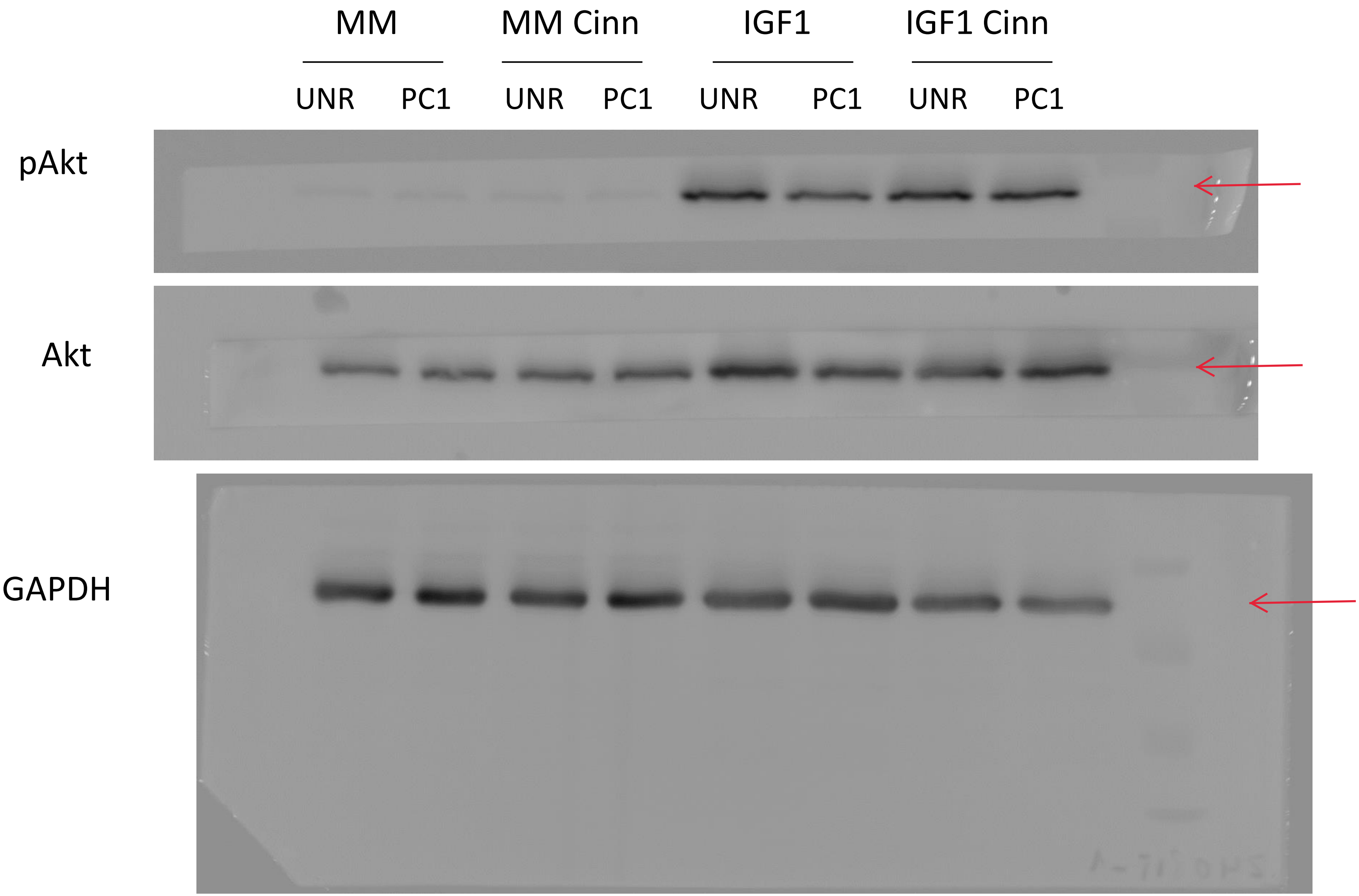

Detection method: EZ-ECL chemiluminescent detection HRP activity, automated

Fig 4C
n2

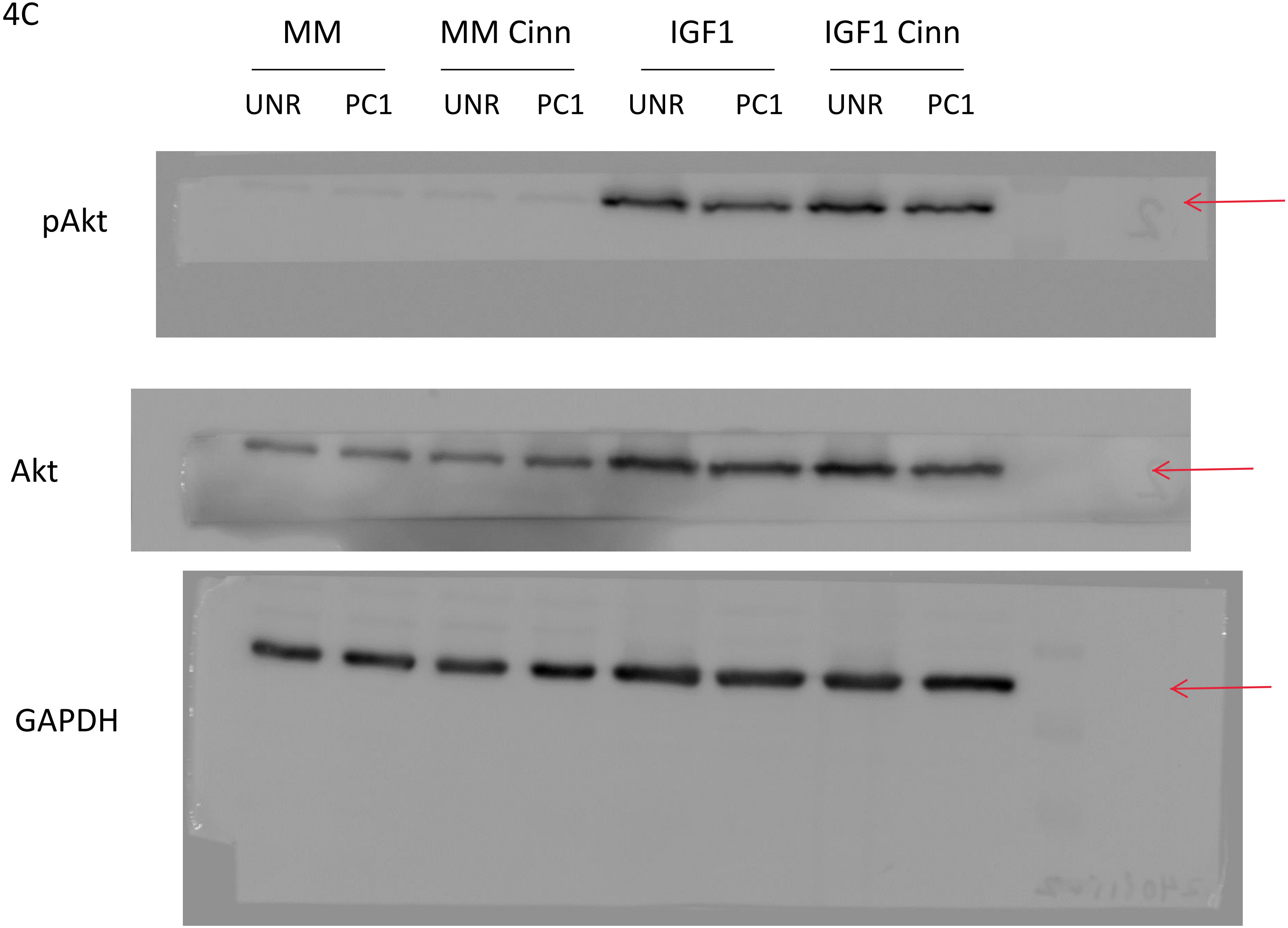

Detection method: EZ-ECL chemiluminescent detection HRP activity, automated

Fig 4C
n3

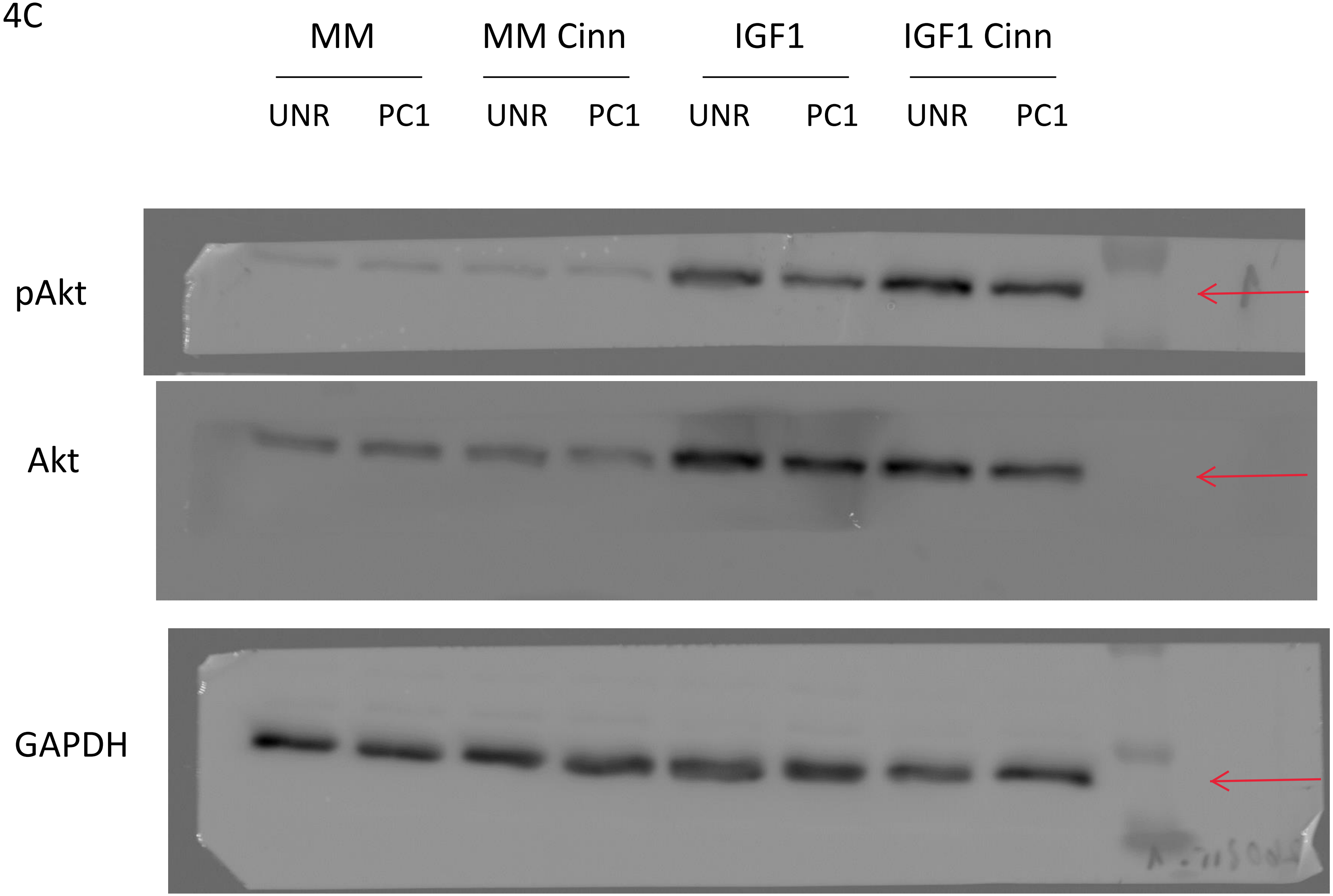

Detection method: EZ-ECL chemiluminescent detection HRP activity, automated

Fig 4C
n4

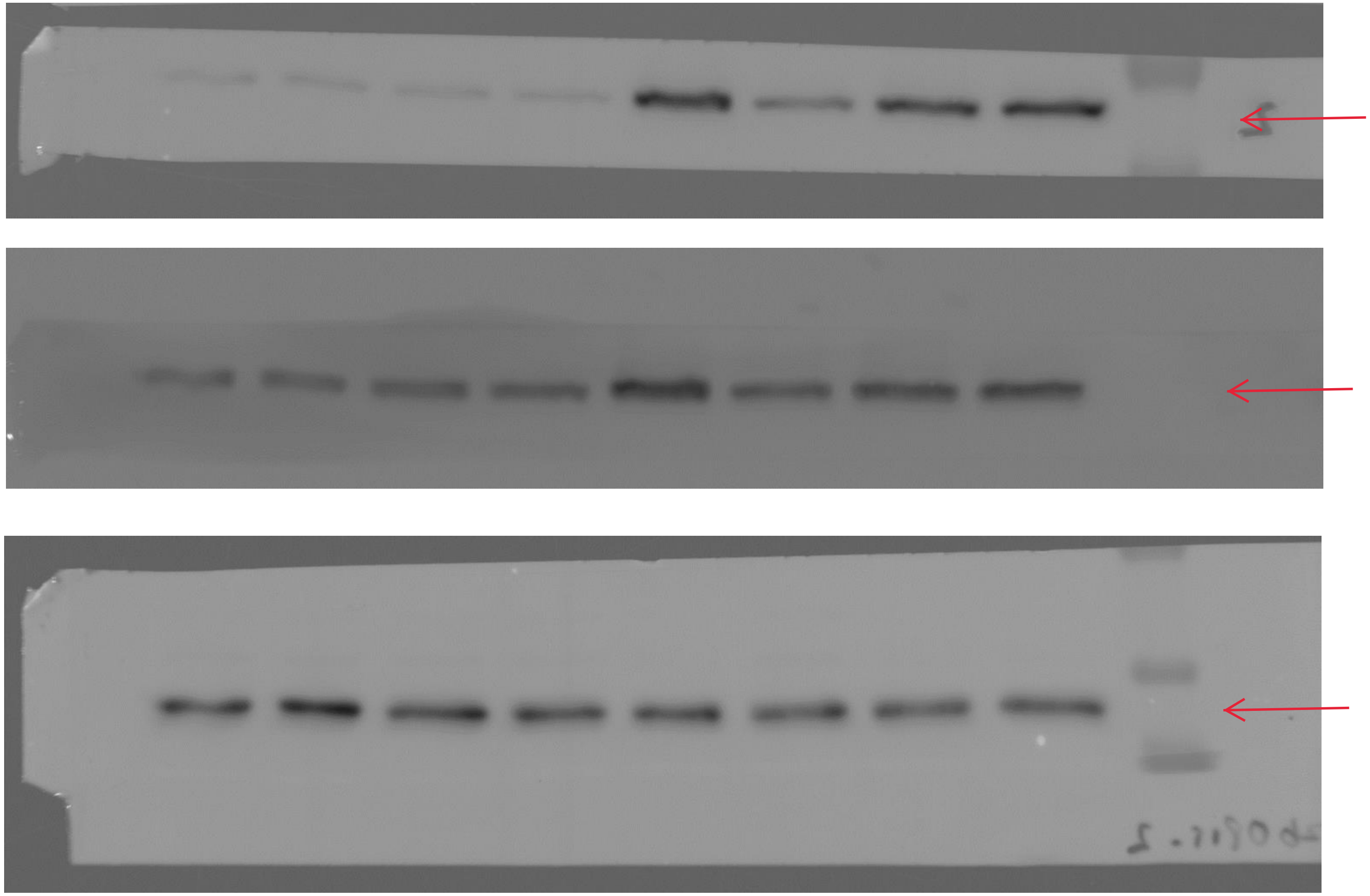

Detection method: EZ-ECL chemiluminescent detection HRP activity, automated

Fig 4D
n1

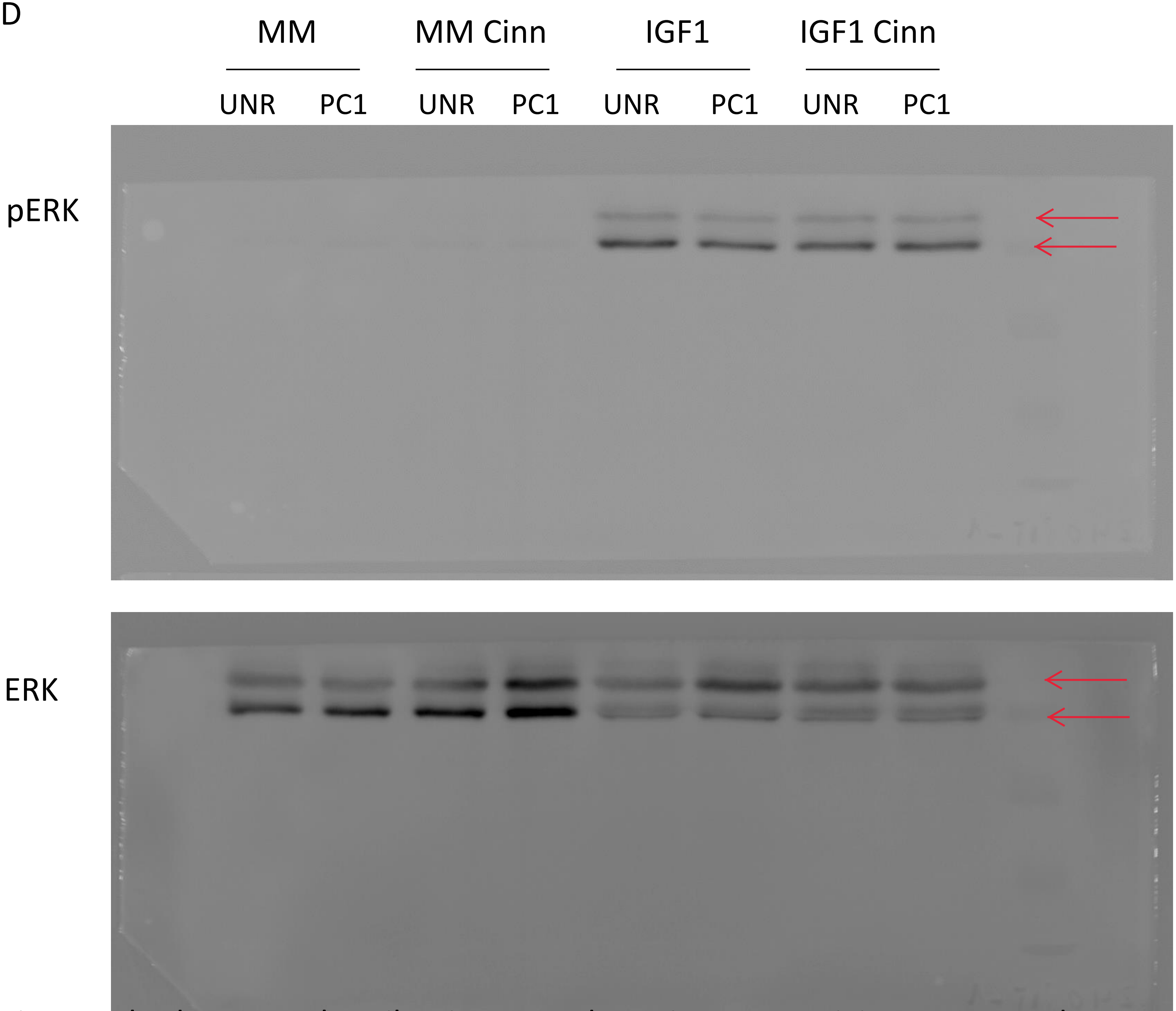

Detection method: EZ-ECL chemiluminescent detection HRP activity, automated

Fig 4D
n1

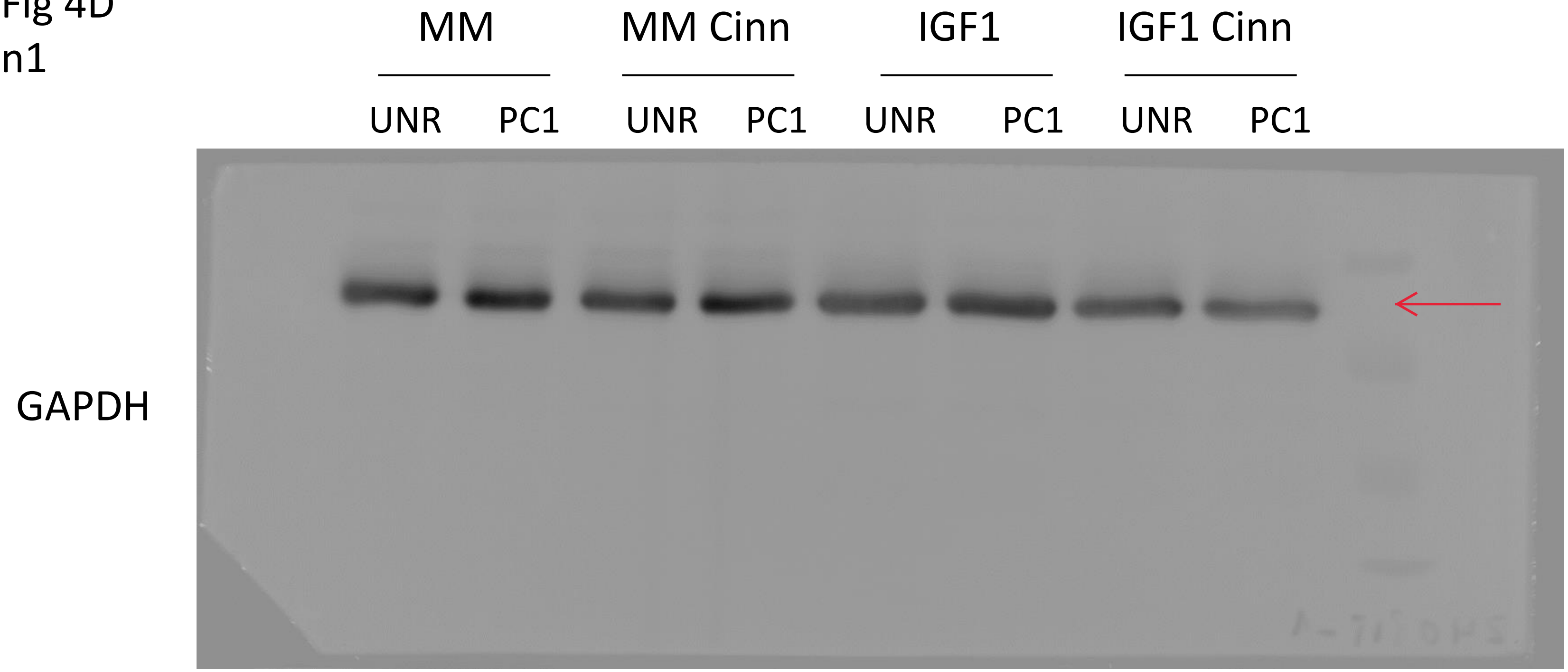

Detection method: EZ-ECL chemiluminescent detection HRP activity, automated

Fig 4D
n2

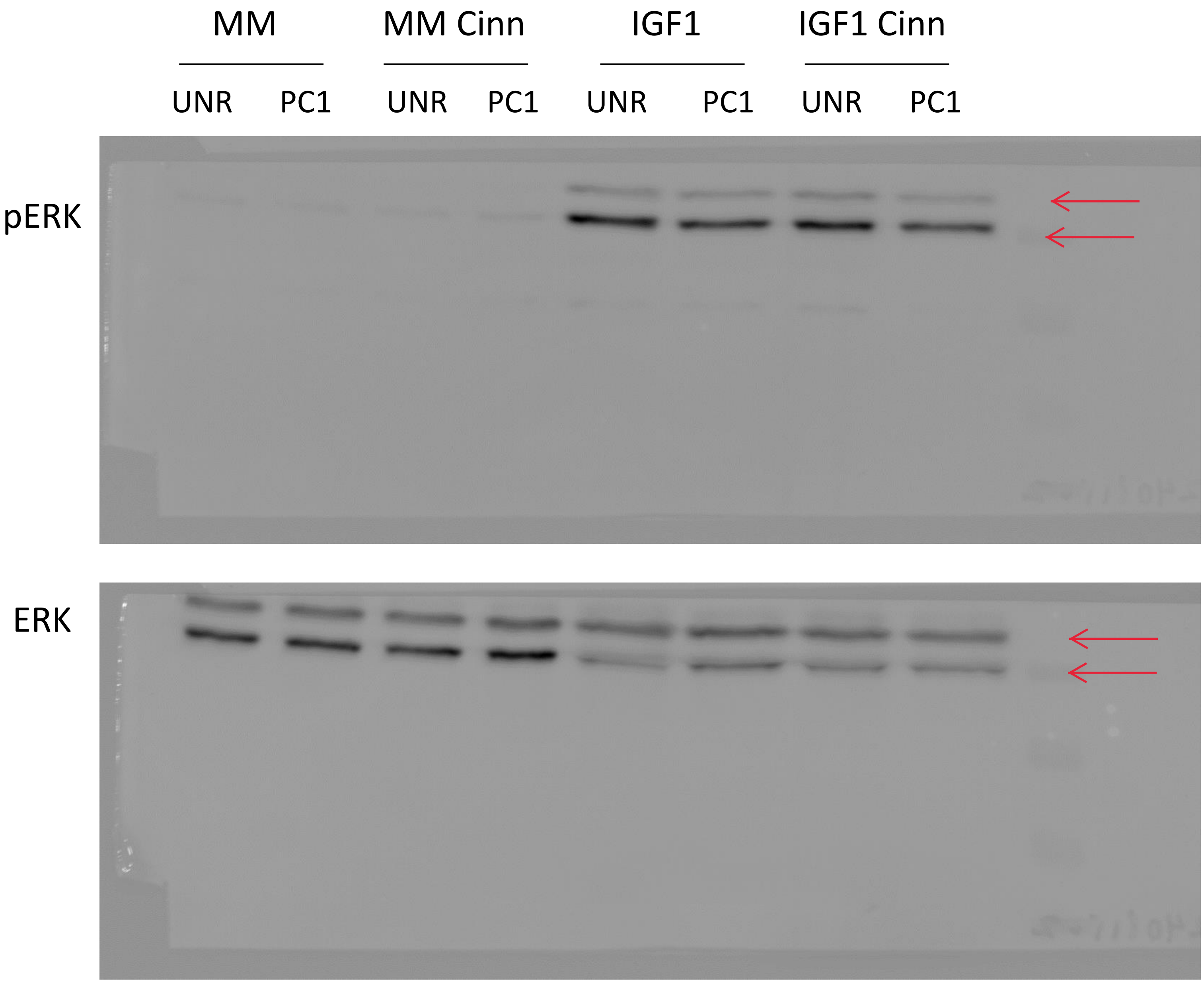

Detection method: EZ-ECL chemiluminescent detection HRP activity, automated

Fig 4D
n2

MM		MM Cinn		IGF1		IGF1 Cinn	
UNR	PC1	UNR	PC1	UNR	PC1	UNR	PC1

GAPDH

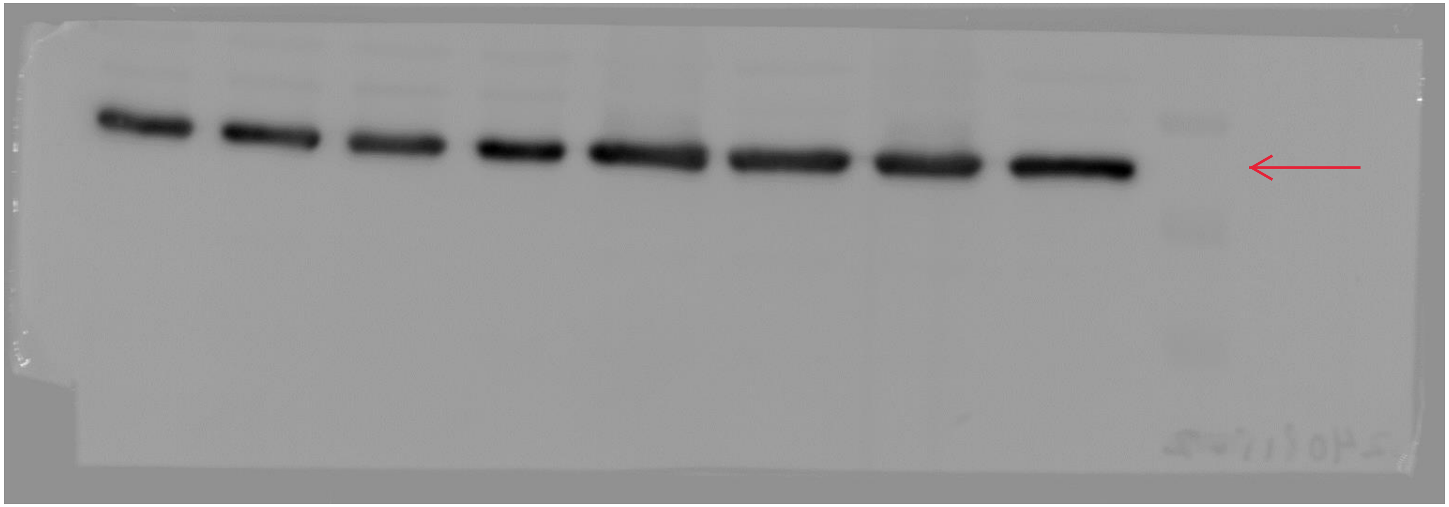

Detection method: EZ-ECL chemiluminescent detection HRP activity, automated

Fig 4D
n3

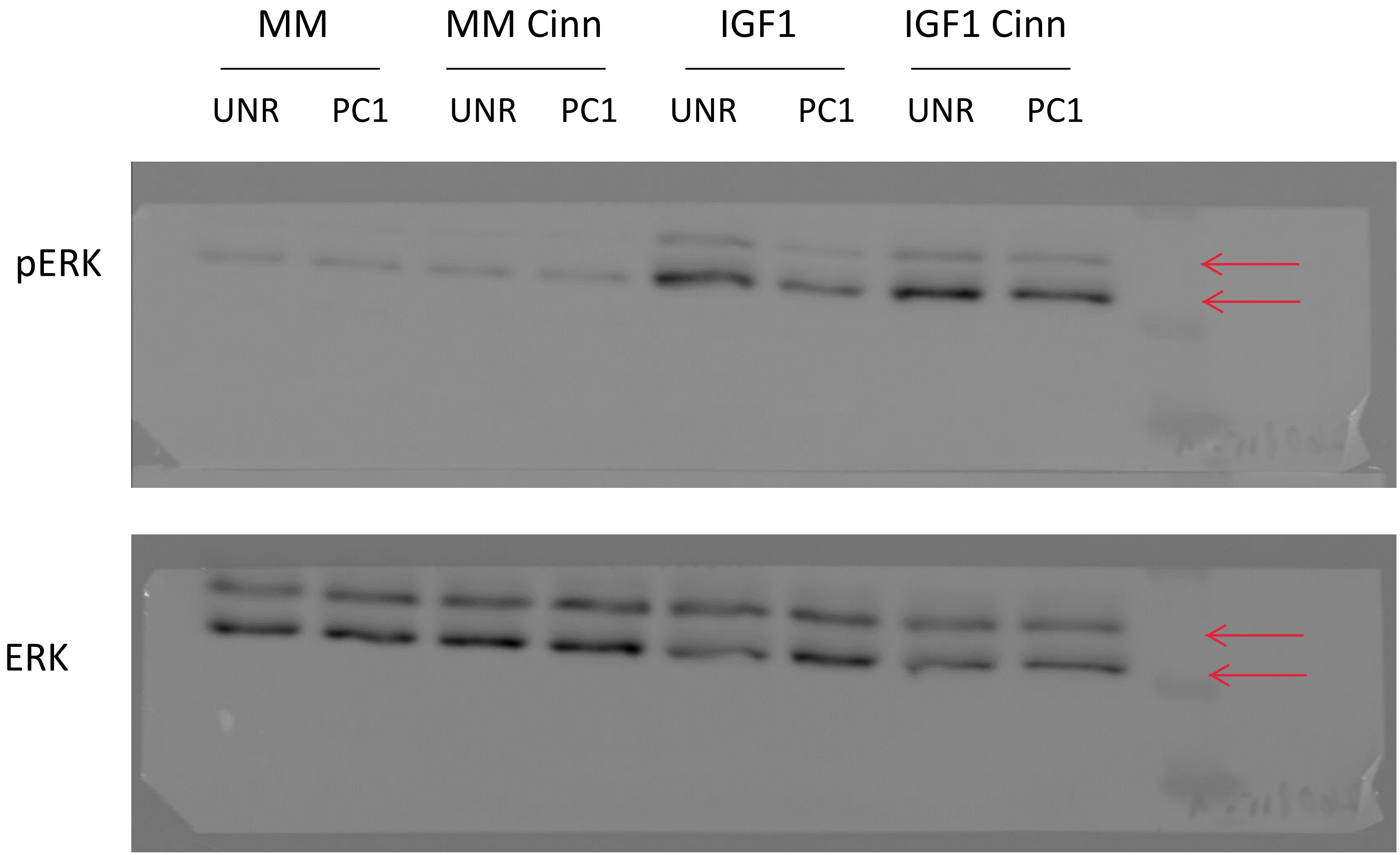

Detection method: EZ-ECL chemiluminescent detection HRP activity, automated

Fig 4D
n3

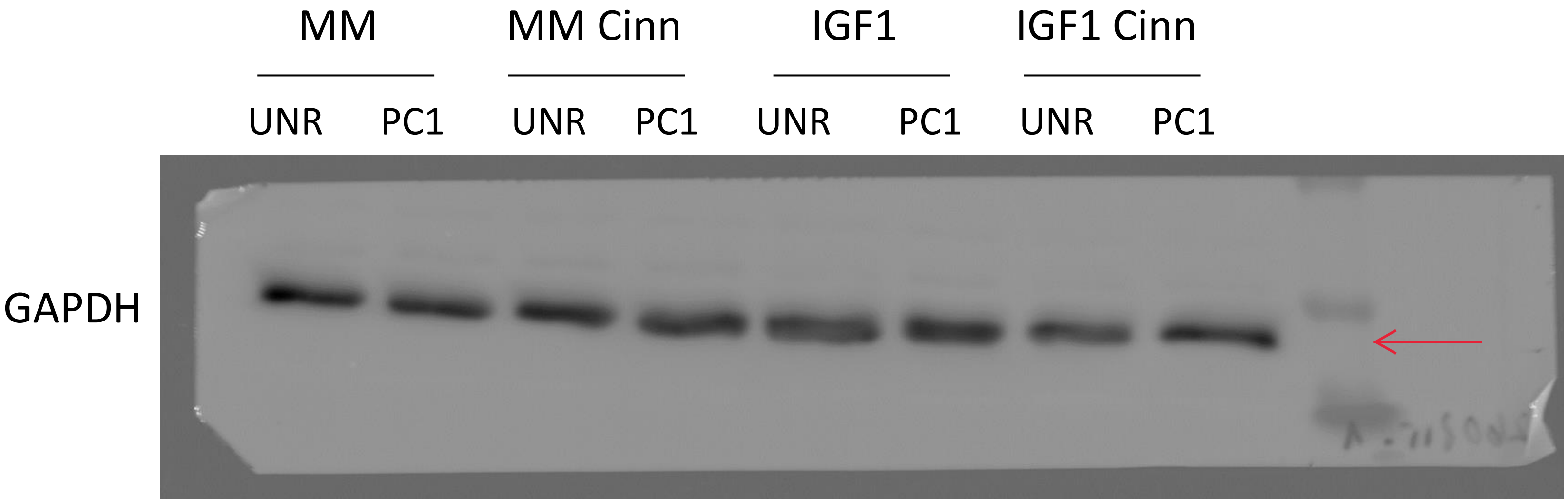

Detection method: EZ-ECL chemiluminescent detection HRP activity, automated

Fig 4D
n4
Paper figure

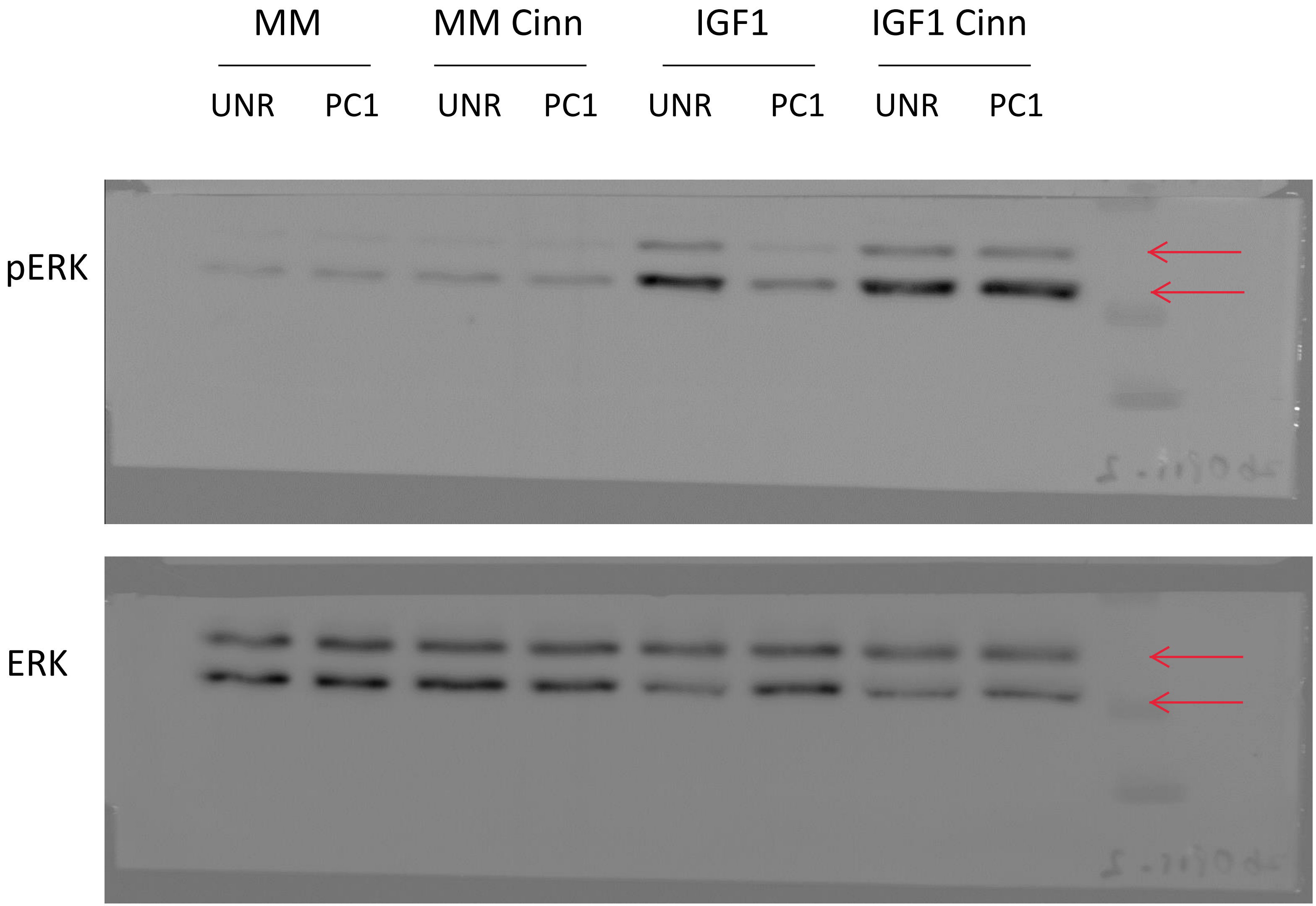

Detection method: EZ-ECL chemiluminescent detection HRP activity, automated

Fig 4D
n4
Paper figure

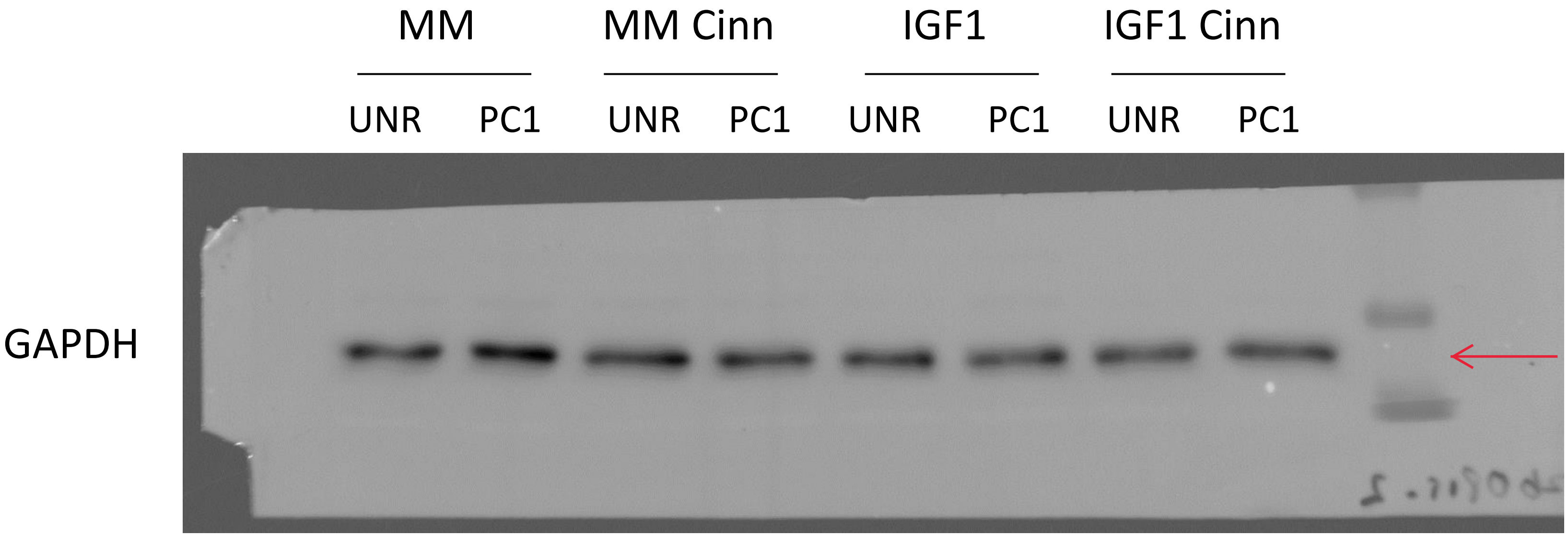

Detection method: EZ-ECL chemiluminescent detection HRP activity, automated
