# Supplementary material for: Polycystin-1 is required for insulin-like growth factor 1-induced cardiomyocyte hypertrophy
Source: PLoS One. 2021 Aug 18;16(8):e0255452. doi: 10.1371/journal.pone.0255452 (PMC8372926; doi:10.1371/journal.pone.0255452)
Supplement: S2 Fig — (PDF) [file pone.0255452.s002.pdf]

Fig 4A

n1

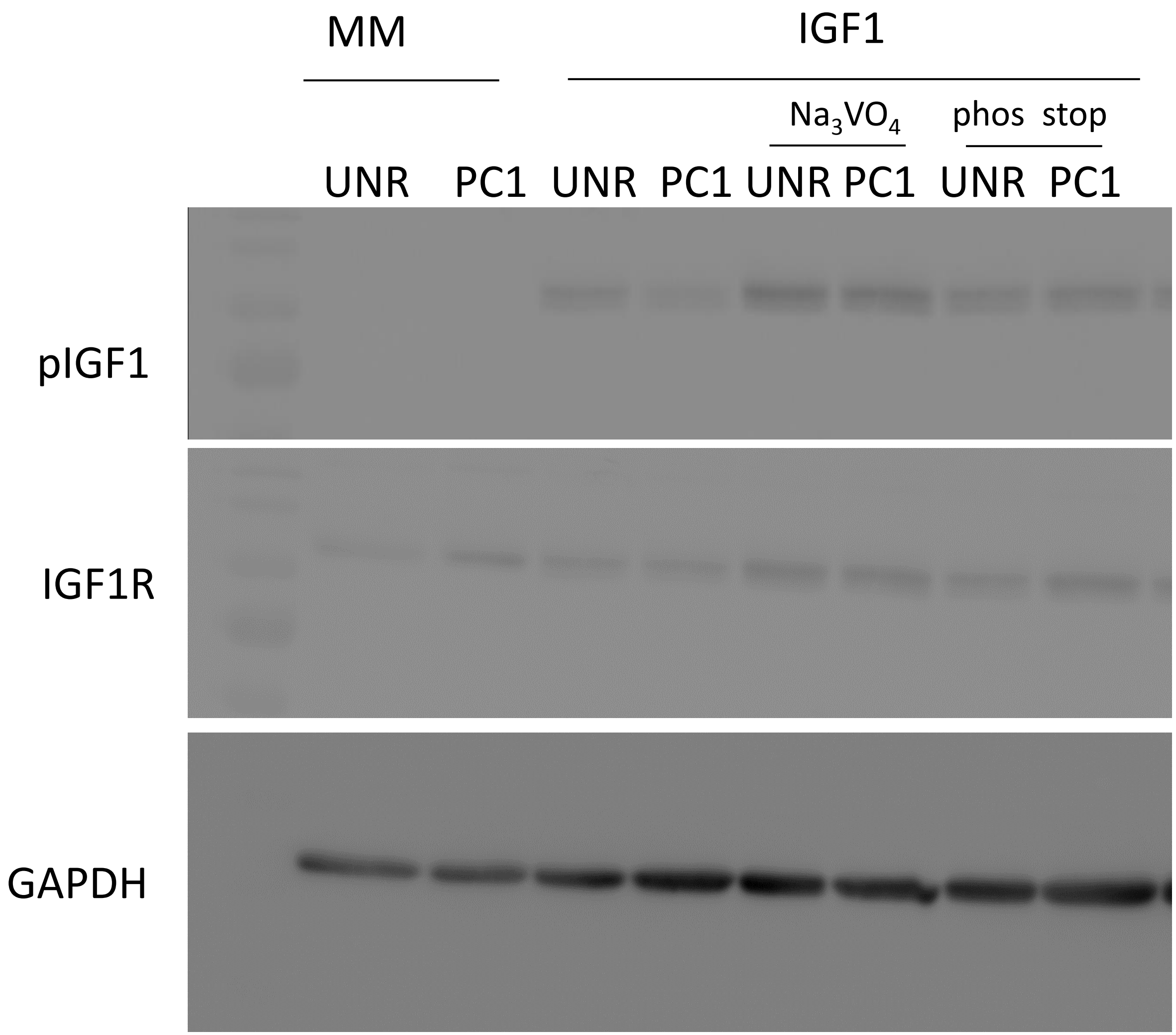
MM
IGF1
Na_3VO_4
phos stop
UNR
PC1
UNR
PC1
UNR
PC1
UNR
PC1
pIGF1
IGF1R
GAPDH

Fig 4A

Paper figure

n2

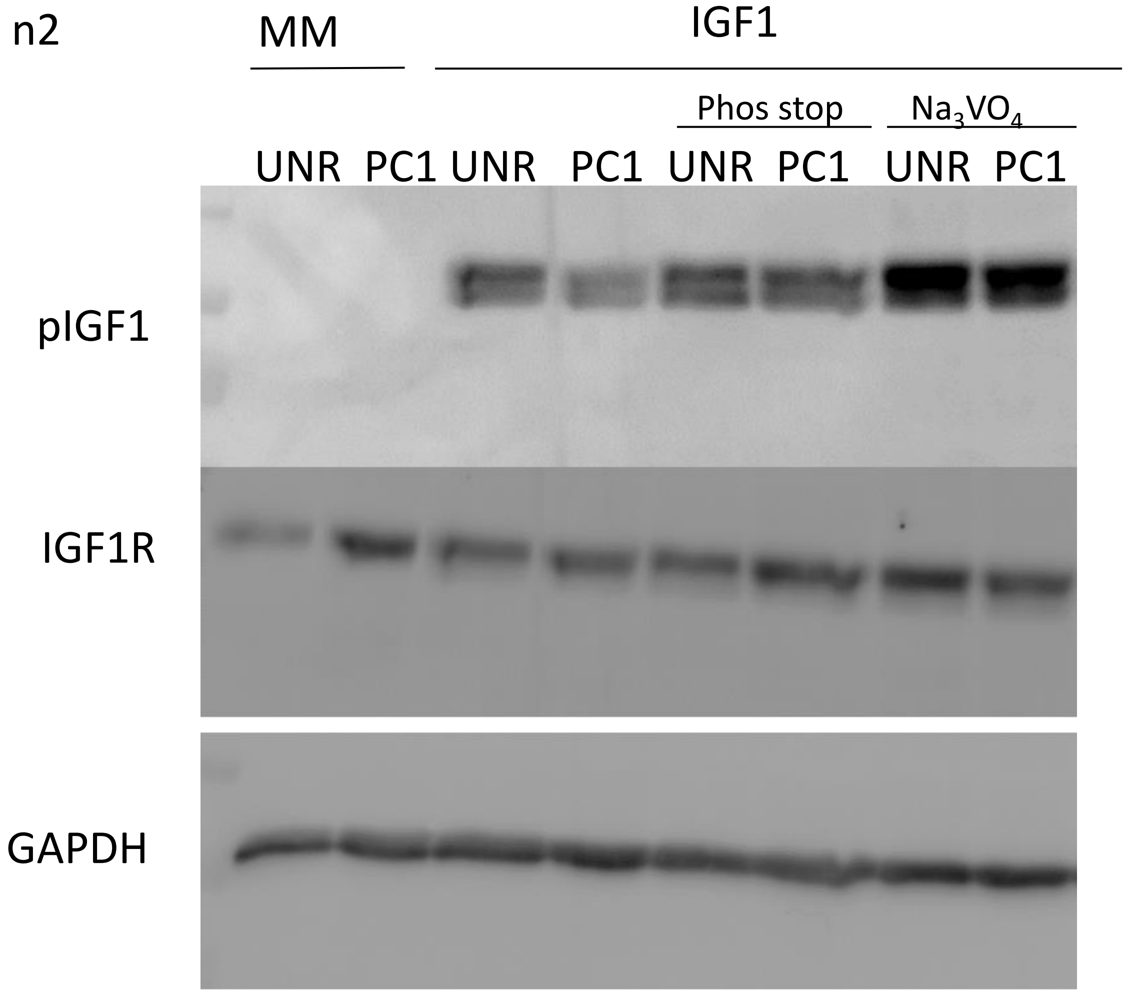

Fig 4A

n3

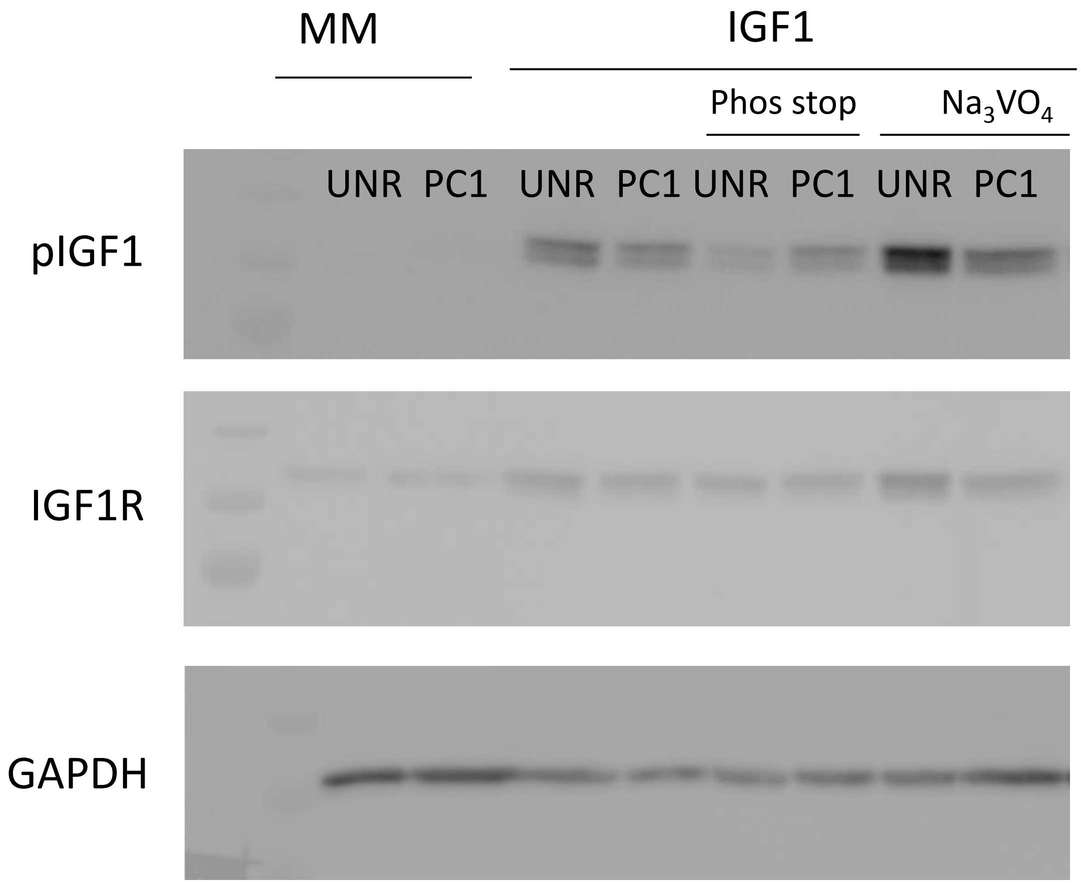
MM
IGF1
Phos stop
Na_3VO_4
UNR PC1 UNR PC1 UNR PC1 UNR PC1
pIGF1
IGF1R
GAPDH

Fig 4A

n4

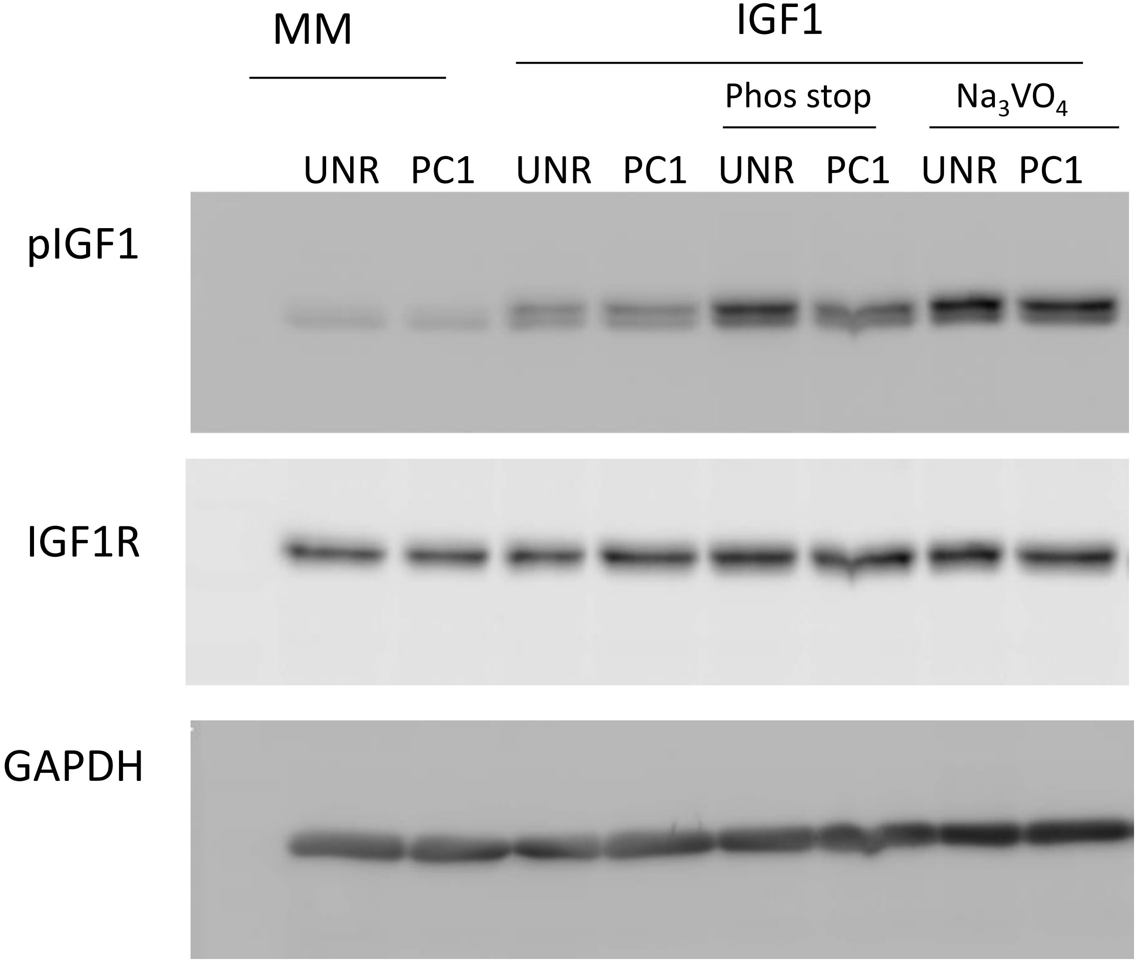

MM
IGF1
Phos stop
Na_3VO_4
UNR PC1 UNR PC1 UNR PC1 UNR PC1
pIGF1
IGF1R
GAPDH

Fig 4B
n1

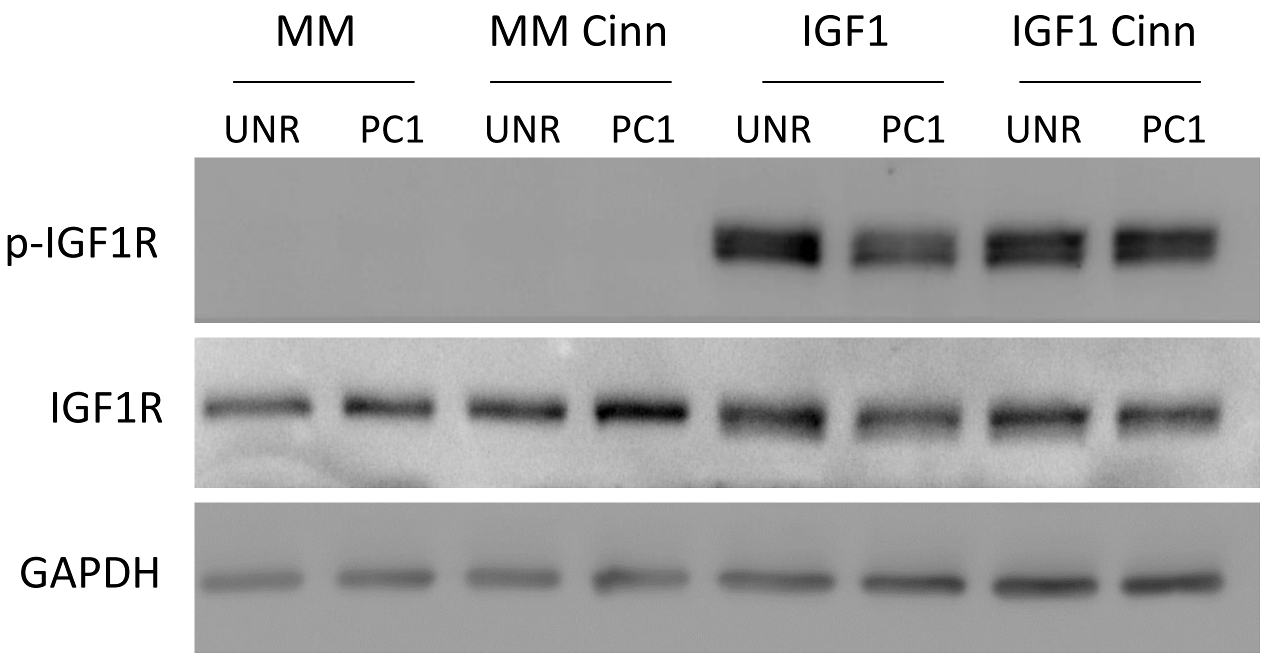

MM: control medium

Fig 4B
n2

Paper figure

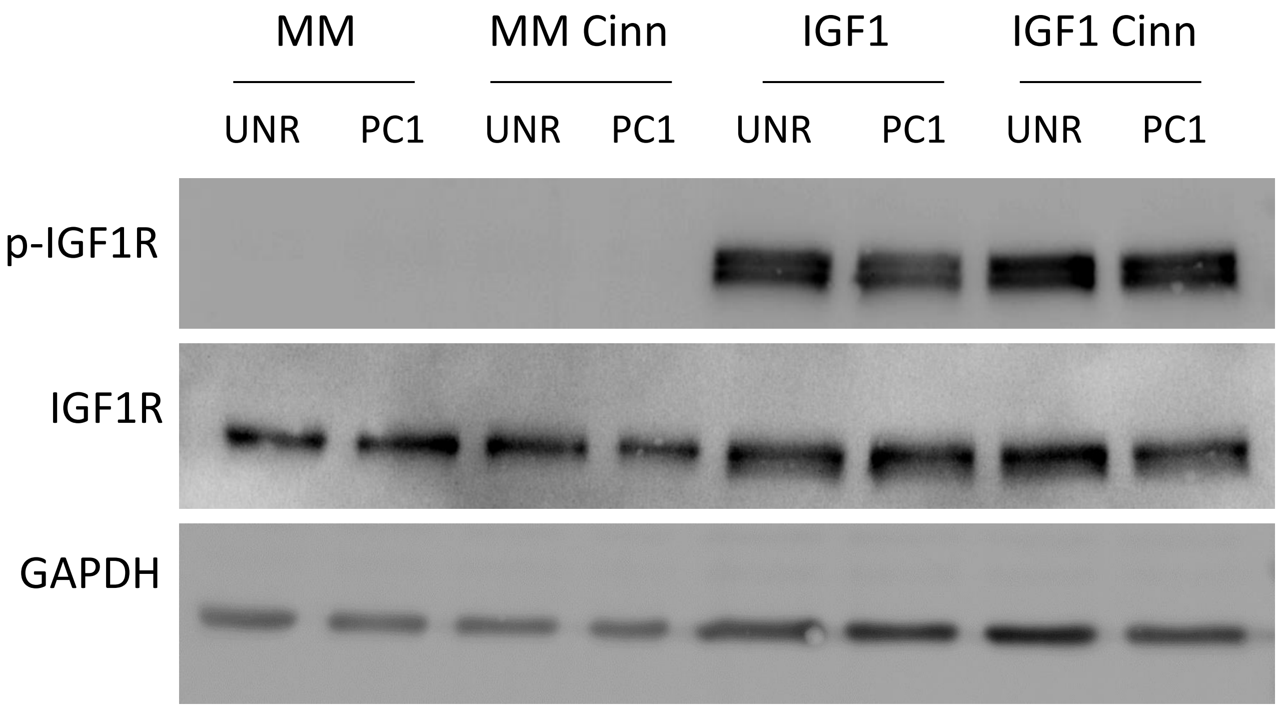

Fig 4B
n3

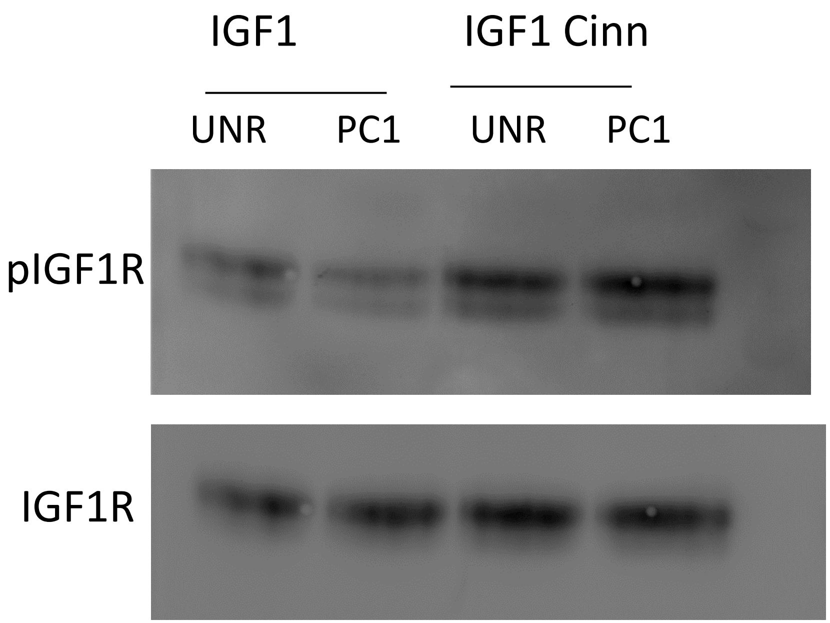

Fig 4B
n4

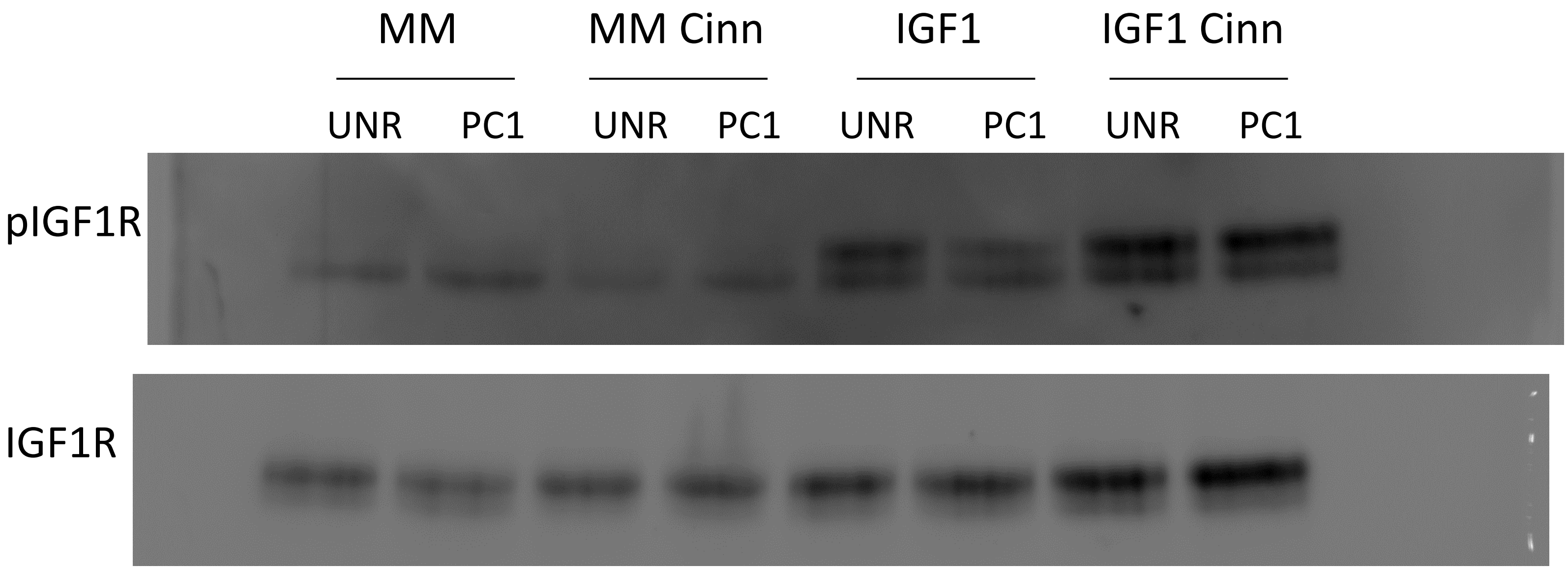
MM
MM Cinn
IGF1
IGF1 Cinn
UNR
PC1
UNR
PC1
UNR
PC1
UNR
PC1
pIGF1R
IGF1R

Fig 4C

n1

Paper figure

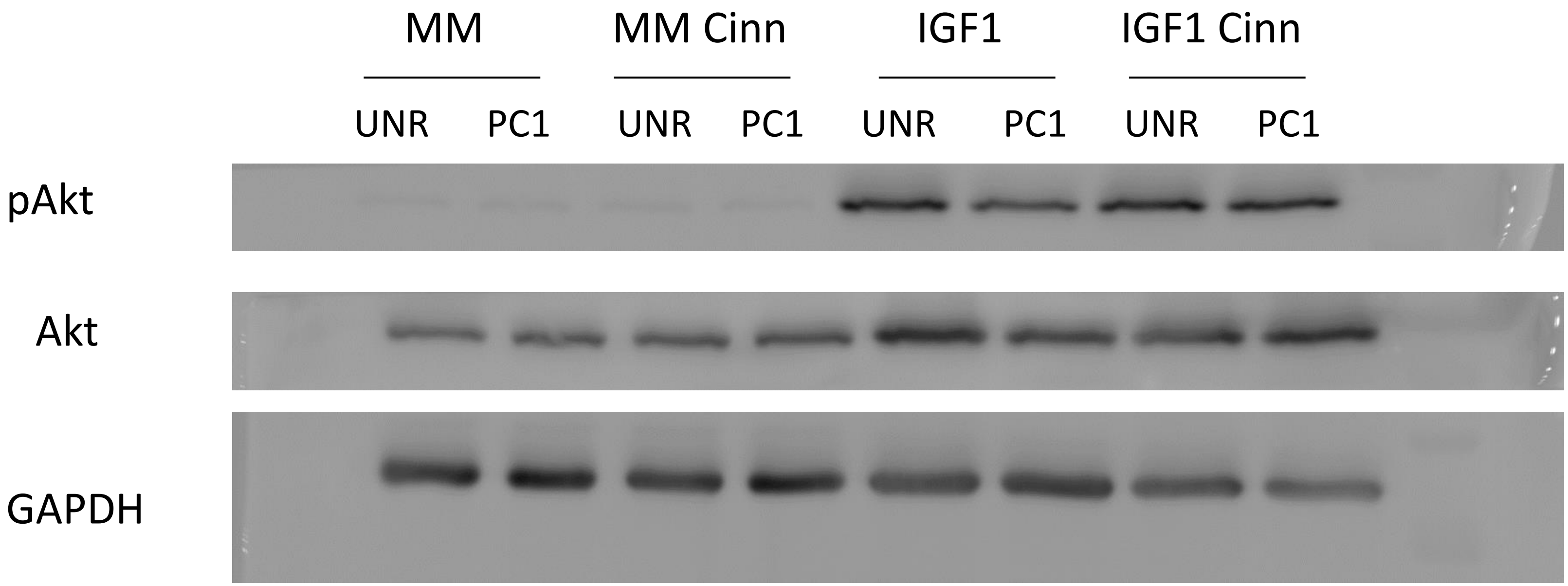

Fig 4C

n2

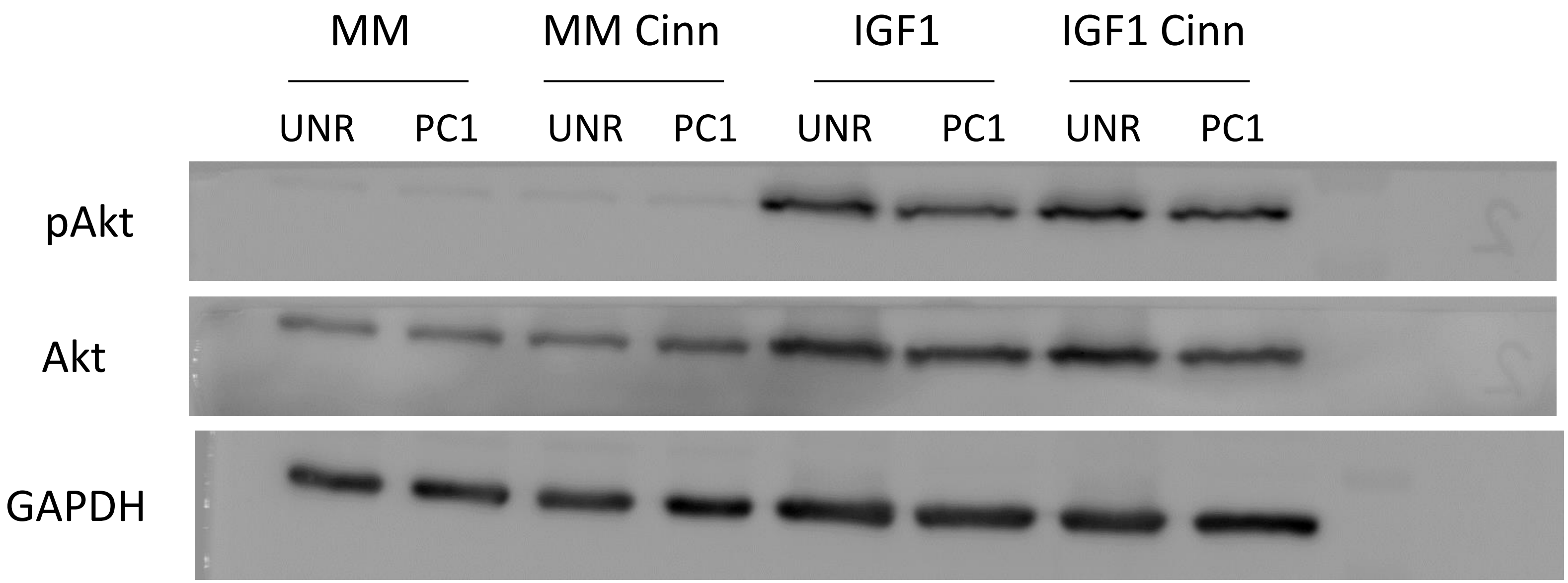
MM
MM Cinn
IGF1
IGF1 Cinn
UNR
PC1
UNR
PC1
UNR
PC1
UNR
PC1
pAkt
Akt
GAPDH

Fig 4C

n3

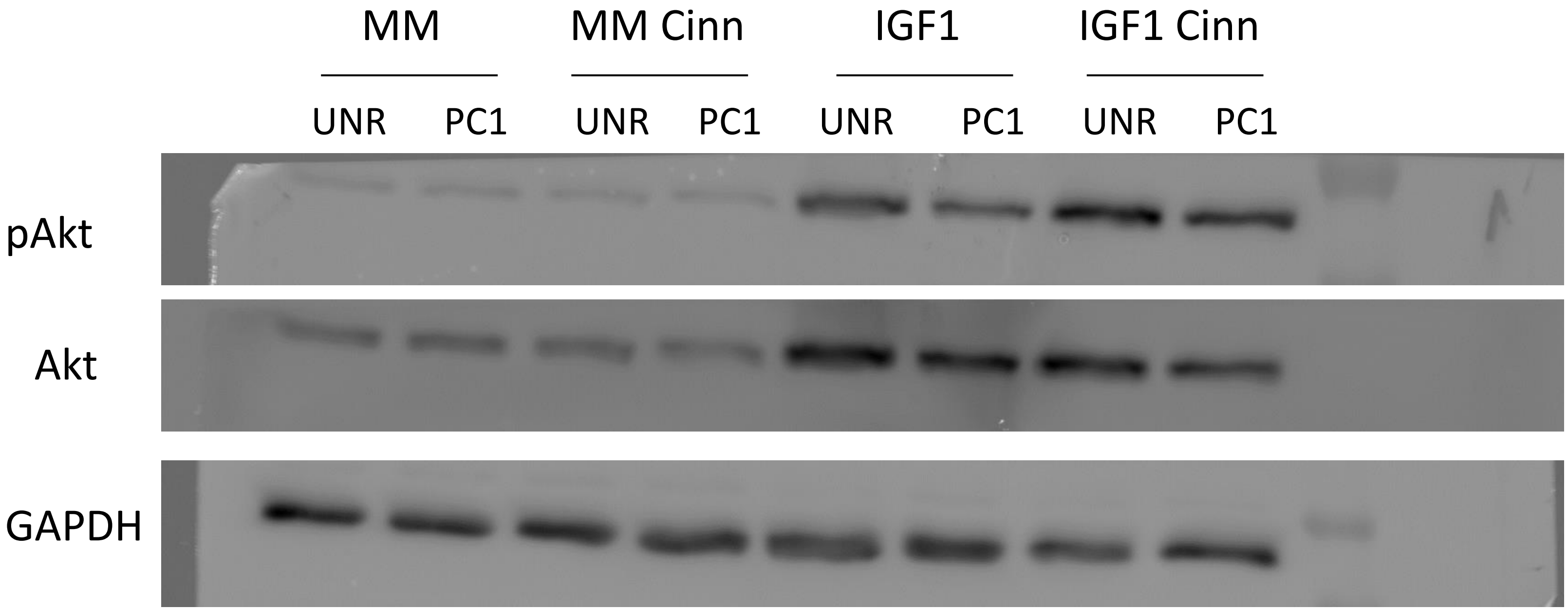
MM
MM Cinn
IGF1
IGF1 Cinn
UNR
PC1
UNR
PC1
UNR
PC1
UNR
PC1
pAkt
Akt
GAPDH

Fig 4C

n4

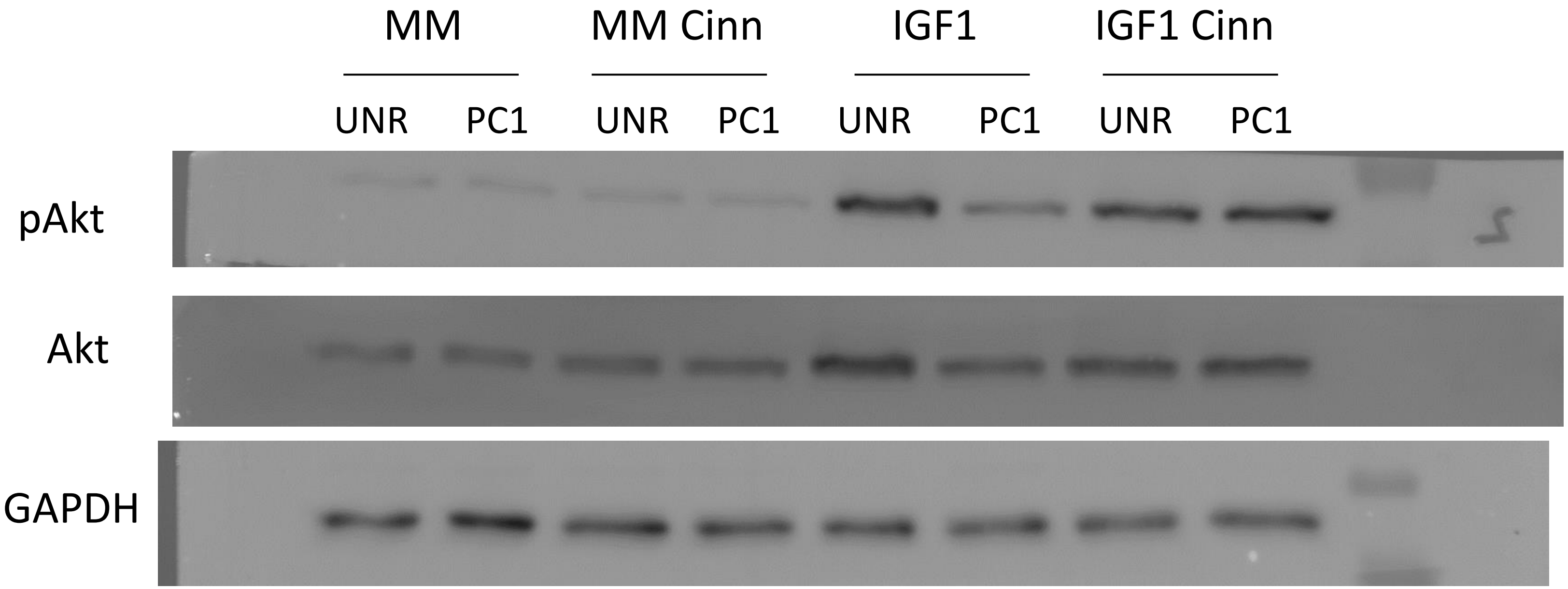
MM
MM Cinn
IGF1
IGF1 Cinn
UNR
PC1
UNR
PC1
UNR
PC1
UNR
PC1
pAkt
Akt
GAPDH

Fig 4D

n1

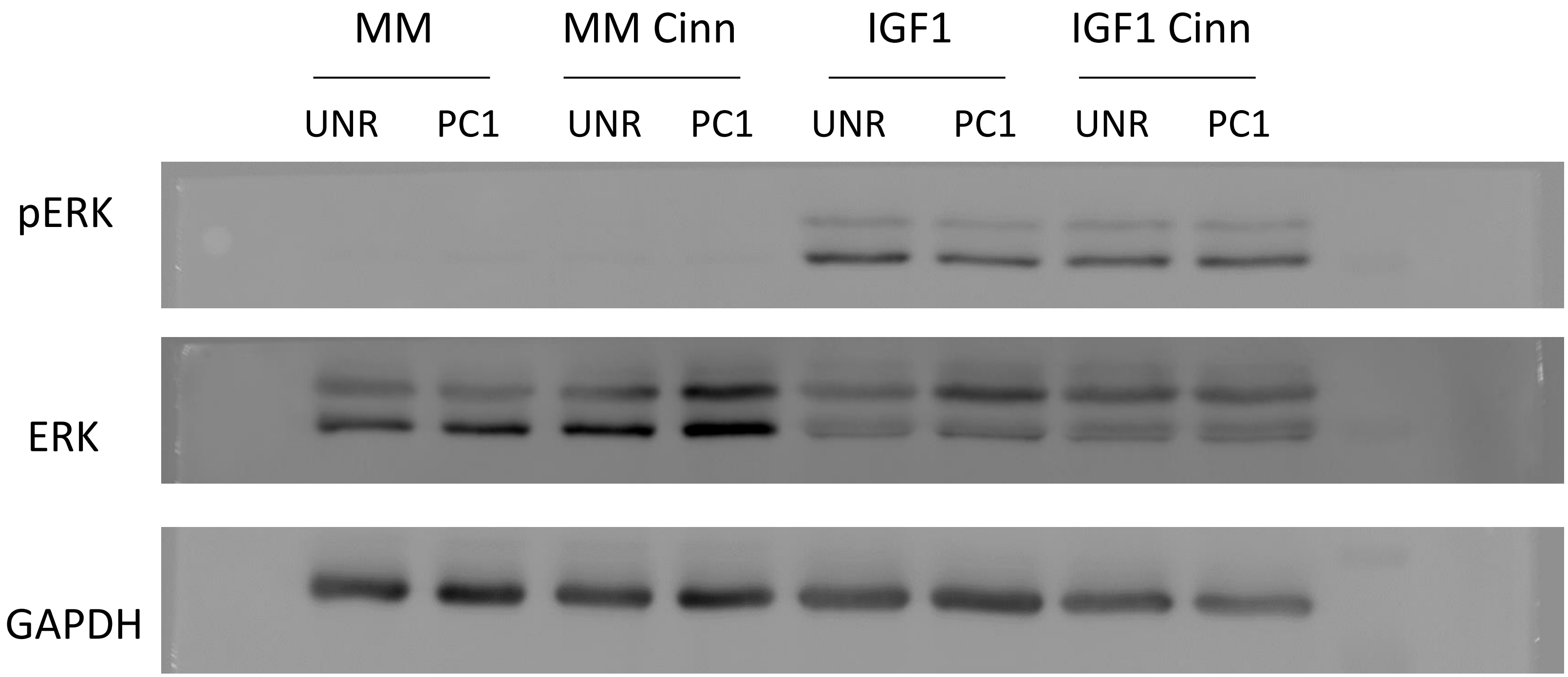
MM
MM Cinn
IGF1
IGF1 Cinn
UNR PC1 UNR PC1 UNR PC1 UNR PC1
pERK
ERK
GAPDH

Fig 4D

n2

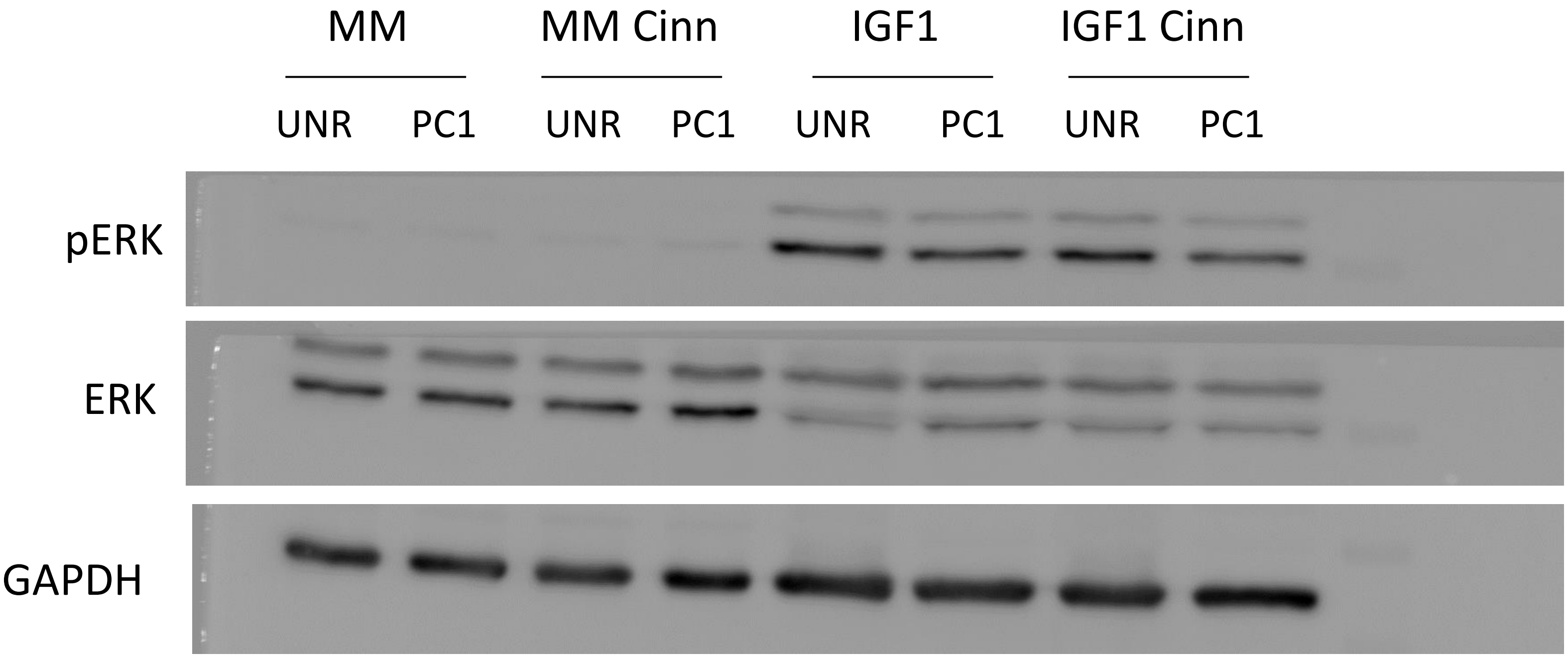
MM
MM Cinn
IGF1
IGF1 Cinn
UNR
PC1
UNR
PC1
UNR
PC1
UNR
PC1
pERK
ERK
GAPDH

Fig 4D

n3

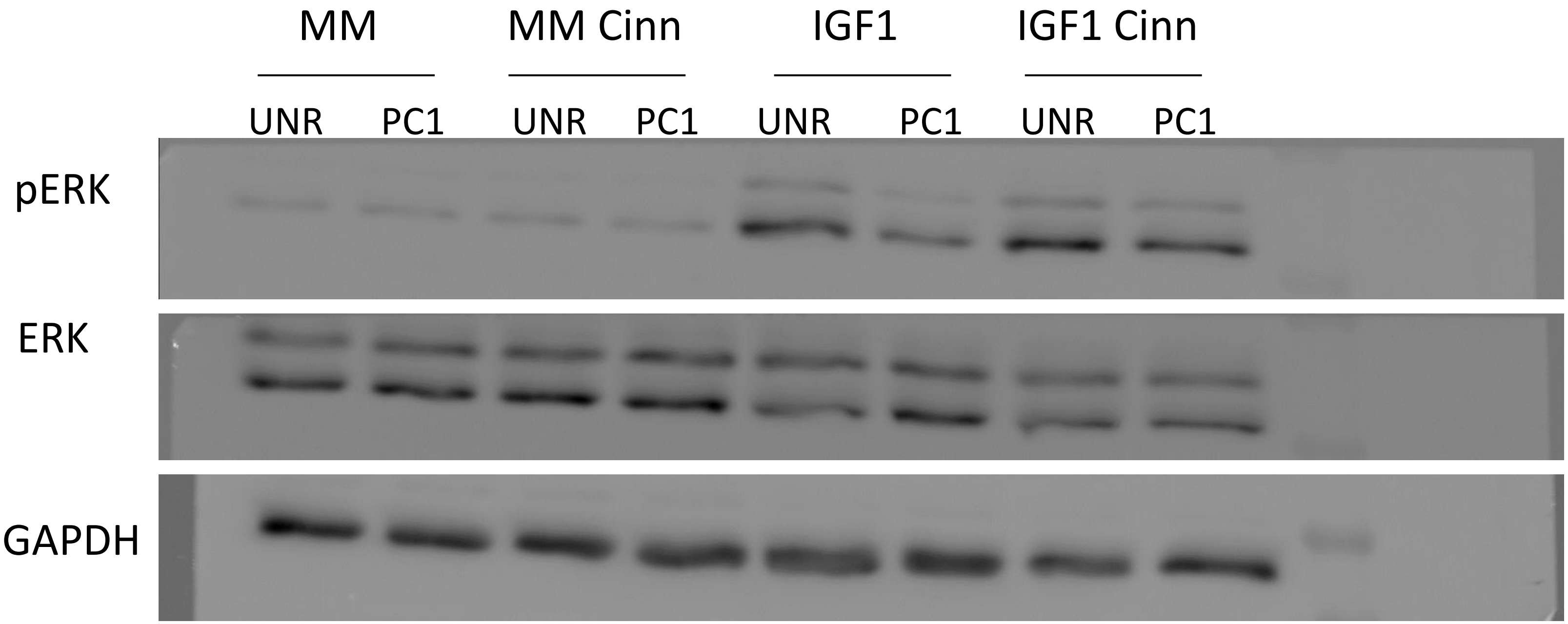
MM
MM Cinn
IGF1
IGF1 Cinn
UNR
PC1
UNR
PC1
UNR
PC1
UNR
PC1
pERK
ERK
GAPDH

Fig 4D

n4

Paper figure

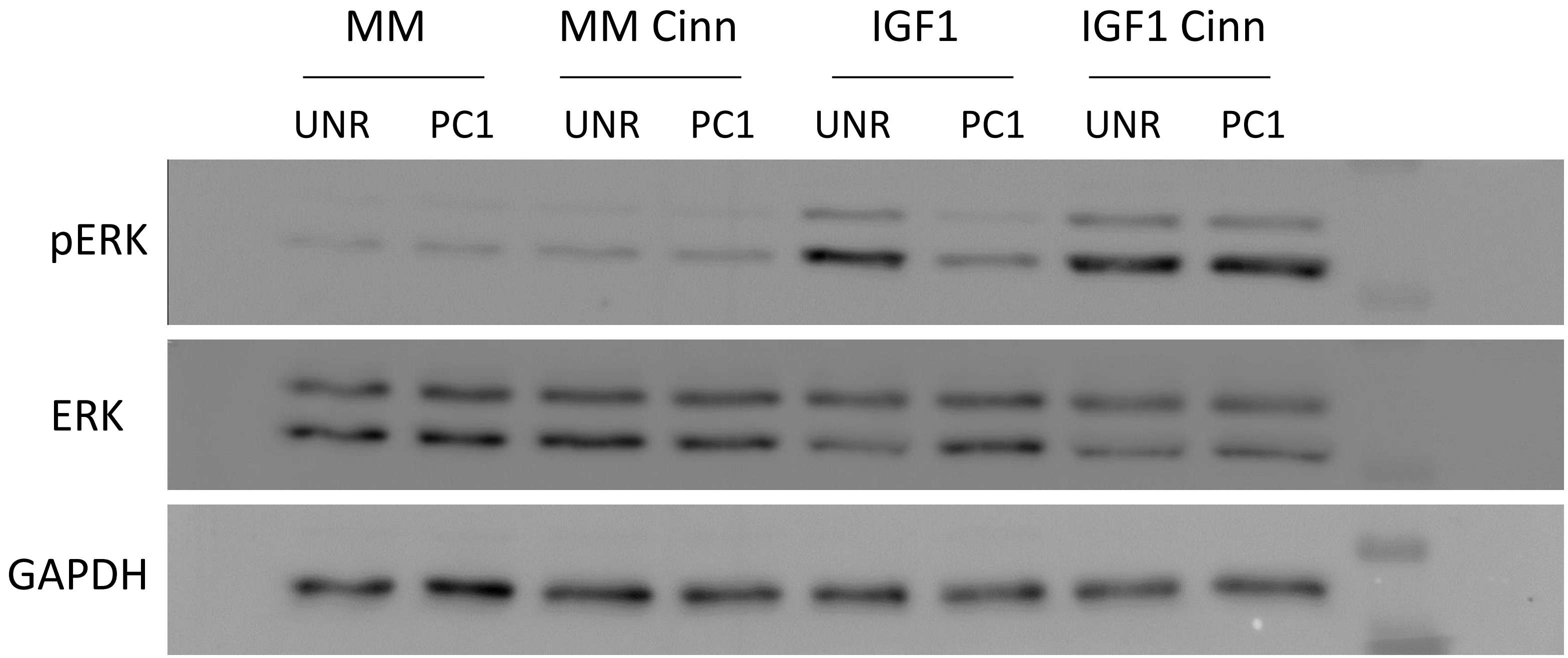

Controls fig 5 A

BNP mRNA fold increase UNR MM	UNR MM	siPC1 MM	UNR MM cinn	siPC1 MM cinn	UNR IGF1	siPC1 IGF1	UNR IGf1 cinn	siPC1 IGF1 cinn
n1	1,00	0,29	0,80	0,06	2,52		2,30	2,77
n2	1,00	1,19	0,49	1,18	1,23	0,95	3,33	2,53
n3	1,00	0,61	1,51	0,64	1,69	0,91	2,17	2,45
n4	1,01	1,37	0,42	1,21	1,94	0,78	1,41	2,14

Controls fig 5 D and E

All data								
area	UNR MM	siPC1 MM	UNR MM cinn	siPC1 MM cinn	UNR IGf1	siPC1 IGf1	UNR IGf1 Cinn	siPC1 IGf1 Cinn
n1	1	1,12657469	0,975745468	0,849367987	1,590304	0,75274441	1,460139908	1,764228888
n2	1	1,01349077	1,166918603	1,279014572	1,72975004	0,9323638	1,79119188	1,660656829
n3	1	1,087454	0,972915961	0,974013834	2,26002014	0,82909298	2,189268662	2,489261232
n4	1				1,80750892	0,93821742	1,789717644	1,711204316
perimeter	UNR MM	siPC1 MM	UNR MM cinn	siPC1 MM cinn	UNR IGf1	siPC1 IGf1	UNR IGf1 Cinn	siPC1 IGf1 Cinn
n1	1	1,01170363	0,888108893	1,011703625	1,40840643	0,84974715	1,40179156	1,493989986
n2	1	1,00220419	1,058017775	1,079212236	1,34538031	0,98738132	1,408929012	1,447887052
n3	1	1,07416745	1,103234295	0,97737293	1,59624617	0,91649453	1,553029553	1,536826267
n4	1				1,68869495	0,98681454	1,497885942	1,608417123
